# Highly diverse endophytic fungi from Serra do Amolar-Pantanal (Brazil) producing bioactive secondary metabolites against phytopathogens

**DOI:** 10.3389/fmicb.2024.1501182

**Published:** 2024-12-24

**Authors:** Bárbara Fanaya Mayrhofer, Jucélia Iantas, Sandriele Aparecida Noriler, Larissa V. Ponomareva, Jon S. Thorson, Jürgen Rohr, Khaled A. Shaaban, Chirlei Glienke

**Affiliations:** ^1^Postgraduate Program in Microbiology, Department of Pathology, Federal University of Paraná (UFPR), Centro Politécnico, Curitiba, Paraná, Brazil; ^2^Center for Pharmaceutical Research and Innovation, College of Pharmacy, University of Kentucky, Lexington, KY, United States; ^3^Department of Pharmaceutical Sciences, College of Pharmacy, University of Kentucky, Lexington, KY, United States; ^4^Postgraduate Program in Genetics, Department of Genetics, Federal University of Paraná (UFPR), Centro Politécnico, Curitiba, Paraná, Brazil

**Keywords:** *Diaporthe*, *Xylaria*, natural products, *Vochysia divergens*, Pantanal, Serra do Amolar, secondary metabolites

## Abstract

**Introduction:**

The exploration of new bioactive compounds for agricultural applications is critical for sustainable development. Endophytic fungi, particularly those from underexplored biomes in Brazil, represent a promising source of natural compounds. This study focused on isolation and bioprospecting endophytic fungi from the medicinal plant *Vochysia divergens* (Pohl), grown in Serra do Amolar (Brazilian Pantanal Biome), with an additional emphasis on conserving microbial biodiversity.

**Methods and results:**

Leaves and petioles were collected from 18 V. divergens specimens, from which 293 endophytes were isolated and grouped by morphological characteristics into 91 phenotypes. One representative of each phenotype was selected for secondary metabolite extraction and taxonomic identification. Fungi belonging to 27 families and 32 different genera were identified, with *Diaporthe*, *Phyllosticta*, and *Pseudofusicoccum* as the most predominant. We also introduce and describe a new endophytic species, *Diaporthe amolarensis*. Multiple extracts inhibited mycelial growth of the phytopathogenic fungus *Colletotrichum abscissum*, with a superior effect compared to the fungicide control. These extracts were produced by *Diaporthe amolarensis*, *Xylaria arbuscula*, and *Nemania primolutea*. Additionally, the extract from one *X. arbuscula* isolate displayed moderate activity against the phytopathogen *Phyllosticta citricarpa*. HPLC-UV and HPLC-MS analyses of these most inhibitory extracts revealed natural products with beneficial potential that need characterization and to have their modes of action elucidated.

**Discussion:**

Finally, a very important contribution of this study was the *ex situ* conservation of the biodiversity of the Serra do Amolar, allowing future studies and biotechnological applications involving endophytes from this region.

## 1 Introduction

One of the biggest challenges facing global agriculture is the control of diseases caused by pathogenic microorganisms. Their rapid dissemination capacity and ability to affect crop production have a severe impact on national agriculture, leading to significant economic losses (Fontes and Valadares-Inglis, 2020). Phytosanitary management involves various practices, with the application of chemical pesticides being one of them. Despite the increase in pesticide use over the recent decades, this has not resulted in a proportional reduction in crop losses (Tan et al., 2018; Sang and Kim, 2020). The large-scale use of pesticides and fungicides has led to its own sets of problems, including the resistance of some pathogenic microorganisms (Morandi et al., 2009; Braga et al., 2022; Torres-Rodriguez et al., 2022). Among these harmful pathogens of most concern are fungi from several genera, such as *Colletotrichum* (Lima et al., 2011; Silva et al., 2017), *Phyllosticta* (Baldassari et al., 2006; Tonial et al., 2017), and *Fusarium* (Brown et al., 2012; Paccanaro et al., 2017). Therefore, species of these genera were used as targets for testing new compounds with activity against phytopathogens.

Considering the risk of pathogen resistance to commonly used fungicides, it has become increasingly necessary to control these pathogens and their negative impacts with new chemical classes that have modes of action different from existing fungicides (Javed et al., 2021; Afzal et al., 2023). Biotechnological advances improve biosynthetic processes, including the production of bioactive compounds of diverse natural substances that can significantly support human, animal, and plant health (Faria et al., 2023). Such compounds, derived from plants, animals, and microorganisms, are gaining considerable attention (Rafiq et al., 2024). Our research group has been dedicated to the prospection and discovery of secondary metabolites (SMs) produced by endophytic microorganisms of medicinal plants from underexplored Brazilian biomes (Savi et al., 2015; Gos et al., 2017; Noriler et al., 2018; Savi et al., 2018; Iantas et al., 2021; Glienke et al., 2024). Endophytes are a dependable source of natural products, as they colonize the internal tissues of plants without causing damage to their host, an interaction based on the exchange of signals and compounds (Petrini et al., 1993; Araújo et al., 2014). In this symbiotic relationship, the endophytes receive protection and nutrients and in return produce SMs that protect the host by increasing its resistance to pathogens (Savi D. et al., 2019).

Brazil is a country with enormous biodiversity, comprising six different biomes, some of which are understudied. One of them is the Pantanal (Glienke et al., 2024), the largest continuous wetland in the world, located in the states of Mato Grosso and Mato Grosso do Sul (in addition to Bolivia and Paraguay), experiencing drastic seasonal fluctuations, with alternating periods of floods and droughts. This biome has an enormous richness of species, and its diversity is strongly related to the seasonality of the region, offering the potential to discover an abundance of natural products. The plant *Vochysia divergens* (Cambará) belongs to a group of approximately 5% of the tree species of the Pantanal biome that is capable of surviving in regions long flooded and therefore became dominant in that area. In addition, it has medicinal properties and is used in teas and syrups for the treatment of colds, coughs, pneumonia, and gastrointestinal diseases (Arieira and Da Cunha, 2006).

The Serra do Amolar, an environmental preservation area, managed by the Serra do Amolar Protection and Conservation Network, in the Pantanal biome, is located in the north of the state of Mato Grosso do Sul, on the border with the Mato Grosso state and Bolivia. It is one of the largest biological heritage sites in Brazil. Our research group has been studying endophytes from medicinal plants located in other regions of the Pantanal and found several new endophytic natural products (Savi et al., 2015; Gos et al., 2017; Noriler et al., 2018; Savi et al., 2018; Iantas et al., 2021). Because the Serra do Amolar region is difficult to access and consequently little studied, it is much more native than other areas of the Pantanal (Fundação Ecotrópica, 2003; Moreira, 2011). Our hypothesis is that the plants in this region harbor a greater diversity of endophytes with greater potential to produce undiscovered compounds with beneficial properties. Additionally, this region has been recently suffering from wildfires, placing its biodiversity at risk (Pessi et al., 2023). Therefore, this study aimed to extend our knowledge of endophytic fungi present in the Pantanal Biome through bioprospecting the medicinal plant *Vochysia divergens* from the Serra do Amolar. To evaluate its potential for producing SMs with biological activities, we isolated and identified the endophytic community, extracted SMs, and tested them against three phytopathogens. This study contributes not only to discover new SMs with biotechnological applications but also to explore the diversity of endophytes present in this underexplored region and, in addition, conducts an *ex situ* conservation of the isolates.

## 2 Materials and methods

### 2.1 Sampling of plant material

**Figure 1:**
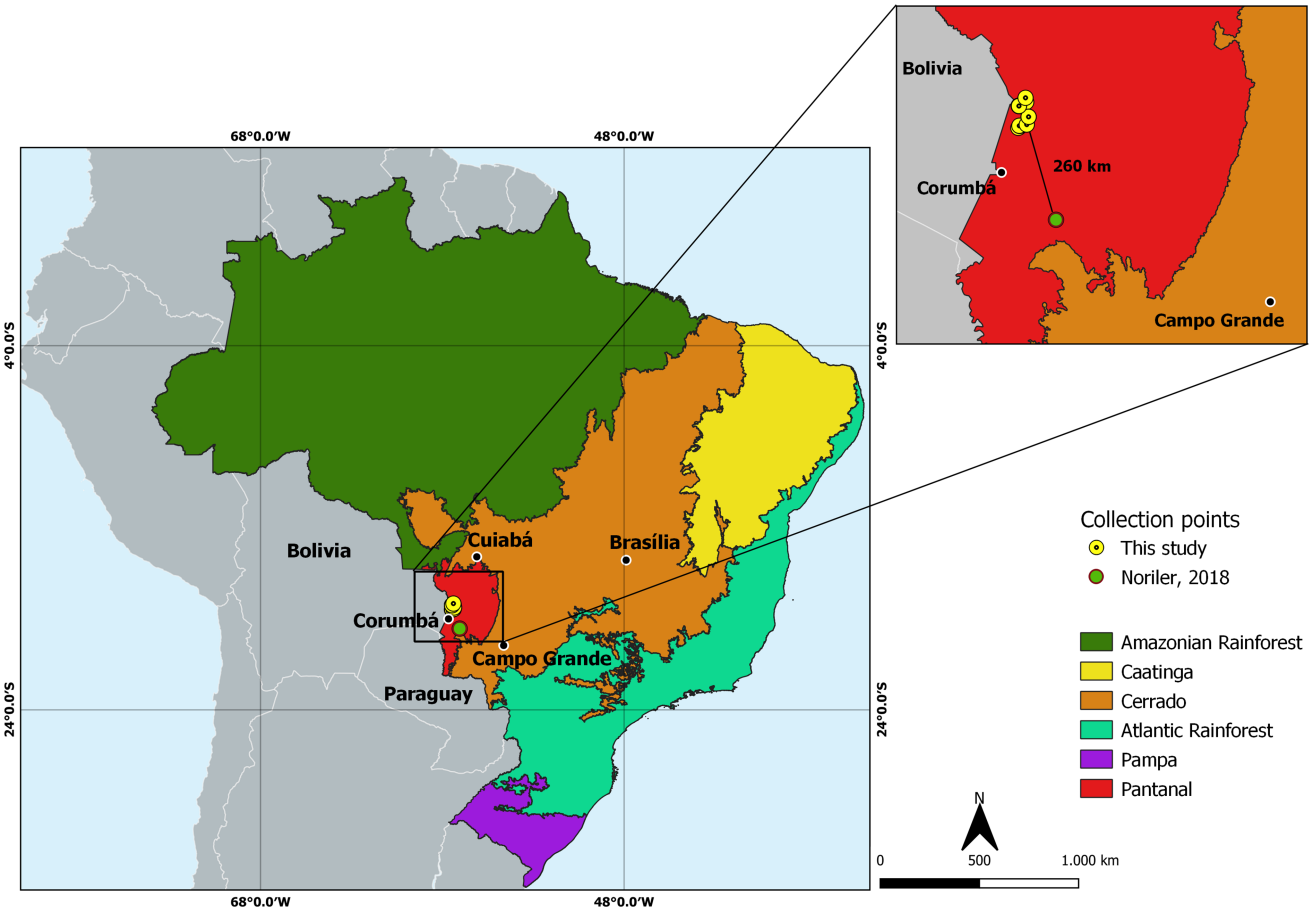
Map of Brazil showing the division by biomes and the sample collection place. The magnified box shows the points of collection of leaves and petioles of *Vochysia divergens* used in this study (yellow dots) in contrast to the point of collection of leaves and petioles of the same plant in the previous study carried out by Noriler et al. (2018) (red dot) with approximately 260 km of distance between them. Source: The authors.

Sampling of plant material occurred in February 2019 with assistance from the Serra do Amolar Institute.^1^ We sampled the leaves and petioles of 18 *V. divergens* trees along a section of the Paraguay River (Figure 1; Supplementary Table S1) within the Serra do Amolar. The collected samples were stored in plastic bags and kept under refrigeration until processing.

### 2.2 Isolation of endophytes

After collection, five leaves and five petioles from each plant without marks or injuries were used to isolate endophytic fungi. To eliminate the epiphytic microorganisms, a surface disinfection protocol was performed as described by Petrini (1986) and adapted by Noriler et al. (2018). Then, the leaves and petioles were fragmented into five pieces (8-mm squares), each of which was placed onto Petri dishes containing potato dextrose agar (PDA), pH 5.8, with the addition of tetracycline (50 μg/mL) to inhibit bacterial growth. The plates were incubated at 28°C for up to 30 days, and fungal growth was checked daily. The emerging mycelia were transferred to fresh PDA plates at pH 5.8 and stored for later use.

The isolates were grouped according to their macromorphological characteristics, such as colony color, hyphal texture, and growth rate. A pure culture of each representative phenotype was generated, using the single spore culture method according to Gilchrist-Saavedra et al. (2006). The pure cultures were used for molecular identification and bioprospecting and were all deposited in the Coleções Microbiológicas da Rede Paranaense (CMRP) culture collection^2^ at the Federal University of Paraná, Brazil. The access to genetic heritage isolated in this study was registered in SISGEN (National System for the Management of Genetic Heritage and Associated Traditional Knowledge) under number A74A5EE in compliance with Brazilian Law 13.123/2015, and the Nagoya Protocol. The collection of biological material used in this study was authorized by the Instituto Chico Mendes de Conservação da Biodiversidade (ICMBio), according to authorization SISBIO (Biodiversity Authorization and Information System) No. 85130.

### 2.3 Identification of endophytes

Genomic DNA was extracted from mycelia following previously published protocols (Raeder and Broda, 1985; Glienke, 1999). Subsequently, partial regions of six loci were amplified according to the specificities of each genus and species. The list of primers used for each amplification is shown in Table 1.

**Table 1:** List of primers used in PCR amplifications.

Region	Primer	Primer DNA sequence	References
ITS	V9G	5’ TTACGTCCCTGCCCTTTGTA 3′	De Hoog and Gerrits van den Ende (1998)
	ITS4	5’ TCCTCCGCTTATTGATATGC 3’	White et al. (1990)
LSU	LROR	5’ GTACCCGCTGAACTTAAGC 3′	Vilgalys and Hester (1990)
	LR5	5’ TCCTGAGGGAAACTTCG 3’	Vilgalys and Hester (1990)
*tub2*	T1	5’ AACATGCGTGAGATTGTAAGT 3′	O’Donnell and Cigelnik (1997)
	T22	5’ TCTGGATGTTGTTGGGAATCC 3′	O’Donnell and Cigelnik (1997)
	Bt2b	5’ ACCCTCAGTGTAGTGACCCTTGGC 3’	Glass and Donaldson (1995)
*tef1*	EF1-728F	5’ CATCGAGAAGTTCGAGAAGG 3′	Carbone and Kohn (1999)
	EF1-986R	5’ TACTTGAAGGAACCCTTACC 3’	Carbone and Kohn (1999)
*his3*	CYLH3F	5’ AGGTCCACTGGTGGCAAG 3′	Crous et al. (2004)
	H3-1b	5’ GCGGGCGAGCTGGATGTCCTT 3′	Glass and Donaldson (1995)
*act*	ACT-512F	5’ ATGTGCAAGGCCGGTTTCGC 3′	Carbone and Kohn (1999)
	ACT-783R	5’ TACGAGTCCTTCTGGCCCAT 3′	Carbone and Kohn (1999)

The PCRs were performed for a final volume of 12.5 μL (1X reaction buffer, 0.2 μM of forward primer, 0.2 μM of reverse primer, 1.5 mM of MgCl_2_, 0.2 mM of dNTPs, and 0.05 U/μL of Taq Polymerase). The PCR conditions used for each gene and taxonomic group are listed in Table 2. The PCR products were purified using the enzymes *Exo1* and *FastAP* (GE Healthcare, USA), and the BigDye® Terminator Kit v3.1 was used for the sequencing reaction. The product of this reaction was purified by gel filtration (Sephadex G50), and the sequencing was determined with an automatic sequencer (ABI3500®, Applied Biosystems, Foster City, CA, USA).

**Table 2:** Conditions used for each amplified fragment.

Region	PCR conditions
ITS (all isolates)	5 min at 94°C, 35 cycles of 30 s at 94°C, 30 s at 48°C and 1 min at 72°C, and a final step of 7 min at 72°C
*act* (Xylariaceae family)	Conditions according to Hsieh et al. (2005)
*tub2* (Xylariaceae family)	Conditions according to Hsieh et al. (2005)
LSU (Xylariaceae family)	Conditions according to Daranagama et al. (2015)
*tef1* (*Diaporthe* genus)	Conditions according to Gomes et al. (2013)
*tub2* (*Diaporthe* genus)	5 min at 94°C, 40 cycles of 30 s at 95°C, 50 s at 58°C and 1 min at 72°C, and a final step of 5 min at 72°C
*his3* (*Diaporthe* genus)	5 min at 95°C, 35 cycles of 30 s at 95°C, 30 s at 55°C and 2 min at 72°C, and a final step of 5 min at 72°C

The obtained chromatograms were inspected using MEGA X (Tamura et al., 2011) and BioEdit (Hall, 1999). The sequences were compared with those available in the NCBI/GenBank database^3^ using the BLAST Tool and compared with type strains obtained from the MycoBank^4^ and Westerdijk Fungal Biodiversity Institute^5^ databases (Supplementary Tables S2–S4, S11–S49). Phylogenetic analyses were performed with the sequences that correspond to the type or authentic strains and those generated by this study. The alignments of the DNA sequences were made using Mafft software (Katoh and Toh, 2008)^6^ and verified manually with MEGA X software. Bayesian inference analysis was inferred using MrBayes v3.2.6 ×86 (Ronquist et al., 2012) via CIPRES Science Gateway (Miller et al., 2011). This analysis was performed using two parallel runs with one cold and three heated chains each, using the number of generations needed to split frequencies ≤0.01 and a sampling frequency set to every 100 generations. The posterior probability values were calculated after discarding the first 25% of the generated trees as burn-in. The resulting trees are plotted in FigTree v.1.4.2.^7^ The substitution modes were selected for each gene using JModelTest (Darriba et al., 2012). All sequences obtained were deposited at GenBank, and the access codes are listed in Supplementary Table S5.

### 2.4 Morphological characterization/taxonomy

The descriptions provided here were based on macromorphological characteristics in different culture media and micromorphological characteristics from sporulated colonies, which formed asexual structures. These observations were conducted for the isolates to which the new species descriptions apply. For the isolate CMRP4997, the following culture conditions were used: PDA pH 5.5, oatmeal agar (OA), and 2% malt extract (MEA) with and without the addition of sterile pine needles and autoclaved leaves of *Schinus terebinthifolius*, incubated at 25°C over a period of 12 h of light and 12 h of darkness as described by Gomes et al. (2013). Colony diameter measurements were determined at 25°C in the dark in PDA, pH 5.5, OA, and MEA media, with measurements performed 3, 4, 5, and 7 days after inoculation. After 15 days, colony colors were described (verse and reverse) using color charts of Ranyer (1970). Alpha and beta conidia were measured to calculate the mean size and standard deviation using ImageJ software. The micromorphological characteristics were examined and captured using a BX51 Olympus microscope equipped with an SC30 camera from the microscopy center (Centro de Tecnologias Avançadas em Microscopia—UFPR).

### 2.5 Extraction and evaluation of endophyte SMs

#### 2.5.1 SM extractions

Endophytic isolates representing each phenotype were selected for small-scale fermentations in liquid medium and extract production. The isolates were cultured for 7 days in PDA at pH 5.8 and 28°C. Three mycelial discs (6 mm) were added into Erlenmeyer flasks (250 mL) containing 100 mL of liquid malt extract medium (Schulz et al., 2002) and incubated under constant agitation (180 rpm) for 10 days at 28°C. The cultures were filtered using Whatman no. 4 filter paper to remove the mycelium, and the remaining broth was mixed with 4% of Amberlite® XAD-16 polymer and kept overnight under constant agitation (180 rpm), followed by centrifugation. The resin was washed three times with distilled water and extracted three times with methanol (MeOH). The solvent was evaporated *in vacuo* at 45°C to obtain the dry crude extract and then diluted in MeOH to a final concentration of 10 mg/mL (Iantas et al., 2021).

#### 2.5.2 SM mycelial growth inhibition of pathogenic Fungi

The phytopathogenic fungus *C. abscissum* CMRP704 was used for the initial screening of the antifungal activity of the crude extracts. The experiment was carried out with 100 μL of each extract (10 mg/mL) added to a Petri dish containing PDA pH 5.8 and spread using a Drigalski spatula, followed by a pathogen mycelial disc (6 mm) placed in the center of the plate. The fungicide Carbendazim (Derosal®; 1.0 mg/mL) and pure methanol were used as positive and negative controls, respectively. The plates were incubated at 24°C for 7 days, and the diameter of the colonies was measured and compared with that of both control plates (Savi, 2011). The percentage of mycelial growth inhibition was calculated with the following formula: Pi = (Cd – Td)/Cd, where Pi = percent inhibition; Cd = control growth diameter; Td = treatment growth diameter. The experiments were carried out in triplicate.

The extracts that presented mycelial growth inhibition higher than the positive control (fungicide Carbendazim) in the previous analysis with *C. abscissum* were also evaluated against the phytopathogens *F. graminearum* LGMF1703 and *P. citricarpa* CMRP06 (Glienke et al., 2011), using the same methodology, with the following modifications: (1) the incubation temperature used for *F. graminearum* was 28°C, (2) the growth inhibition rates were analyzed at 4 and 21 days after inoculation for *F. graminearum* and *P. citricarpa*, respectively. The experiments were performed in triplicate.

#### 2.5.3 Chemical analyses of SMs

To identify the potential compounds in the prioritized bioactive fungal extracts, we conducted chromatography analyses using HPLC-UV/MS. HPLC-UV/MS analyses were accomplished with an Agilent InfinityLab LC/MSD mass spectrometer (MS Model G6125B; Agilent Technologies, Santa Clara, CA, USA) equipped with an Agilent 1260 Infinity II Series Quaternary LC system and a Phenomenex NX-C18 column (250 × 4.6 mm, 5 *μ*m) [method: solvent A: H_2_O/0.1% formic acid, solvent B: CH_3_CN; flow rate: 0.5 mL min^−1^; 0–30 min, 5–100% B (linear gradient); 30–35 min, 100% B; 35–36 min, 100–5% B; 36–40 min, 5% B]. All solvents used were of ACS grade and purchased from Pharmco-AAPER (Brookfield, CT). A549, PC3, and HEL299 cells were obtained from ATCC (Manassas, VA). All other reagents used were reagent grade and purchased from Sigma-Aldrich (Saint Louis, MO).

#### 2.5.4 Cytotoxicity assays for SM extracts

The cytotoxicity of crude extracts was evaluated against A549 (non-small-cell lung carcinoma), PC3 (prostate adenocarcinoma), and HEL299 (normal lung fibroblast) human cell lines. The assays were accomplished in triplicate following our previously reported protocols (Savi et al., 2018; Shaaban et al., 2013, 2015; Wang et al., 2013) actinomycin D (A549, PC3, and HEL299) was used as a positive control.

## 3 Results

### 3.1 Endophyte diversity observed with *Vochysia divergens* from the Serra do Amolar

A total of 293 cultivable endophytic fungi were isolated from leaf and petiole fragments collected from 18 trees of *V. divergens*. The isolates were grouped into 91 phenotypes according to their morphological characteristics (Supplementary Figure S1). A representative isolate of each phenotype was identified at the genus and/or species level using phylogenetic analyses (Figures 2–4; Supplementary Figures S2–S36).

**Figure 2:**
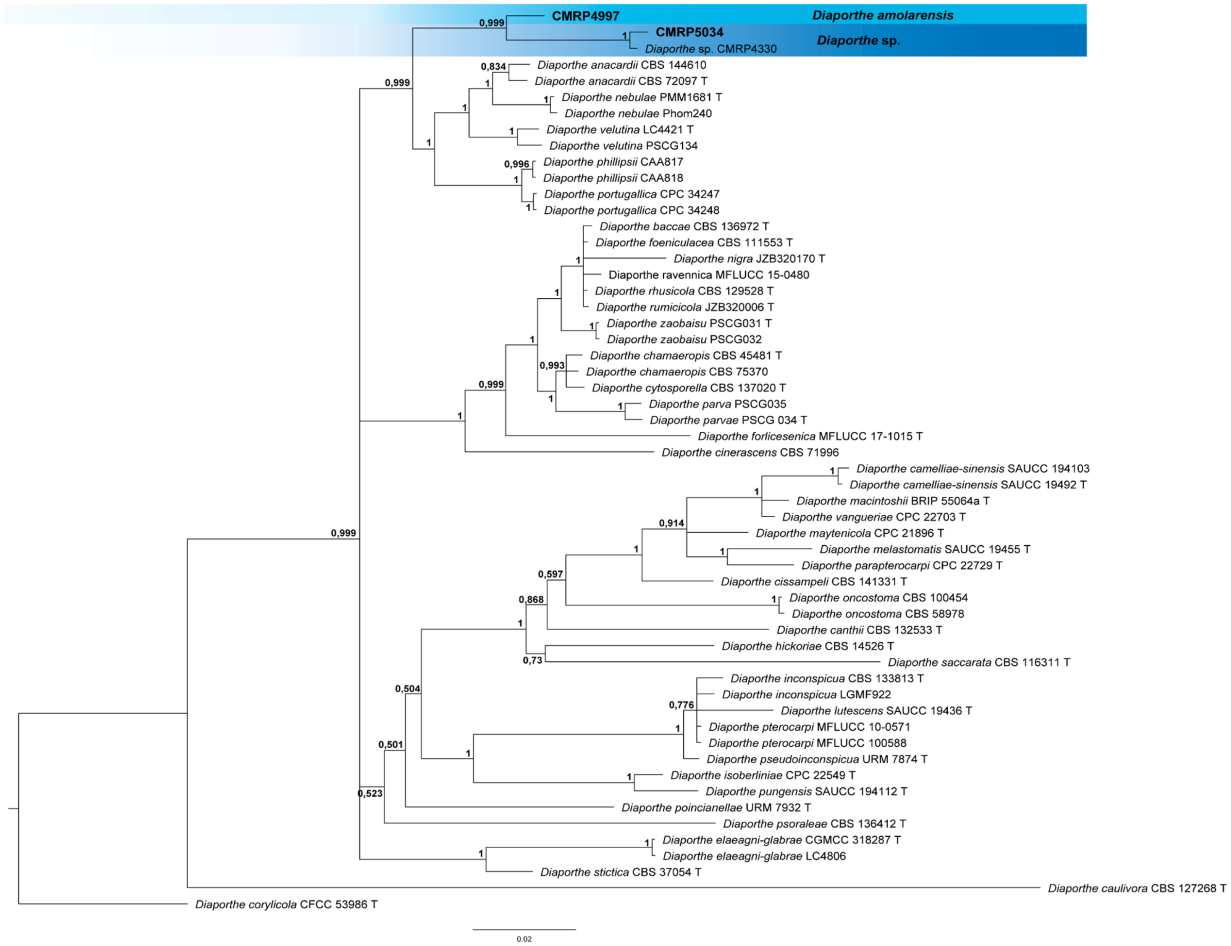
Bayesian inference phylogenetic tree of *Diaporthe oncostoma* species complex based on multiple alignments of *tef1*, *tub2,* and *his3* partial sequences. The data matrix had 56 taxa and 1750 characters. The species *Diaporthe corylicola* (CFCC 53986) was used as an outgroup. Strains marked with a “T” correspond to type sequences. Bayesian posterior probabilities equal to or greater than 0.50 are presented next to each node. The scale bar of 0.02 represents the number of changes. The sequence of the isolates here studied is presented with their isolation codes (CMRP4997 and CMRP5034) highlighted in bold.

**Figure 3:**
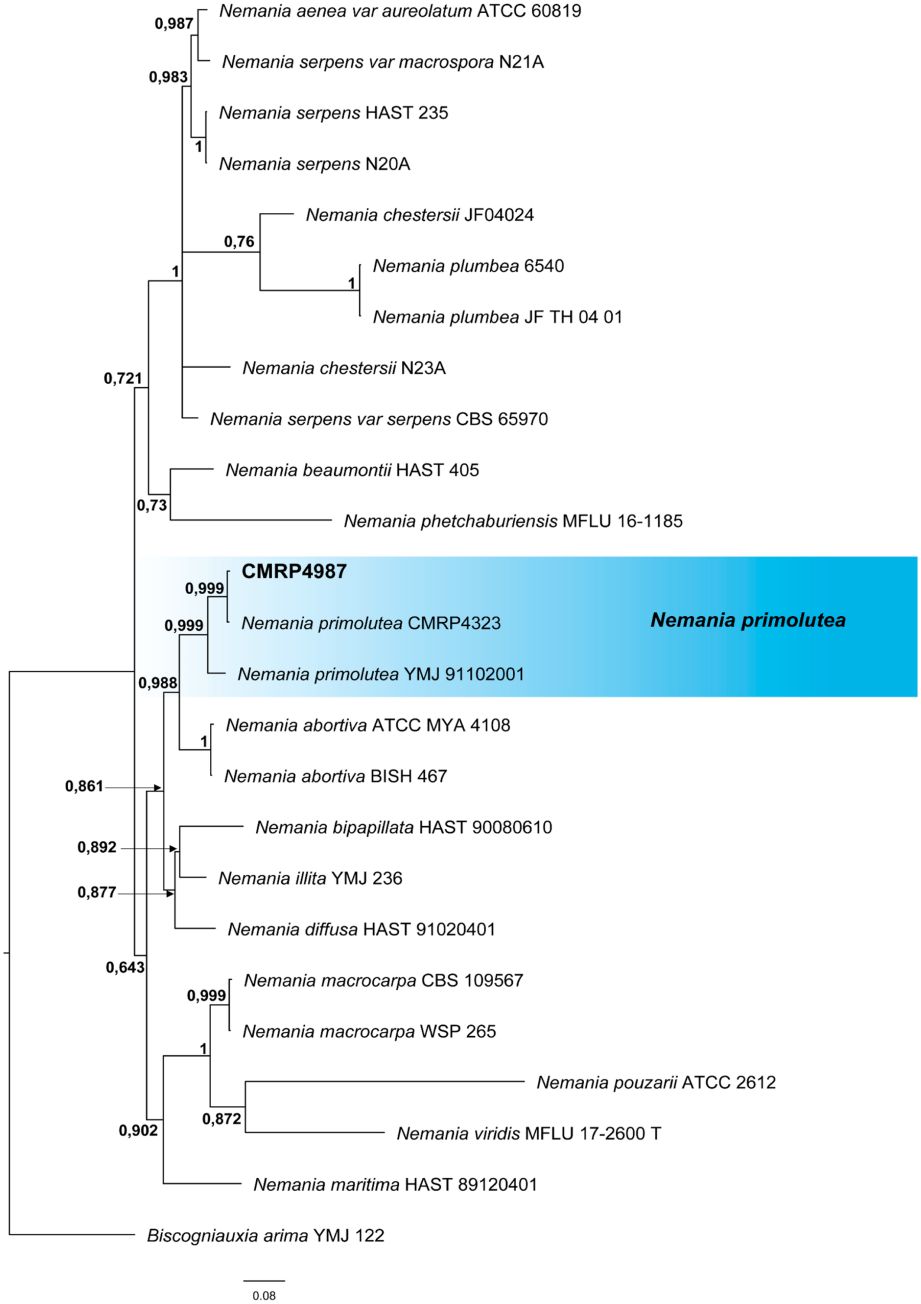
Bayesian inference phylogenetic tree of *Nemania* species based on multiple alignments of ITS, *act*, and *tub2* partial sequences. The data matrix had 25 taxa and 2,846 characters. The species *Biscogniauxia arima* (YMJ 122) was used as an outgroup. Strains marked with a “T” correspond to type sequences. Bayesian posterior probabilities equal to or greater than 0.50 are presented next to each node. The scale bar of 0.08 represents the number of changes. The sequence of the isolate here studied is presented with its isolation code (CMRP4987) highlighted in bold.

**Figure 4:**
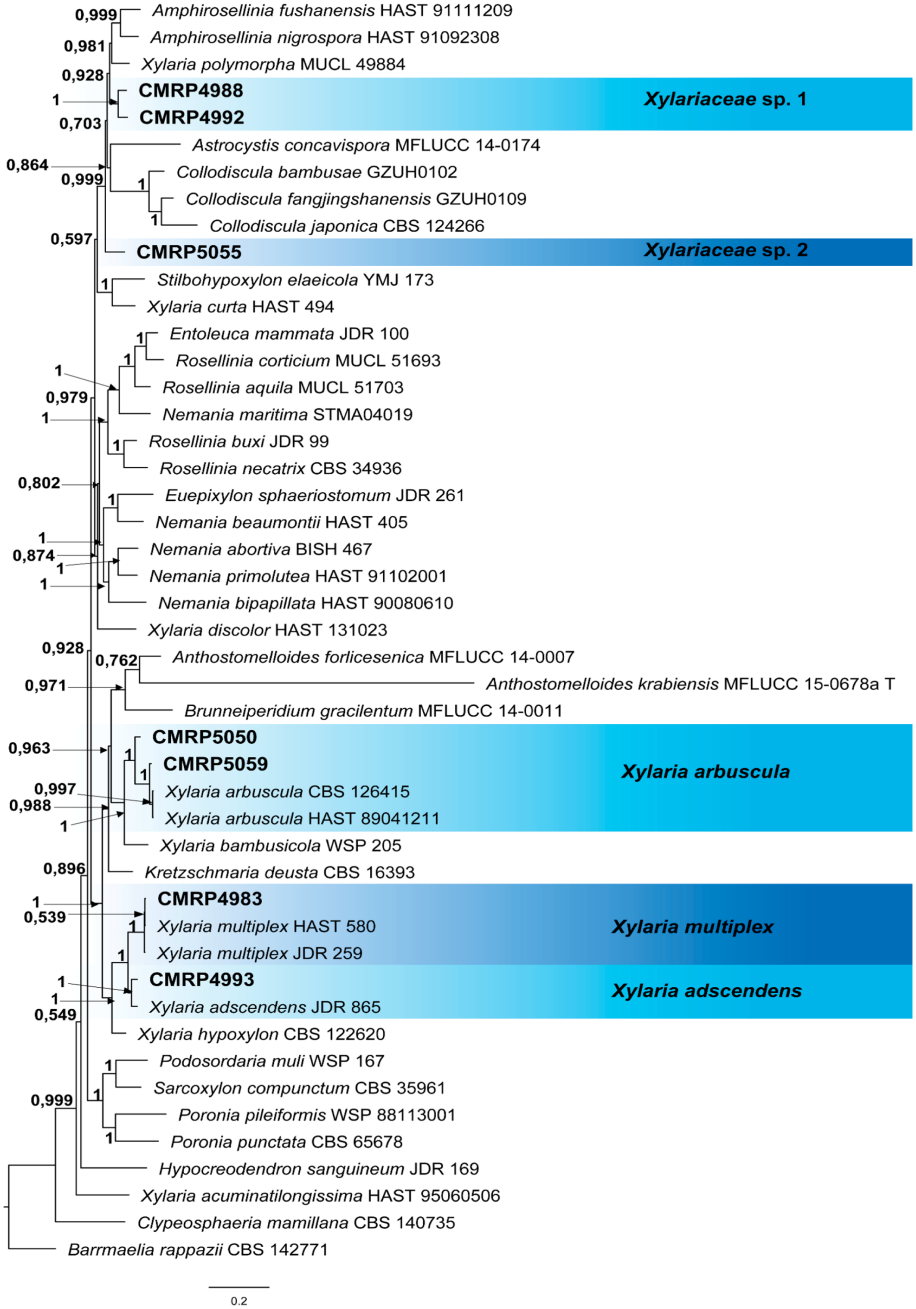
Bayesian Inference phylogenetic tree of species from *Xylariaceae* family based on multiple alignments of ITS, LSU, and *tub2* partial sequences. The data matrix had 47 taxa and 5,647 characters. The species *Barrmaelia rappazii* (CBS 142771) was used as an outgroup. Strains marked with a “T” correspond to type sequences. Bayesian posterior probabilities equal to or greater than 0.50 are presented next to each node. The scale bar of 0.2 represents the number of changes. The sequences of the isolates here studied are presented with their isolation codes (CMRP4988, CMRP4992, CMRP5055, CMRP5050, CMRP5059, CMRP4983, and CMRP4993) highlighted in bold.

**Figure 5:**
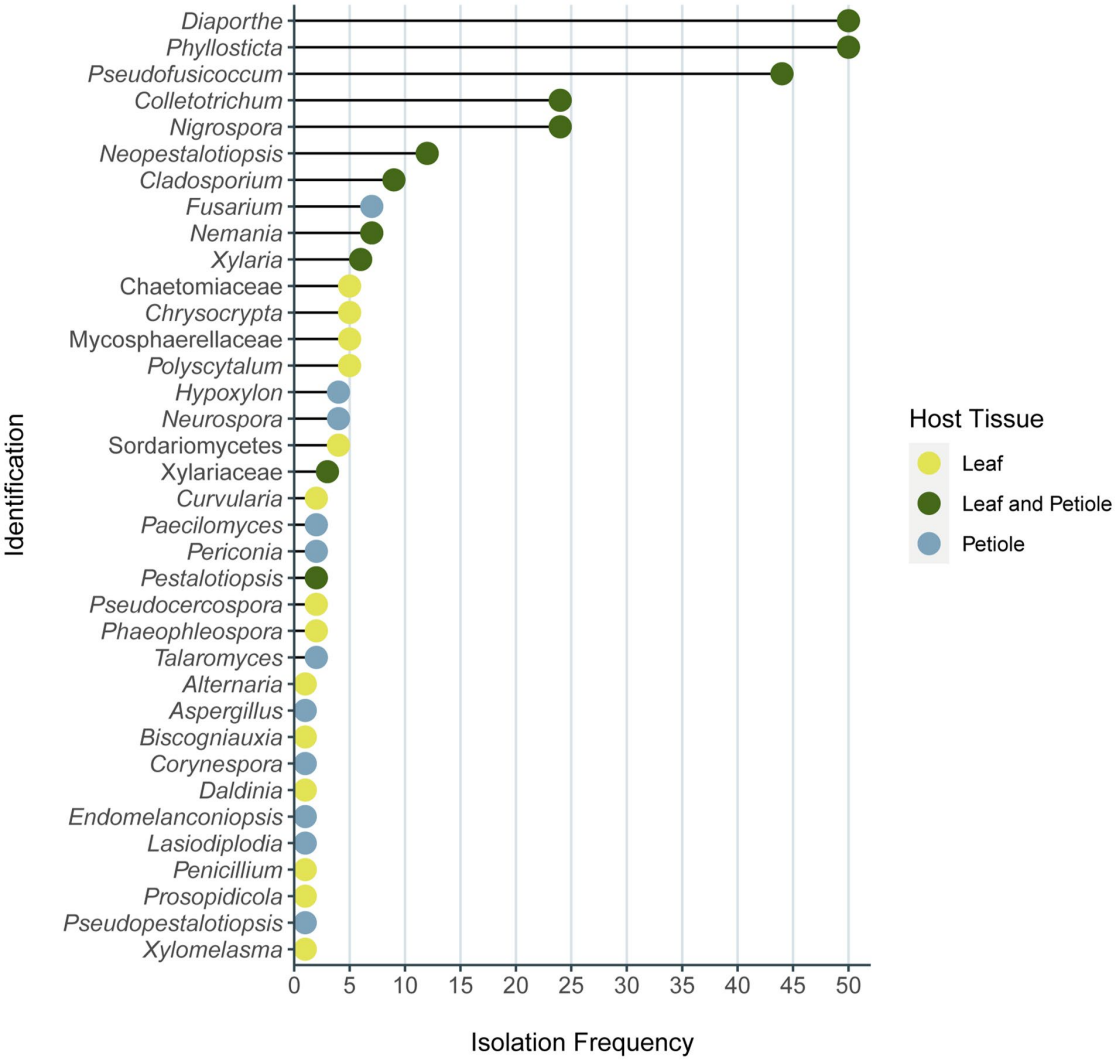
Frequency of endophyte isolates identified of each genus or family. Number of endophytes of each genus and family isolated from leaves, petioles, or both of plant *Vochysia divergens* in the present study. This result considers the molecular identification of each phenotype representative isolates. The color of each dot represents the host tissue origin of each genera/family, the isolates that came only from leaves are indicated in yellow, the ones from petiole only are indicated in blue, and the green dots mean that the isolates came from both plant tissues.

All the identified phenotypes belong to the Phylum Ascomycota within three classes: Eurotiomycetes, Dothideomycetes, and Sordariomycetes. Sordariomycetes was the dominant class corresponding to 69% of the isolates, and the dominant orders in this class were Diaporthales (25%, with 56 isolates belonging to three families and three genera), Glomerellales (21%, with 24 isolates of one family), and Xylariales (21% with 27 isolates of six genera of four families). The 293 isolates belong to 32 different genera of 27 families (Figure 5; Supplementary Table S6). All isolates were included in the same phenotype as the representative isolate used in the identification analysis belong to the same family/genera. *Diaporthe* and *Phyllosticta* were the most frequent genera isolated, with 50 isolates obtained from each of one, followed by *Pseudofusicoccum* with 44 isolates (Figure 5; Supplementary Table S6). It was also observed that among the most isolated genera the individuals were predominantly obtained by both leaf and petiole tissues, while in the least frequent genera, the host tissue specificity was more evident (Figure 5).

Thirteen isolates did not group with any type genera from the families Chaetomiaceae (5), Mycosphaerellaceae (5), and Xylariaceae (3). These are families not yet very well resolved phylogenetically (Supplementary Figures S2, S3; Figure 4). We suggest that additional gene sequences need to be obtained to identify these 13 isolates at a more restricted taxonomic level. Thus, in the present study, these isolates were assigned only at the family level. Other four isolates did not cluster with any family of the Onygenales order, then with an analysis based on a reevaluation study of this order, those isolates are currently located in an *incertae sedis* clade in Onygenales (Kandemir et al., 2022; Supplementary Figure S4). Therefore, those isolates remain identified on an Order level until further resolution studies.

All identifications were performed based on phylogenetic analysis individually for each genus or family and the trees are shown in the Supplementary material (Supplementary Figures S2–S36). The three dominant genera isolated in this study (*Diaporthe*, *Phyllosticta,* and *Pseudofusicoccum*) are discussed below. To identify the four strains producing bioactive SMs at the species level (i.e., *Diaporthe amolarensis* CMRP4997, *Nemania primolutea* CMRP4987, and *Xylaria arbuscula* CMRP5050 and CMRP5059), more robust analyses were performed using multilocus alignments. These analyses are also presented below.

#### 3.1.1 Identification of isolates

##### 3.1.1.1 The genus *Diaporthe*

*Diaporthe* was the dominant genus with 50 isolates obtained as endophytes that were grouped into 13 phenotypes (Supplementary Table S6; Supplementary Figure S1). Based on a phylogeny using the partial sequence of *tef1* with 1,104 pb and 316 taxa (corresponding to the type and representative strains), these 13 phenotypes were identified as belonging to five clades (data not shown). Each clade was submitted to a new analysis based on *Diaporthe* species complex, following a recently reappraisal by Norphanphoun et al. (2022). The results are described below.

###### 3.1.1.1.1 Clade 1 *Diaporthe oncostoma* species complex: *Diaporthe amolarensis* CMRP4997, and *Diaporthe* sp. CMRP5034

Bayesian inference analysis of the *Diaporthe oncostoma* species complex comprising 1750 pb of *tef1*, *tub2*, and *his3* partial sequences showed the strains CMRP4997 and CMRP5034 clustered with CMRP4330 (*Diaporthe* sp.) in a single branch (supported by 0.999 probability), different from the other species present in the clade (Supplementary Tables S2, S7–S10). These three strains are phylogenetically close to the species *D. anacardii*, *D. macadamiae*, *D. nebulae*, *D. velutia*, *D. phillipsii*, and *D. portugallica,* and not distant from the species *D. inconspicua* and *D. pterocarpi* (Figure 2). The CMRP4330 strain was isolated by our group in a previous study (Iantas et al., 2021) also as an endophyte of *V. divergens* from the Pantanal Biome, however, from a different region than the Serra do Amolar. The strain CMRP5034 is very similar to CMRP4330 and was named *Diaporthe* sp. However, the strain CMRP4997 is a separate branch. Due to the long length of the branch, these strains probably do not belong to the same species (Figure 2).

**Figure 6:**
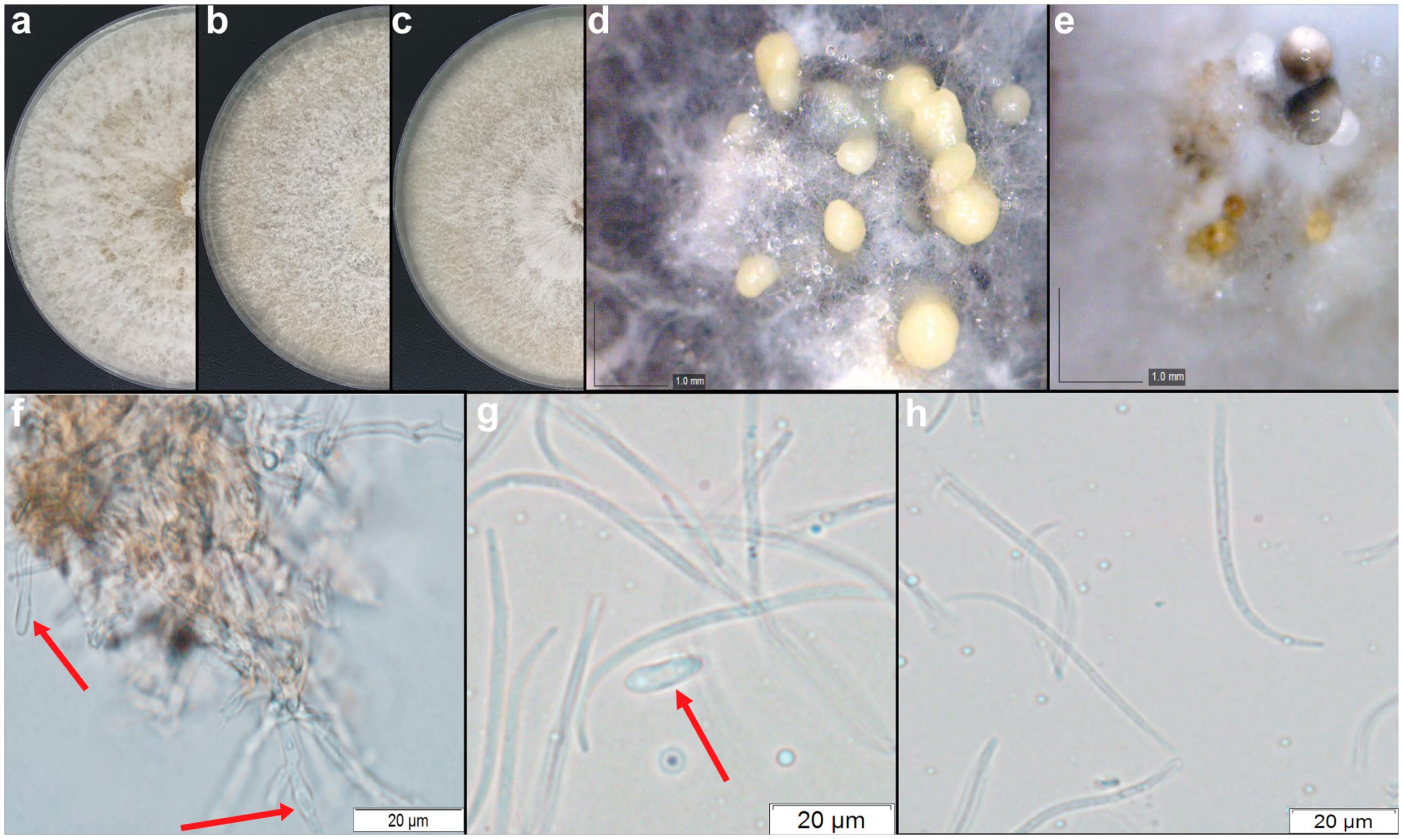
*Diaporthe amolarensis* (CMRP4997). **(A–C)** Colonies at 15 days on PDA, MEA, and OA, respectively. **(D–E)** Conidiomata sporulating on OA and MEA. **(F)** Conidiogenous cells. **(G)** Alpha conidia. **(H)** Beta conidia. Bars: **(F–H)** 20 μm.

The *D. amolarensis* CMRP4997 strain was the only one that showed sporulation in culture, and therefore, it was possible to describe this new species, which was designated *Diaporthe amolarensis*, named after its isolation region, the Serra do Amolar (Pantanal, Brazil). The macro and micromorphology were described using different culture media (Figure 6). Below, we describe this new species.

Species description.

*Diaporthe amolarensis* CMRP4997.

*Diaporthe amolarensis*: Mayrhofer & Glienke, sp. nov. Mycobank 843837 (Figure 6).

*Etymology:* Named after the Pantanal region where it was collected, Serra do Amolar.

Sporulation on PDA and OA culture media. *Conidiomata* pycnidial globose, conical or irregular, solitary or aggregated, exposed on PDA medium surface, dark brown to black, cream translucent conidial drops exuded from the ostioles, 170–350 μm diameter. *Conidiogenous cells* hyaline and subcylindrical, tapering toward the apex 12.2–15.1 × 2–3.5 μm. *Alpha* conidia common, hyaline, fusiform, biguttulate, 12.8–16.1 × 3.3–4.9 μm, mean ± SD = 14.5 ± 1.8 × 4.29 ± 0.44 (*n* = 50). *Beta* conidia spindle-shaped, aseptate, smooth, hyaline, mostly curved toward one end 26.4–35.1 × 0.3–1.5 μm, mean ± SD = 30.26 ± 2.50 × 0.89 ± 0.29 (*n* = 50), and *gamma* conidia absent (Figure 6).

*Culture characteristics*: Colonies covering a dish after 15 days in the dark at 25°C. Colonies on flat PDA, aerial mycelium with cotton texture, white to pale yellow on the surface, colonies reaching 79 mm in diameter after 7 days at 25°C; reverse brown. In flat OA, aerial mycelium with fluffy texture in the center, white on the surface, colonies reaching 79 mm in diameter; reverse light yellow. In the flat MEA, aerial mycelium with cotton texture, white to pale yellow on the surface, colonies reaching 73 mm diameter; reverse yellow to brown forming concentric rings (Figure 6).

*Specimen examined:* Brazil, Serra do Amolar, Pantanal, Mato Grosso do Sul (18°15′37.8”S 57°27′37.4”W), endophytic species isolated from petiole of *Vochysia divergens* (popular name Cambará), February 2019, C. Glienke. Holotype: UPCB 98095 (Herbarium of the Department of Botany code, Federal University of Paraná), ex-type culture CMRP4997 (Microbiological Collections of Paraná Network at Federal University of Paraná).

*Notes*—Endophytic isolate of a medicinal plant in Brazil.

###### 3.1.1.1.2 Clade 2 *Diaporthe sojae* species complex part 1: *Diaporthe vochysiae* (CMRP4977, CMRP4978, CMRP4994, CMRP4996, CMRP5036, and CMRP5220) and *Diaporthe infertilis* (CMRP5061)

Based on phylogenetic analysis of the *Diaporthe sojae partial* species complex using multiple alignments of the partial sequences of ITS, *tef1*, *tub2,* and *his3* (comprising 2,273 characters), these six strains clustered with the type strain of *D. vochysiae* LGMF1583 (Supplementary Figure S5; Supplementary Table S14). This type strain was isolated by our group as an endophyte of *V. divergens* from the Pantanal Biome, however, from a different region than the Serra do Amolar (Noriler et al., 2019).

Moreover, based on the same phylogenetic tree, one strain CMRP5061 was clustered with the type strain (CBS 230.52) and two other representative strains (CBS 19939 and CPC 203.22) of the species *Diaporthe infertilis* (Supplementary Figure S5).

###### 3.1.1.1.3 Clade 3 *Diaporthe sojae* species complex part 2: *Diaporthe cerradensis* (CMRP4985)

Based on the phylogenetic analysis of the *Diaporthe sojae* partial species complex based on the multiple alignment of ITS and *tef1* partial sequences the strain CMRP4985 belongs to *D. cerradensis* species (Supplementary Figure S6; Supplementary Table S15).

###### 3.1.1.1.4 Clade 4 *Diaporthe rudis* species complex: *Diaporthe cf. heveae* 1 (CMRP4989, CMRP5038)

Bayesian Inference analysis of the *Diaporthe rudis* species complex based on the alignment of *tef1* partial sequence revealed strains CMRP4989 and CMRP5038 clustered with CBS 852.97 *Diaporthe cf. heveae* 1 (Supplementary Figure S7; Supplementary Table S16). This species still needs to be better resolved, as there is also *Diaporthe cf. heveae* 2, which belongs to a completely different clade (Gomes et al., 2013).

###### 3.1.1.1.5 Clade 5 *Diaporthe arecae* species complex: *Diaporthe podocarpi-macrophylli* (CMRP4990)

Bayesian Inference analysis of the *Diaporthe arecae* species complex based on the alignment of *tef1* partial sequence showed the strain CMRP4990 clustered with the CGMCC 3.18281 *D. podocarpi-macrophylli* type strain and another representative strain (LC6229) from the same species (Supplementary Figure S8; Supplementary Table S17).

##### 3.1.1.2 The genus *Phyllosticta* (CMRP4971, CMRP4972, CMRP5048)

*Phyllosticta* together with *Diaporthe* was also the dominant genus with 50 isolates obtained as endophytes, but they were grouped into only three phenotypes. Based on a phylogenetic analysis using ITS and the partial sequences of *tef1, act,* and *gapdh* with 2038 characters and 81 taxa (corresponding to the type and representative strains), these three phenotypes were identified as belonging to the species *P. capitalensis* (Supplementary Figure S9; Supplementary Table S18). In the same clade are the strains CMRP4583 and CMRP4660 isolated by Iantas et al. (2021) as endophytes of *V. divergens* and previously identified as *P. capitalensis*. Despite the low support of this branch (0.66 posterior probability), the identity was confirmed based on the nucleotide differences between *P. capitalensis* and *P. paracapitalensis* according to Guarnaccia et al. (2017).

*Phyllosticta* species usually have low morphological diversity, and as phenotypes were assigned based on morphology, we cannot be sure that all 50 *Phyllosticta* isolates belong to the *P. capitalensis* species, although we sequenced the three most morphologically different strains (Supplementary Figure S1).

##### 3.1.1.3 The genus *Pseudofusicoccum* (CMRP4982, CMRP4976, CMRP5047, and CMRP5219)

*Pseudofusicoccum* was one of the most abundant genera, with 44 isolates obtained as endophytes in this study. Based on morphology, we selected four strains for sequencing, and despite representing different phenotypes (Supplementary Figure S1), all were identified as *P. stromaticum* (Supplementary Figure S10; Supplementary Table S19). These isolates clustered with *P. stromaticum* CMRP4328 and *P. stromaticum* LGMF1608, both isolated by our group (Iantas et al., 2021; Noriler et al., 2018) as endophytes from *Stryphnodendron adstringens* and *Vochysia divergens*. Furthermore, an isolate previously identified as *Pseudofusicoccum* sp. LGMF1611 (Noriler et al., 2018) is also located in the same branch with high support, being currently identified as *Pseudofusicoccum stromaticum*.

##### 3.1.1.4 *Nemania primolutea* CMRP4987

Due to the high bioactivities detected in the extract produced by this strain, the isolate of the genus *Nemania* was identified at the species level by multilocus analysis that comprised 2,868 pb of the partial sequences of ITS, *act* and *tub2* of all type and representative strains with GenBank available sequences. The Bayesian Inference analysis (Figure 3; Supplementary Table S3) showed the strain CMRP4987 in the same branch (0.999 posterior probability) as the species *N. primolutea* (YMJ 91102001), sharing 96% similarity (identities = 540/565) and the strain CMRP4328 previously identified as *N. primolutea* with 99% similarity (identities = 586/589). Therefore, the CMRP4987 isolate was identified as *N. primolutea*.

##### 3.1.1.5 The family *Xylariaceae*

Despite the low number of isolates obtained as endophytes in our study (6) belonging to the family *Xylariaceae*, we performed a more robust phylogenetic analysis due to the activity observed by secondary metabolites of the strains CMRP5050 and CMRP5059. Due to uncertainties regarding the taxonomic resolution within *Xylariaceae sensu stricto*, we decided to identify the isolates of this family according to Voglmayr et al. (2018). A multilocus analysis was carried out, comprising 47 taxa and 5,647 characters of the partial sequences of ITS, LSU, *rpb2*, and *tub3* of the strains used in the Voglmayr et al. (2018) study (Supplementary Table S4). The Bayesian inference analysis (Figure 4) showed that our endophyte isolates belong to the Xylariaceae *sensu stricto* family (Voglmayr et al., 2018), which also corresponds to Xylariaceae clade xy1 (Samarakoon et al., 2022).

Two isolates were identified as *Xylaria multiplex* (CMRP4983) and *Xylaria adscendens* (CMRP4993) in the Xylariaceae *sensu stricto*. Three isolates (CMRP4988, CMRP4992, and CMRP5055) remain identified only at the family level because they did not cluster with any genus present in the analysis (Figure 4).

###### 3.1.1.5.1 *Xylaria arbuscula* CMRP5050 and CMRP5059

The isolates CMRP5050 and CMRP5059 were selected for further analysis, due to the biological activity of their secondary metabolites. These two isolates are in the same branch (1 posterior probability) as the species *X. arbuscula* (CBS 126415 and HAST 89041211) (Figure 4). The strain CMRP5050 shares 95% similarity (identities = 511/538) and 95% similarity (Identities = 533/564) with CBS 126415 and HAST 89041211, respectively, and CMRP5059 shares 99% similarity (identities = 530/537) and 98% similarity (identities = 554/563) with CBS 126415 and HAST 89041211, respectively.

### 3.2 Endophytic fungi from Serra do Amolar produce SMs bioactive against phytopathogenic fungi

**Figure 7:**
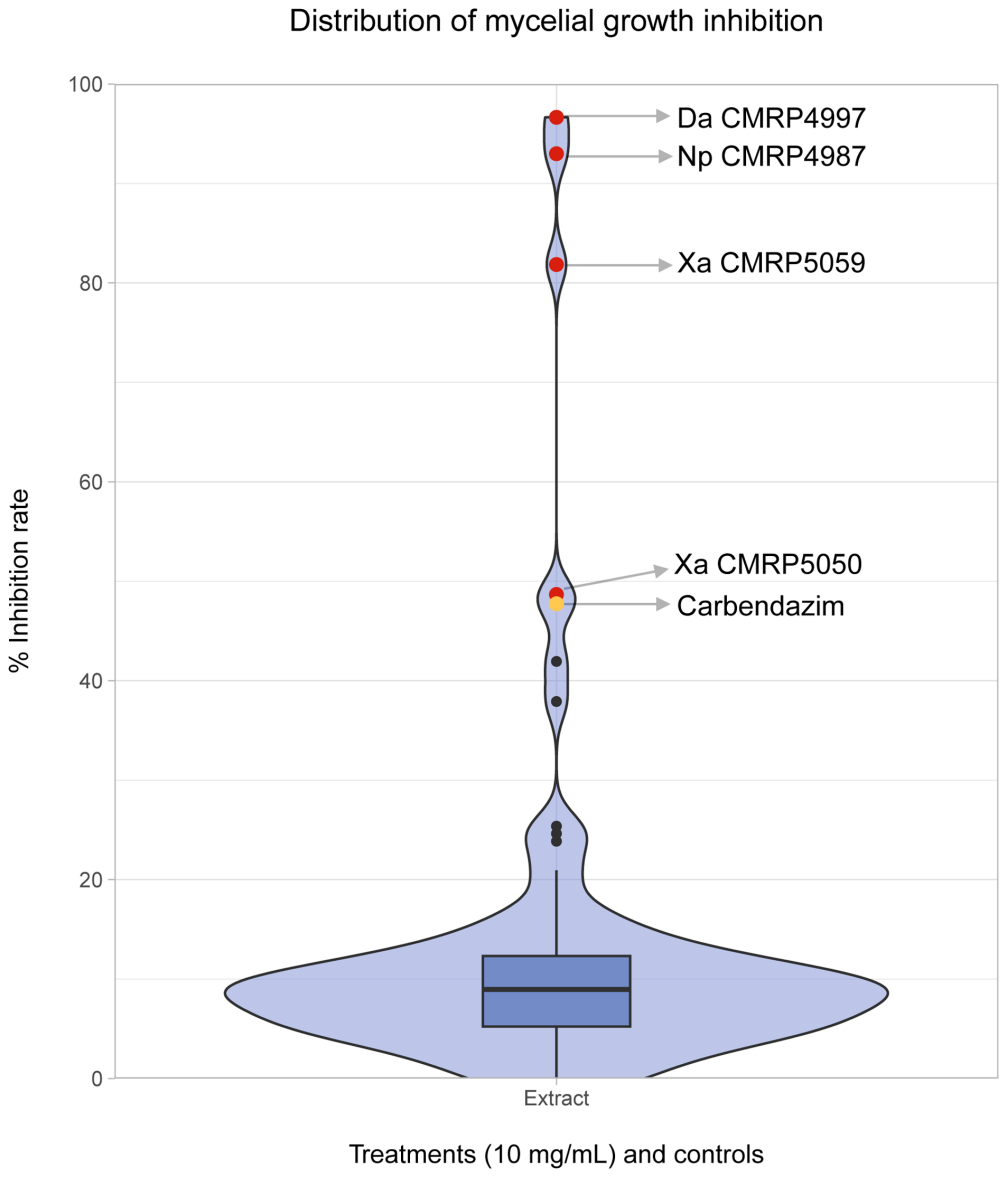
Distribution of mycelial growth inhibition of phytopathogen *Colletotrichum abscissum* (CMRP704) After exposure to extracts produced by endophytes. Distribution of mycelial growth inhibition (in %) of the phytopathogen *Colletotrichum abscissum* in the presence of 100 μL of the extracts at 10 mg/mL obtained by the cultivation of 91 endophytic fungi. The outside points colored in red represent the extracts produced by the isolates *D. amolarensis* (Da) CMRP4997, *N. primolutea* (Np) CMRP4987, *X. arbuscula* (Xa) CMRP5059, and *X. arbuscula* (Xa) CMRP5050, which had higher inhibition activity than the Carbendazim (showed in yellow).

In the antifungal activity screening test of the 91 extracts produced by the endophytes, most treatments exhibited low inhibition of *C. abscissum*, approximately 10%. However, a few extracts stood out, appearing as outliers in the density plot (Figure 7). Among the extracts with higher activity, we highlight four (red points) that demonstrated equal or greater growth inhibition than the commercial fungicide Carbendazim (yellow point): *Diaporthe amolarensis* CMRP4997 (MGI: 96.65%), *N. primolutea* CMRP4987 (MGI: 92.99%), *X. arbuscula* CMRP5059 (MGI: 81.85%), and *X. arbuscula* CMRP5050 (MGI: 48.67%) (Figure 7).

**Figure 8:**
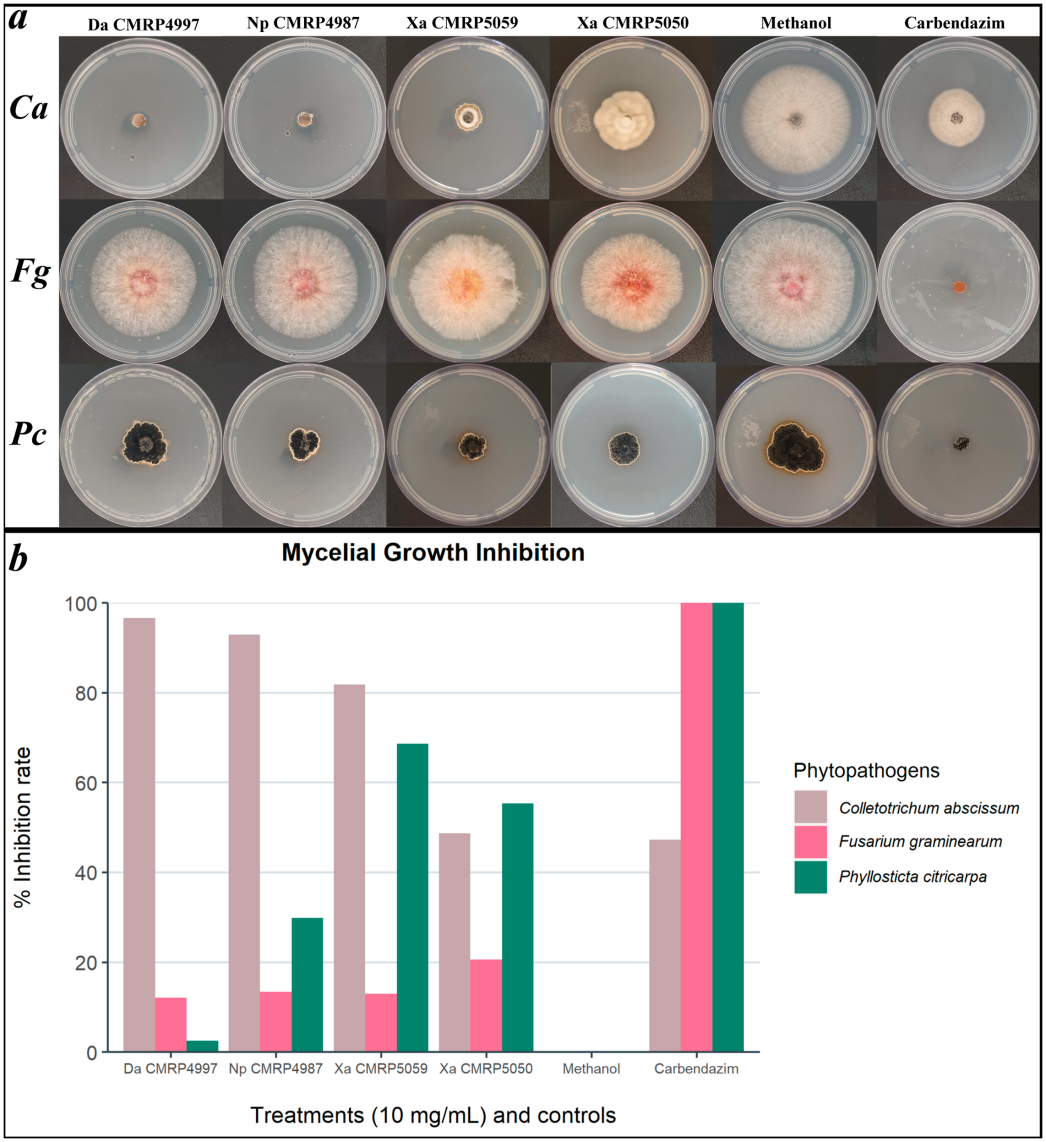
Mycelial growth inhibition of phytopathogens *Colletotrichum abscissum* (CMRP704), *Fusarium graminearum* (LGMF1703), and *Phyllosticta citricarpa* (CMRP06) after exposure to extracts selected in screening test. Mycelial growth inhibition test plates **(A)** and mean of mycelial growth inhibition (in %) **(B)** of the phytopathogens *C. abscissum* (Ca)*, F. graminearum* (Fg), and *P. citricarpa* (Pc) in presence of 100 μL of the extracts from strains *Diaporthe amolarensis* (Da) CMRP4997 (MGI: 12.16%), *Nemania primolutea* (Np) CMRP4987 (MGI: 13.40%), *Xylaria arbuscula* (Xa) CMRP5059 (MGI: 12.96%), and *Xylaria arbuscula* (Xa) CMRP5050 (MGI: 20.64%), compared to the controls (Methanol and Carbendazim).

Based on these results, four extracts were further evaluated for their antifungal activity against the phytopathogenic fungi *F. graminearum* and *P. citricarpa* (Figure 8). Against *P. citricarpa*, the extract produced by strain CMRP5059 *X. arbuscula* stood out, showing the highest mycelial growth inhibition (MGI: 68.64%). Furthermore, the extracts produced by the strains CMRP5050 (*X. arbuscula*) and CMRP4987 (*N. primolutea*) exhibited moderate activity against *P. citricarpa*, with MGI values of 55.38% and MGI: 49.91%, respectively (Figure 8). The extract from CMRP5050 (*X. arbuscula*) also demonstrated the highest inhibition rate against *F. graminearum* (MGI: 20.64%) (Figure 8). As the initial screening test was performed only against *C. abscissum*, we cannot infer about the antifungal activity of the non-evaluated extracts against *P. citricarpa* and *F. graminearum*. Therefore, it is possible that we could observe more active extracts against those two pathogens.

### 3.3 Cytotoxicity

The cytotoxicity of the crude extracts produced by the most interesting four endophytic fungi strains, *Diaporthe amolarensis* CMRP4997, *Nemania primolutea* CMRP4987, *Xylaria arbuscula* CMRP5059, and *Xylaria arbuscula* CMRP5050, were evaluated using A549 (non-small cell lung), PC3 (prostate), and HEL299 (human lung fibroblast). The extracts CMRP4997 and CMRP5059 displayed the highest cytotoxicity assays against all tested cell lines (with less than 10% cell viability). CMRP5050 demonstrated also high cytotoxicity in an assay against all three tested cell lines (~25% cell viability), and CMRP4987 showed low cytotoxicity against A549 and PC3, respectively, but was highly toxic against HEL299 (Supplementary Figure S41).

### 3.4 HPLC-UV/MS analysis of selected fungal extracts

The bioactive extracts produced by the most interesting four endophytic fungi strains, *Diaporthe amolarensis* CMRP4997, *Nemania primolutea* CMRP4987, *Xylaria arbuscula* CMRP5059, and *Xylaria arbuscula* CMRP5050, have been subjected for chemical screening analysis including HPLC-UV and HPLC-MS analyses. The extracts generated from the small-scale fermentations of these fungal strains have been dissolved in MeOH and subjected to HPLC-UV/MS analysis and compared with our microbial natural products database (AntiBase) (Laatsch, 2017) for compound dereplication/identification. The HPLC-UV/MS analysis of the obtained extracts from these four fungal strains displayed several interesting UV/vis and MS peaks (Supplementary Figures S42–S66). The HPLC-UV/MS analysis of the extract produced by the fungal strain, *Diaporthe amolarensis* CMRP4997, displayed five major peaks [HPLC *R*_t_ = 12.91, 27.3, 30.62, 31.25, and 32.45 min] with various UV/vis and molecular weights ranges (MW = 196–414 Daltons) (Supplementary Figures S42–S46). No mass and UV/vis match were found in the AntiBase search, which indicates the potential of this strain to produce new natural products. In the same manner, the HPLC-UV/MS analysis of the extract produced by the endophytic, *Nemania primolutea* CMRP4987, displayed several interesting peaks [HPLC *R*_t_ = 16.04 (MW 431), 21.83 (MW 478) 22.53 (MW 325), 27.19 (MW 332), 29.29 (MW 666), 30.62 (MW 290) and 31.26 min (no clear mass detected), 32.46 min (no clear mass detected)] with various UV/vis and molecular weights ranges (Supplementary Figures S47–S52). The two major LC-UV/MS peaks detected at 31.26 and 32.46 min in the fungal extract of *Nemania primolutea* CMRP4987 were the same as those peaks (31.26 and 32.46 min) detected in the extract of *Diaporthe amolarensis* CMRP4997. AntiBase search for the LC-UV/MS peaks detected from this strain extract indicated no hits and suggests the potential of the endophytic fungus, *Nemania primolutea* CMRP4987, to produce new natural products.

**Figure 9:**
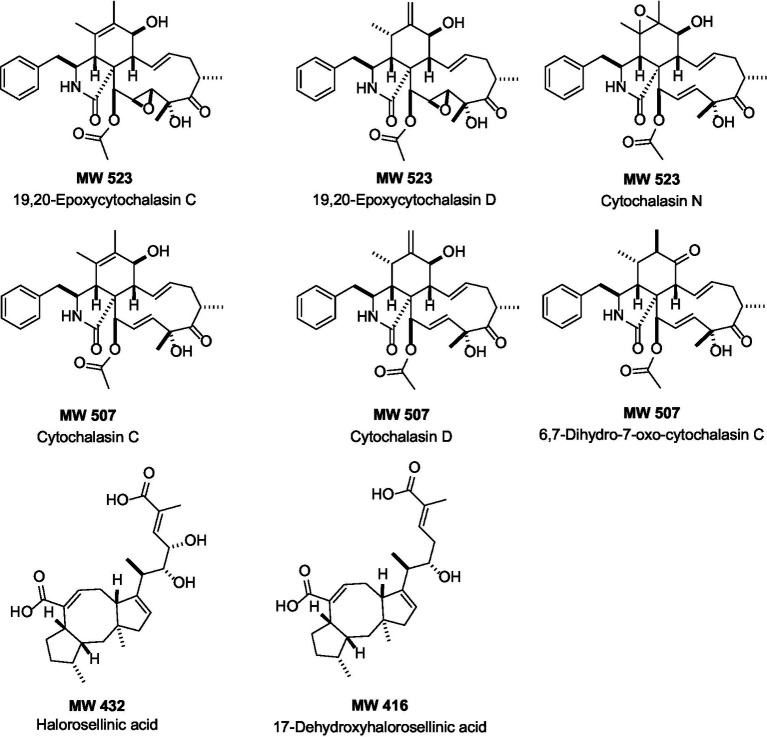
Chemical structures of the compounds identified from the selected endophytic fungal extracts (determined through HPLC-UV/vis and HPLC-ESI-MS profiling, and AntiBase search). MW, Molecular weight.

The HPLC-UV/MS metabolic profiles of the extracts produced by the two fungal strains, *Xylaria arbuscula* CMRP5059 and *Xylaria arbuscula* CMRP5050, indicate interesting compounds with diversity in their chemical structures (various UV/vis chromophores, with over 25 different major metabolites) (Supplementary Figures S53–S66). AntiBase search using the detected molecular weights and UV/vis resulted in the presence of several cytochalasin-analogs as well as halorosellinic acid analogs that match with MS and UV/vis, including 19,20-epoxycytochalasin C (MW 523), 19,20-epoxycytochalasin D (MW 523), cytochalasin N (MW 523), cytochalasin C (MW 507), cytochalasin D (MW 507), 6,7-dihydro-7-oxo-cytochalasin C (MW 507), halorosellinic acid (MW 432), and 17-dehydroxyhalorosellinic acid (MW 416), all of them have been reported previously from fungi (Edwards et al., 1989; Fujii et al., 2000; Chinworrungsee et al., 2001; Chinworrungsee et al., 2002; Chen et al., 2011; Wang et al., 2019; Figure 9). All the remaining LC-UV/MS peaks identified from these two fungal extracts did not match any UV/vis and mass data in AntiBase, which indicates the potential of these two fungal strains to produce interesting bioactive new natural products including new cytochalasins- and halorosellinic acid analogs. Based on the aforementioned LC-UV/MS and bioactivity data results, this study highlights that some of these selected strains including *Xylaria arbuscula* CMRP5059 and *Xylaria arbuscula* CMRP5050 are considered promising strains to produce new bioactive natural products, and have been selected for future studies (including scale-up fermentation, isolation, structure elucidation/compound identification, and bioactivities of the new bioactive compounds produced by these endophytic fungi) and will be published later.

## 4 Discussion

Considering the importance of discovering new bioactive compounds for agriculture applications, the potential of endophytes to produce novel natural products from an underexplored biome in Brazil combined with the need for microbial biodiversity conservation, we conducted a bioprospecting study on endophytic fungi of the medicinal plant *V. divergens,* from the Serra do Amolar to produce SMs with biological activity against phytopathogens. We also acted on the *ex situ* conservation of the endophytes, preserving them in the CMRP Culture Collection of the TaxOnline Network.

The isolation data presented in this study indicate a high diversity of endophytic fungi associated with the medicinal plant *V. divergens* from the Serra do Amolar region of the Pantanal wetlands. Phylogenetic analysis revealed that we isolated at least 54 different species from 27 families and 32 genera and at least 19 new species of endophytic fungi. We consider this richness to be very high, given that we used only 18 plant specimens of the same species collected from the same region in the Pantanal Biome. The extensive fire that occurred in 2020 affected more than 90% of this environmental conservation region, with an estimated loss of 742,000 trees and approximately 17 million vertebrate animals, mainly reptiles (Pletsch et al., 2021). Some of the new species reported in this study, which were isolated in 2019, may no longer be present *in situ*, however, were preserved *ex-situ* at the CMRP culture collection. This underscores the importance of isolating and preserving endophytes in culture collections, where they will be stored and available for future research (Smith and Ryan, 2008).

These results corroborate the findings of previous studies that also isolated endophytes from the plant *V. divergens* (Noriler et al., 2018; Iantas et al., 2021). Noriler et al. (2018) isolated 777 endophytic fungi from leaves and petioles of the plant *V. divergens,* also collected in the Pantanal, but in a different region, approximately 260 km from the sample collection area of this study, near the Miranda River. The isolates belonged primarily to the Phylum Ascomycota, although some were Basidiomycota. The class Sordariomycetes was dominant, similar to what was observed in the present study, with Diaporthales being the most dominant order among main orders. In addition, as in the present study, *Diaporthe* was the most dominant genus. The genus *Diaporthe* belongs to the family Diaporthaceae and comprises hundreds of species (Chepkirui and Stadler, 2017), being recently considered a paraphyletic genus (Gao et al., 2017). This genus is distributed worldwide and its species are able to colonize a wide range of hosts in various associations, occurring as endophytes, plant pathogens, and saprobes (Udayanga et al., 2012; Gomes et al., 2013). Among endophytic fungi, the genus *Diaporthe* is one of the most commonly isolated from various host plants and is frequently associated with the production of secondary metabolites with diverse biological activities (Chepkirui and Stadler, 2017; Gomes et al., 2013). Most of the bioactive SMs recently described from isolates of this genus were obtained from endophytes associated with medicinal plants (Chepkirui and Stadler, 2017), with some recent studies demonstrating antifungal activity against citrus phytopathogens such as *C. abscissum* and *P. citricarpa* (Tonial et al., 2017; Noriler et al., 2018; Savi et al., 2020; Iantas et al., 2021). Investigations of this study support the findings of high antifungal activity obtained by *Diaporthe amolarensis*, which was also reported as a new species.

Over the years, due to the need to explore compounds from natural sources, our group has conducted numerous bioprospecting studies, reporting the production of SMs of endophytic microorganisms (Savi et al., 2015; Gos et al., 2017; Noriler et al., 2018; Savi D. C. et al., 2019). In this study, we report four extracts produced by endophytic fungi that inhibited the mycelial growth of the phytopathogen *C. abscissum*: *Diaporthe amolarensis* CMRP4997 (MGI: 96.65%), *N. primolutea* CMRP4987 (MGI: 92.99%), *Xylaria arbuscula* CMRP5059 (MGI: 81.85%), and *X. arbuscula* CMRP5050 (MGI: 48.67%). The first three extracts inhibited the mycelial growth of the phytopathogen levels higher than the positive control, the fungicide Carbendazim. *C. abscissum* is the causal agent of postbloom fruit drop (PFD), a disease that affects citrus production (Silva et al., 2017), in which necrotic lesions on the petals and premature fruit drop and calyx retention are the main symptoms (Pinho et al., 2015). In Brazil, PFD causes significant production losses, especially in the state of São Paulo, where much of citrus cultivation is concentrated (Pinho et al., 2015; Silva et al., 2017). Therefore, finding endophytic isolates that produce SMs active against this pathogen is of great importance. Additionally, studying endophyte extracts at the compound level can help to characterize the biological activity observed in the crude extract, and provide knowledge for a future application of these compounds.

In addition to the good results observed against *C. abscissum* by the extract of *Diaporthe amolarensis* CMRP4997, those obtained with the metabolites of the isolates *Nemania primolutea* CMRP4987, *Xylaria arbuscula* CMRP5059, and *X. arbuscula* CMRP5050 also showed promising activities. These three isolates belong to the family Xylariaceae, known as an important source of SMs with biological activity (Petrini, 1986; Stadler, 2011; Kuhnert et al., 2014). Members of the Xylariaceae family are normally of cosmopolitan distribution and are mainly endophytes, although can also be found as phytopathogens. However, little is known about the species *N. primolutea* and *X. arbuscula* and their bioactive metabolites. The species *N. primolutea* was described by Ju et al. (2005) been isolated from a dead trunk of *Artocarpus communis* in Taiwan. To date, few studies have demonstrated the biological activity of isolates of this species, such as antibacterial (Tan et al., 2020) and lignolytic (Idris et al., 2019) activities. Recently, a study from our group reported antifungal activity from extracts of this species, with mycelial growth inhibition of *C. abscissum* (MGI: 80%), and *P. citricarpa* (MGI: 76%), as well as inhibition of symptoms development in citrus flowers and leaves (Iantas et al., 2021). *X. arbuscula* species was described by Saccardo (1878), and recently, it was related to the production of the compound cytochalasin B. This compound is a mycotoxin with various biological activities including nuclear cell extrusion, inhibition of HIV-1 protease, and antibiotic and cytotoxic effects, and was also related to the symbiotics endophyte/host interaction (Amaral, 2009; Amaral et al., 2014; Amaral et al., 2017). All these data support the results presented in this study and reaffirm the diverse biological potential of the SMs produced by those strains, especially those isolated from medicinal plants from Brazilian biomes.

The bioactive metabolites produced by the four most promising endophytic fungi strains were subjected to chemical screening analysis, including HPLC-UV and HPLC-MS. These analyses revealed several UV/vis and MS peaks, indicating the production of compounds with great chemical structural diversity. An AntiBase search for the LC-UV/MS peaks detected from some of these metabolites indicated no hits, suggesting that these endophytic fungi may produce new natural products.

Furthermore, we evaluated the cytotoxic activities of the four most promising extracts against A549 (non-small cell lung), PC3 (prostate), and HEL299 (human lung fibroblast) cell lines. The crude extracts produced by *Diaporthe amolarensis* CMRP4997 and *Xylaria arbuscula* CMRP5059 displayed high cytotoxicity across all tested cell lines. The high activity of the extract from *Nemania primolutea* CMRP4987 against HEL299 suggests that the antifungal activity against the phytopathogens observed may be related to the high cytotoxicity against the cancer cell lines. In contrast, the crude extract of CMRP4987 showed promising antifungal activity, but only low or moderate cytotoxicity, indicating a potentially different mechanism of antifungal action (Supplementary Figure S41). Because all biological evaluations were conducted using crude extracts, the detected cytotoxic activity may not be caused by the same pure compounds, which produce the antifungal activity, particularly as the LC-UV/MS data of the crude extracts indicate that these extracts may produce different classes of chemical structures.

Although no extract demonstrated strong activity against *F. graminearum*, the causal agent of Fusarium head blight (FHB), the observed rates of mycelial growth inhibition rates are not negligible, given the difficulty in controlling this pathogen. The best result obtained in this study against this pathogen was achieved with the extract produced from the fermentation of the *X. arbuscula* CMRP5050 (MGI: 23.0%). Additionally, a common strategy for managing this pathogen is the search for compounds that reduce the production of mycotoxins, called anti-mycotoxigenic (Pagnussatt et al., 2014), which should be an approach in future studies. Furthermore, it is important to note that the present study is the first to demonstrate the antifungal activity of extracts produced by an endophyte of the genus *Xylaria* against the phytopathogens *C. abscissum*, *P. citricarpa*, and *F. graminearum*.

In conclusion, this study highlights the Serra do Amolar as an important region of the Pantanal in Brazil, with a rich diversity of endophytes, including the newly described species *D. amolarensis*. Furthermore, extracts from four endophytic fungi demonstrated promising SMs with biological activity against phytopathogens, such as *C. abscissum*, *P. citricarpa*, and *F. graminearum* and should be further explored (scale-up fermentation, isolation of compounds, and purification) to identify bioactive new natural products and their mode of action. Finally, a significant contribution of this study was the *ex situ* conservation of the Serra do Amolar biodiversity, supporting future research and potential biotechnological applications of these endophytes. Such studies add value to Brazilian biodiversity and underscore the importance of conserving these biomes.

## Data Availability

The datasets presented in this study can be found in online repositories. The names of the repository/repositories and accession number(s) can be found in the article/Supplementary material.

## Footnotes

^1^ https://institutoserradoamolar.org.br

^2^ https://www.cmrp-taxonline.com

^3^National Center for Biotechnology Information—http://www.ncbi.nlm.nih.gov/BLAST/.

^4^ http://www.mycobank.org/

^5^ http://www.westerdijkinstitute.nl/

^6^ https://mafft.cbrc.jp/alignment/server/

^7^ http://tree.bio.ed.ac.uk/software/figtree/
