# Supplementary material for: Highly diverse endophytic fungi from Serra do Amolar-Pantanal (Brazil) producing bioactive secondary metabolites against phytopathogens
Source: Front Microbiol. 2024 Dec 24;15:1501182. doi: 10.3389/fmicb.2024.1501182 (PMC11703833; doi:10.3389/fmicb.2024.1501182)
Supplement: Supplementary file 1 [file Data_Sheet_1.docx]

## **Supplementary Material**

**Highly diverse endophytic fungi from Serra do Amolar-Pantanal (Brazil) producing bioactive secondary metabolites against phytopathogens**

Bárbara Fanaya Mayrhofer^1^, Jucélia Iantas^1^, Sandriele Aparecida Noriler^1^, Larissa V. Ponomareva^2,3^, Jon S. Thorson^2,3^, Jürgen Rohr^3*^, Khaled A. Shaaban^2,3,^*; Chirlei Glienke^1,4,*^

^1^Postgraduate Program in Microbiology, Department of Pathology, Federal University of Paraná (UFPR), Centro Politécnico, Curitiba, Paraná, Brazil

^2^Center for Pharmaceutical Research and Innovation, College of Pharmacy, University of Kentucky, Lexington, KY, United States

^3^Department of Pharmaceutical Sciences, College of Pharmacy, University of Kentucky, Lexington, KY, United States

^4^Postgraduate Program in Genetics, Department of Genetics, Federal University of Paraná (UFPR), Centro Politécnico, Curitiba, Paraná, Brazil

**^*^Correspondence:**

Jürgen Rohr

jrohr2@uky.edu

Khaled A. Shaaban

khaled_shaaban@uky.edu

Chirlei Glienke

ch.glienke@gmail.com

| **Contents** | **Page** |
| --- | --- |
| **Table S1.** Sample collection coordinates | 9 |
| **Table S2.** List of taxa, references and GenBank accession numbers of strains of *Diaporthe* included in the study. | 10-12 |
| **Table S3.** List of taxa, references and GenBank accession numbers of strains of *Nemania* included in the study. | 13-14 |
| **Table S4.** List of taxa, references and GenBank accession numbers of strains of the family Xylariaceae included in the study. | 14-17 |
| **Table S5.** List of taxa and GenBank accession of the strains generated by this study. | 17-20 |
| **Table S6.** Taxonomic classification of each phenotype of endophytic fungi isolated in this study from *Vochysia divergens.* | 20-23 |
| **Figure S1.** Macromorphology of each representative of the 91 phenotypes growth in PDA media after 7-14 days of incubation. The isolates are identified with the phenotype code (Phen.) and the culture collection code (CMRP). | 24 |
| **Figure S2.** Bayesian Inference phylogenetic tree of species from Chaetomiaceae family based on the alignment of ITS partial sequence. The data matrix had 81 taxa and 721 characters. The species *Triangularia bambusae* (CBS 35233) was used as an outgroup. Strains marked with a “T” correspond to type sequences. Bayesian posterior probabilities equal to or greater than 0.50 are presented next to each node in bold. The scale bar of 0.05 represents the number of changes. The sequences of the isolates studied here are presented with its culture collection code (CMRP5224, CMRP5062, and CMRP5063) highlighted in bold. | 25 |
| **Figure S3.** Bayesian Inference phylogenetic tree of species from Mycosphaerellaceae family based on the alignment of ITS partial sequence. The data matrix had 19 taxa and 528 characters. The species *Exosporium livistonae* (CBS 131313) was used as an outgroup. Strains marked with a “T” correspond to type sequences. Bayesian posterior probabilities equal to or greater than 0.50 are presented next to each node in bold. The scale bar of 0.03 represents the number of changes. The sequences of the isolates here studied are presented with their culture collection code (CMRP5024 and CMRP5025) highlighted in bold. | 26 |
| **Figure S4.** Bayesian Inference phylogenetic tree of species from Onygenales *insertae sedis* clade 4 (KANDEMIR et al. 2022) based on the alignment of ITS partial sequence. The data matrix had 14 taxa and 789 characters. The species *Nannizziopsis pluriseptata* (UTHSC 10-1045) was used as an outgroup. Strains marked with a “T” correspond to type sequences. Bayesian posterior probabilities equal to or greater than 0.50 are presented next to each node in bold. The scale bar of 0.08 represents the number of changes. The sequences of the isolates studied here are presented with their culture collection code (CMRP5030 and CMRP5064) highlighted in bold. | 27 |
| **Figure S5.** Bayesian Inference phylogenetic tree of *Diaporthe sojae* partial species complex based on the multiple alignment of ITS, *tef1*, *tub2* and *his3* partial sequences. The data matrix had 46 taxa and 2273 characters. The species *Diaporthe perjuncta* (CBS 109745) was used as an outgroup. Strains marked with a “T” correspond to type sequences. Bayesian posterior probabilities equal to or greater than 0.50 are presented next to each node in bold. The scale bar of 0.02 represents the number of changes. The sequence of the isolates studied here are presented with its culture collection code (CMRP4977, CMRP4978, CMRP5220, CMRP5036, CMRP4994, CMRP4996, and CMRP5061) highlighted in bold. | 28 |
| **Figure S6.** Bayesian Inference phylogenetic tree of *Diaporthe sojae* partial species complex based on the alignment of ITS and *tef1* partial sequences. The data matrix had 53 taxa and 1058 characters. The species *Diaporthe perjuncta* (CBS 109745) was used as an outgroup. Strains marked with a “T” correspond to type sequences. Bayesian posterior probabilities equal to or greater than 0.50 are presented next to each node in bold. The scale bar of 0.03 represents the number of changes. The sequence of the isolate studied here is presented with its culture collection code (CMRP4985) highlighted in bold. | 29 |
| **Figure S7.** Bayesian Inference phylogenetic tree of *Diaporthe rudis* species complex based on the alignment of *tef1* partial sequence. The data matrix had 22 taxa and 635 characters. The species *Diaporthe toxica* (CBS 534.93) was used as an outgroup. Strains marked with a “T” correspond to type sequences. Bayesian posterior probabilities equal to or greater than 0.50 are presented next to each node in bold. The scale bar of 0.2 represents the number of changes. The sequences of the studied isolates are presented with its culture collection code (CMRP4989 and CMRP5038) highlighted in bold. | 30 |
| **Figure S8.** Bayesian Inference phylogenetic tree of *Diaporthe arecae* species complex based on the alignment of *tef1* partial sequence. The data matrix had 66 taxa and 385 characters. The species *Diaporthe vawdreyi* (BRIP 57887a) was used as an outgroup. Strains marked with a “T” correspond to type sequences. Bayesian posterior probabilities equal to or greater than 0.50 are presented next to each node in bold. The scale bar of 0.03 represents the number of changes. The sequence of the isolate studied here is presented with its culture collection code (CMRP4990) highlighted in bold. | 31 |
| **Figure S9.** Bayesian Inference phylogenetic tree of *Phyllosticta* species based on multilocus alignment of ITS, *tef1*, *act* and *gapdh* partial sequences. The data matrix had 81 taxa and 2038 characters. The species *Diplodia corticola* (CBS 112549) was used as an outgroup. Strains marked with a “T” correspond to type sequences. Bayesian posterior probabilities equal to or greater than 0.50 are presented next to each node in bold. The scale bar of 0.05 represents the number of changes. The sequences of the isolates studied here are presented with their culture collection codes (CMRP4971, CMRP4972, and CMRP5048) highlighted in bold. | 32 |
| **Figure S10.** Bayesian Inference phylogenetic tree of *Pseudofusicoccum* species based on multiple alignment of ITS and *tef1* partial sequences. The data matrix had 32 taxa and 843 characters. The species *Endomelanconiopsis microspora* (CBS 353.97) was used as an outgroup. Strains marked with a “T” correspond to type sequences. Bayesian posterior probabilities equal to or greater than 0.50 are presented next to each node in bold. The scale bar of 0.02 represents the number of changes. The sequences of the isolates studied here are presented with their culture collection codes (CMRP4982, CMRP5047, CMRP4976, and CMRP5219) highlighted in bold. | 33 |
| **Figure S11.** Bayesian Inference phylogenetic tree of *Colletotrichum acutatum* species complex based on the alignment of ITS partial sequence. The data matrix had 54 taxa and 556 characters. The species *Colletotrichum dematium* (CBS 125.25) was used as an outgroup. Strains marked with a “T” correspond to type sequences. Bayesian posterior probabilities equal to or greater than 0.50 are presented next to each node in bold. The scale bar of 0.004 represents the number of changes. The sequences of the isolates studied here are presented with their culture collection code (CMRP4974, CMRP5222, CMRP5228, and CMRP5221) highlighted in bold. | 34 |
| **Figure S12.** Bayesian Inference phylogenetic tree of *Colletotrichum boninense* species complex based on the alignment of ITS partial sequence. The data matrix had 23 taxa and 560 characters. The species *Colletotrichum truncatum* (CBS 151.35) was used as an outgroup. Strains marked with a “T” correspond to type sequences. Bayesian posterior probabilities equal to or greater than 0.50 are presented next to each node in bold. The scale bar of 0.004 represents the number of changes. The sequences of the isolates studied here are presented with their culture collection codes (CMRP5056, CMRP5039, and CMRP5035) highlighted in bold. | 35 |
| **Figure S13.** Bayesian Inference phylogenetic tree of *Colletotrichum gigasporum* species complex based on the alignment of ITS partial sequence. The data matrix had 16 taxa and 628 characters. The species *Colletotrichum boninense* (MAFF 305972) was used as an outgroup. Strains marked with a “T” correspond to type sequences. Bayesian posterior probabilities equal to or greater than 0.50 are presented next to each node in bold. The scale bar of 0.006 represents the number of changes. The sequence of the isolate here studied is presented with its culture collection code (CMRP5043) highlighted in bold. | 36 |
| **Figure S14.** Bayesian Inference phylogenetic tree of *Colletotrichum gloeosporioides* species complex based on the alignment of ITS partial sequence. The data matrix had 105 taxa and 634 characters. The species *Colletotrichum boninense* (CBS 123755) was used as an outgroup. Strains marked with a “T” correspond to type sequences. Bayesian posterior probabilities equal or greater than 0.50 are presented next to each node in bold. The scale bar of 0.003 represents the number of changes. The sequences of the isolates here studied are presented with their culture collection codes (CMRP4974, CMRP5058, CMRP4984, CMRP4995, CMRP5001, and CMRP4999) highlighted in bold. | 37 |
| **Figure S15.** Bayesian Inference phylogenetic tree of *Nigrospora* species based on the alignment of ITS partial sequence. The data matrix had 51 taxa and 635 characters. The species *Arthrinium malaysianum* (CBS 102053) was used as an outgroup. Strains marked with a “T” correspond to type sequences. Bayesian posterior probabilities equal to or greater than 0.50 are presented next to each node in bold. The scale bar of 0.02 represents the number of changes. The sequences of the isolates studied here are presented with their culture collection code (CMRP5046, CMRP5045, CMRP5044, CMRP5041, CMRP4973, CMRP4975, CMRP4979, and CMRP4980) highlighted in bold. | 38 |
| **Figure S16.** Bayesian Inference phylogenetic tree of *Neopestalotiopsis* species based on the multiple alignment of ITS, *tef1* and *tub2* partial sequence. The data matrix had 56 taxa and 2734 characters. The species *Pestalotiopsis trachicarpicola* (OP068) was used as an outgroup. Strains marked with a “T” correspond to type sequences. Bayesian posterior probabilities equal to or greater than 0.50 are presented next to each node in bold. The scale bar of 0.06 represents the number of changes. The sequences of the isolates studied here are presented with their culture collection codes (CMRP4981 and CMRP5000) highlighted in bold. | 39 |
| **Figure S17.** Bayesian Inference phylogenetic tree of *Cladosporium* species based on the alignment of ITS partial sequence. The data matrix had 103 taxa and 749 characters. The species *Cercospora beticola* (CBS 116456) was used as an outgroup. Strains marked with a “T” correspond to type sequences. Bayesian posterior probabilities equal to or greater than 0.50 are presented next to each node in bold. The scale bar of 0.005 represents the number of changes. The sequences of the isolates studied here are presented with their culture collection codes (CMRP5049, CMRP5032, CMRP5226, and CMRP5021) highlighted in bold. | 40 |
| **Figure S18.** Bayesian Inference phylogenetic tree of *Fusarium* species based on the alignment of ITS partial sequence. The data matrix had 75 taxa and 778 characters. The species *Calonectria citri* (CBS 18636) was used as an outgroup. Strains marked with a “T” correspond to type sequences. Bayesian posterior probabilities equal to or greater than 0.50 are presented next to each node in bold. The scale bar of 0.04 represents the number of changes. The sequences of the isolates studied here are presented with their culture collection codes (CMRP5223 and CMRP5017) highlighted in bold. | 41 |
| **Figure S19.** Bayesian Inference phylogenetic tree of species from Chryphonectriaceae family based on the alignment of ITS partial sequence. The data matrix had 36 taxa and 620 characters. The species *Diaporthe ambigua* (CBS 127746) was used as an outgroup. Strains marked with a “T” correspond to type sequences. Bayesian posterior probabilities equal to or greater than 0.50 are presented next to each node in bold. The scale bar of 0.06 represents the number of changes. The sequences of the isolates studied here are presented with their culture collection codes (CMRP5022 and CMRP5057) highlighted in bold. | 42 |
| **Figure S20.** Bayesian Inference phylogenetic tree of *Polyscytalum* species based on the alignment of ITS partial sequence. The data matrix had 10 taxa and 957 characters. The species *Cylindrium corymbiae* (CPC 35637) was used as an outgroup. Strains marked with a “T” correspond to type sequences. Bayesian posterior probabilities equal to or greater than 0.50 are presented next to each node in bold. The scale bar of 0.05 represents the number of changes. The sequence of the isolate here studied is presented with its culture collection code (CMRP5225) highlighted in bold. | 43 |
| **Figure S21.** Bayesian Inference phylogenetic tree of *Hypoxylon* species based on the alignment of ITS partial sequence. The data matrix had 77 taxa and 956 characters. The species *Xylariaceae* sp. 3 (LGMF 1542) was used as an outgroup. Strains marked with a “T” correspond to type sequences. Bayesian posterior probabilities equal to or greater than 0.50 are presented next to each node in bold. The scale bar of 0.2 represents the number of changes. The sequences of the isolates studied here are presented with their culture collection codes (CMRP5016 and CMRP5053) highlighted in bold. | 44 |
| **Figure S22.** Bayesian Inference phylogenetic tree of *Neurospora* species based on the alignment of ITS partial sequence. The data matrix had 32 taxa and 633 characters. The species *Chaetomium globosum* (CBS 161.52) was used as an outgroup. Strains marked with a “T” correspond to type sequences. Bayesian posterior probabilities equal to or greater than 0.50 are presented next to each node in bold. The scale bar of 0.008 represents the number of changes. The sequences of the isolates studied here are presented with their culture collection codes (CMRP5029, CMRP5018, and CMRP5019) highlighted in bold. | 45 |
| **Figure S23.** Bayesian Inference phylogenetic tree of *Curvularia* species based on the alignment of ITS partial sequence. The data matrix had 122 taxa and 900 characters. The species *Bipolaris maydis* (CBS 136.29) was used as an outgroup. Strains marked with a “T” correspond to type sequences. Bayesian posterior probabilities equal to or greater than 0.50 are presented next to each node in bold. The scale bar of 0.08 represents the number of changes. The sequences of the isolates studied here are presented with their culture collection codes (CMRP4991 and CMRP5040) highlighted in bold. | 46 |
| **Figure S24.** Bayesian Inference phylogenetic tree of *Paecilomyces* species based on the alignment of ITS partial sequence. The data matrix had 36 taxa and 770 characters. The species *Sclerocleista ornata* (NRRL 2291) was used as an outgroup. Strains marked with a “T” correspond to type sequences. Bayesian posterior probabilities equal to or greater than 0.50 are presented next to each node in bold. The scale bar of 0.09 represents the number of changes. The sequence of the isolate here studied is presented with its culture collection code (CMRP5052) highlighted in bold. | 47 |
| **Figure S25.** Bayesian Inference phylogenetic tree of *Periconia* species based on the alignment of ITS partial sequence. The data matrix had 26 taxa and 1326 characters. The species *Paraphaeosphaeria michotii* (MFLUCC 13-0349) was used as an outgroup. Strains marked with a “T” correspond to type sequences. Bayesian posterior probabilities equal to or greater than 0.50 are presented next to each node in bold. The scale bar of 0.04 represents the number of changes. The sequence of the isolate here studied is presented with its culture collection code (CMRP5033) highlighted in bold. | 48 |
| **Figure S26.** Bayesian Inference phylogenetic tree of *Pestalotiopsis* species based on the alignment of ITS partial sequence. The data matrix had 93 taxa and 634 characters. The species *Neopestalotiopsis saprophytica* (MFLUCC 12-0282) was used as an outgroup. Strains marked with a “T” correspond to type sequences. Bayesian posterior probabilities equal to or greater than 0.50 are presented next to each node in bold. The scale bar of 0.007 represents the number of changes. The sequence of the isolate here studied is presented with its culture collection code (CMRP5037) highlighted in bold. | 49 |
| **Figure S27.** Bayesian Inference phylogenetic tree of *Pseudocercospora* species based on the alignment of ITS partial sequence. The data matrix had 172 taxa and 583 characters. The species *Passalora eucalypti* (CBS 111318) was used as an outgroup. Strains marked with a “T” correspond to type sequences. Bayesian posterior probabilities equal to or greater than 0.50 are presented next to each node in bold. The scale bar of 0.01 represents the number of changes. The sequence of the isolate here studied is presented with its culture collection code (CMRP5027) highlighted in bold. | 50-51 |
| **Figure S28.** Bayesian Inference phylogenetic tree of *Phaeophleospora* species based on the alignment of ITS partial sequence. The data matrix had 22 taxa and 714 characters. The species *Lecanosticta brevispora* (CBS 133601) was used as an outgroup. Strains marked with a “T” correspond to type sequences. Bayesian posterior probabilities equal to or greater than 0.50 are presented next to each node in bold. The scale bar of 0.06 represents the number of changes. The sequence of the isolate here studied is presented with its culture collection code (CMRP5026) highlighted in bold. | 52 |
| **Figure S29.** Bayesian Inference phylogenetic tree of *Talaromyces* section *Talaromyces* species based on the alignment of ITS partial sequence. The data matrix had 55 taxa and 787 characters. The species *Talaromyces trachyspermus* (CBS 37348) was used as an outgroup. Strains marked with a “T” correspond to type sequences. Bayesian posterior probabilities equal to or greater than 0.50 are presented next to each node in bold. The scale bar of 0.01 represents the number of changes. The sequence of the isolate here studied is presented with its culture collection code (CMRP5020) highlighted in bold. | 53 |
| **Figure S30.** Bayesian Inference phylogenetic tree of *Alternaria* section *Alternaria* species based on the alignment of ITS partial sequence. The data matrix had 15 taxa and 570 characters. The species *Alternaria protae* (CBS 47590) was used as an outgroup. Strains marked with a “T” correspond to type sequences. Bayesian posterior probabilities equal to or greater than 0.50 are presented next to each node in bold. The scale bar of 0.003 represents the number of changes. The sequence of the isolate here studied is presented with its culture collection code (CMRP5051) highlighted in bold. | 54 |
| **Figure S31.** Bayesian Inference phylogenetic tree of *Aspergillus* section *Nigri* species based on the alignment of ITS partial sequence. The data matrix had 32 taxa and 641 characters. The species *Penicillium expansum* (CBS 325.48) was used as an outgroup. Strains marked with a “T” correspond to type sequences. Bayesian posterior probabilities equal to or greater than 0.50 are presented next to each node in bold. The scale bar of 0.02 represents the number of changes. The sequence of the isolate here studied is presented with its culture collection code (CMRP5218) highlighted in bold. | 55 |
| **Figure S32.** Bayesian Inference phylogenetic tree of *Biscogniauxia* species based on the alignment of ITS partial sequence. The data matrix had 29 taxa and 1055 characters. The species *Xylaria hypoxylon* (CBS 122620) was used as an outgroup. Strains marked with a “T” correspond to type sequences. Bayesian posterior probabilities equal to or greater than 0.50 are presented next to each node in bold. The scale bar of 0.2 represents the number of changes. The sequence of the isolate here studied is presented with its culture collection code (CMRP5023) highlighted in bold. | 56 |
| **Figure S33.** Bayesian Inference phylogenetic tree of *Corynespora* species based on the alignment of ITS partial sequence. The data matrix had 15 taxa and 1067 characters. The species *Cyclothyriella rubronotata* (CBS 121892) was used as an outgroup. Strains marked with a “T” correspond to type sequences. Bayesian posterior probabilities equal to or greater than 0.50 are presented next to each node in bold. The scale bar of 0.05 represents the number of changes. The sequence of the isolate here studied is presented with its culture collection code (CMRP5054) highlighted in bold. | 57 |
| **Figure S34.** Bayesian Inference phylogenetic tree of *Daldinia* species based on the alignment of ITS partial sequence. The data matrix had 31 taxa and 989 characters. The species *Pyrenopolyporus nicaraguense* (CBS 117739) was used as an outgroup. Strains marked with a “T” correspond to type sequences. Bayesian posterior probabilities equal to or greater than 0.50 are presented next to each node in bold. The scale bar of 0.02 represents the number of changes. The sequence of the isolate here studied is presented with its culture collection code (CMRP5028) highlighted in bold. | 58 |
| **Figure S35.** Bayesian Inference phylogenetic tree of *Endomelanconiopsis* species based on the alignment of ITS partial sequence. The data matrix had 5 taxa and 658 characters. The species *Neofusicoccum parvum* (CMW 9081) was used as an outgroup. Strains marked with a “T” correspond to type sequences. Bayesian posterior probabilities equal to or greater than 0.50 are presented next to each node in bold. The scale bar of 0.009 represents the number of changes. The sequence of the isolate here studied is presented with its culture collection code (CMRP5042) highlighted in bold. | 59 |
| **Figure S36.** Bayesian Inference phylogenetic tree of *Lasiodiplodia* species based on multiple alignment of ITS, *tef1* and *tub2* partial sequence. The data matrix had 76 taxa and 2,085 characters. The species *Diplodia mutila* (CMW 7060) was used as an outgroup. Strains marked with a “T” correspond to type sequences. Bayesian posterior probabilities equal to or greater than 0.50 are presented next to each node in bold. The scale bar of 0.03 represents the number of changes. The sequence of the isolate here studied is presented with its culture collection code (CMRP4998) highlighted in bold. | 60 |
| **Figure S37.** Bayesian Inference phylogenetic tree of *Penicillium* section *Citrina* species based on the alignment of ITS partial sequence. The data matrix had 45 taxa and 889 characters. The species *Aspergillus glaucus* (NRRL 116) was used as an outgroup. Strains marked with a “T” correspond to type sequences. Bayesian posterior probabilities equal to or greater than 0.50 are presented next to each node in bold. The scale bar of 0.2 represents the number of changes. The sequence of the isolate here studied is presented with its culture collection code (CMRP5227) highlighted in bold. | 61 |
| **Figure S38.** Bayesian Inference phylogenetic tree of species from Prosopidicolaceae and Stilbosporaceae families based on the alignment of ITS partial sequence. The data matrix had 10 taxa and 681 characters. The species *Diaporthe eres* (AR3519) was used as an outgroup. Strains marked with a “T” correspond to type sequences. Bayesian posterior probabilities equal to or greater than 0.50 are presented next to each node in bold. The scale bar of 0.03 represents the number of changes. The sequence of the isolate here studied is presented with its culture collection code (CMRP5031) highlighted in bold. | 62 |
| **Figure S39.** Bayesian Inference phylogenetic tree of *Pseudopestalotiopsis* species based on the alignment of ITS partial sequence. The data matrix had 30 taxa and 615 characters. The species *Neopestalotiopsis protearum* (CBS 114178) was used as an outgroup. Strains marked with a “T” correspond to type sequences. Bayesian posterior probabilities equal to or greater than 0.50 are presented next to each node in bold. The scale bar of 0.003 represents the number of changes. The sequence of the isolate here studied is presented with its culture collection code (CMRP5060) highlighted in bold. | 63 |
| **Figure S40.** Bayesian Inference phylogenetic tree of species from Barbatosphaeriaceae family based on the alignment of ITS partial sequence. The data matrix had 19 taxa and 611 characters. The species *Annulusmagnus triseptatus* (CBS 127688) was used as an outgroup. Strains marked with a “T” correspond to type sequences. Bayesian posterior probabilities equal to or greater than 0.50 are presented next to each node in bold. The scale bar of 0.03 represents the number of changes. The sequence of the isolate here studied is presented with its culture collection code (CMRP4986) highlighted in bold. | 64 |
| **Table S7.** GenBank megablast result for the first 10 sequences using ITS (internal transcribed spacer) partial sequence from CMRP4997 isolate. | 64-65 |
| **Table S8.** GenBank megablast result for the first 10 sequences using *tef1* (translation elongation factor 1-α) partial sequence from CMRP4997 isolate. | 65 |
| **Table S9.** GenBank megablast result for the first 10 sequences using *tub* (tubulin) partial sequence from CMRP4997 isolate. | 65 |
| **Table S10.** GenBank megablast result for the first 10 sequences using *his3* (histone H3) partial sequence from CMRP4997 isolate. | 65-66 |
| **Figure S41:** % Viability of A549 (non-small lung), PC3 (prostate) and HEL299 (human lung fibroblast) cell lines (after 72h) at 1:25 dilution of the fungal extracts produced by *Diaporthe* *amolarensis* sp. nov. CMRP4997, *Nemania primolutea* CMRP4987, *Xylaria arbuscula* CMRP5059, *Xylaria arbuscula* CMRP5050. | 66 |
| **Figures S42-66.** HPLC-UV/MS analysis of fungal extracts produced by *Diaporthe* *amolarensis* sp. nov. CMRP4997, *Nemania primolutea* CMRP4987, *Xylaria arbuscula* CMRP5059, *Xylaria arbuscula* CMRP5050. LC-MS conditions: H_2_O/0.1% formic acid (solvent A), CH_3_CN/0.1% formic acid (solvent B); flow rate: 0.5 mL min^-1^; 0-2 min, 5% B; 2-30 min, 5-100% B; 30-35 min, 100% B; 35-36 min, 100%-5% B; 36-40 min, 5% B. | 67-91 |
| **References** | 92-94 |

**Table S1.** Sample collection coordinates.

| **Plant** | **Coordinates** |
| --- | --- |
| Plant 1 | 18º29'54.7"S 57º27'16.5"W |
| Plant 2 | 18°29'54.5"S 57°27'20.0"W |
| Plant 3 | 18°30'22.2"S 57°27'56.1"W |
| Plant 4 | 18°29'06.6"S 57°27'05.6"W |
| Plant 5 | 18°28'59.3"S 57°27'13.5"W |
| Plant 6 | 18º28'10.5"S 57º22'10.2"W |
| Plant 7 | 18°15'37.8"S 57°27'37.4"W |
| Plant 8 | 18°15'39.4"S 57°27'35.6"W |
| Plant 9 | 18°15'45.1"S 57°26'11.3"W |
| Plant 10 | 18°15'42.3"S 57°27'29.9"W |
| Plant 11 | 18°15'25.6"S 57°27'42.0"W |
| Plant 11.2 | 18°15'29.7"S 57°27'33.6"W |
| Plant 12 | 18°13'00.0"S 57°22'32.0"W |
| Plant 13 | 18°22'55.8"S 57°20'55.8"W |
| Plant 14 | 18°22'55.8"S 57°20'55.8"W |
| Plant 15 | 18°15'35.5"S 57°27'28.7"W |
| Plant 16 | 18°15'36.3"S 57°27'28.3"W |
| Plant 17 | 18°10'04.5"S 57°23'01.0"W |

**Table S2.** List of taxa, references and GenBank accession numbers of strains of *Diaporthe* included in the study.

|  |  |  |  | **GenBank accession no.^2^** | | | |  |
| --- | --- | --- | --- | --- | --- | --- | --- | --- |
| **Species** | **Collection number^1^** | **Host** | **Country** | **ITS** | ***tub2*** | ***tef1*** | ***his3*** | **Reference** |
| ***Diaporthe* *amolarensis*** | **CMRP4997** | ***Vochysia divergens*** | **Brazil** | **MZ567074** | **ON675581** | **ON564444** | - | **This study** |
| *D. anacardii* | CBS 720.97* | *Anacardium occidentale* | East Africa | KC343024 | KC343992 | KC343750 | KC343508 | Gomes et al*.* (2013) |
|  | CBS 144610 | *unidentified leaf* | South Africa | MK442578 | - | MK442692 | - | Crous et al*.* (2019a) |
| *D. baccae* | CBS 136972* | *Vaccinium corymbosum* | Italy | KJ160565 | MF418509 | KJ160597 | MF418264 | Lombard et al. (2014) |
| *D. camelliae-sinensis* | SAUCC 194.92* | *Camellia sinensis* | China | MT822620 | MT855817 | MT855932 | MT855588 | Huang et al. (2020) |
|  | SAUCC 194.103 | *Castanea mollissima* | China | MT822631 | MT855828 | MT855943 | MT855599 | Huang et al. (2020) |
| *D. canthii* | CBS 132533* | *Canthium inerme* | South Africa | JX069864 | KC843230 | KC843120 | - | Udayanga et al*.* (2014) |
| *D. caulivora* | CBS 127268* | *Glycine max* | Croatia | KC343045 | KC344013 | KC343771 | KC343529 | Gomes et al. (2013) |
| *D. chamaeropsis* | CBS 454.81* | *Chamaeropsis humilis* | Greece | KC343048 | KC344016 | KC343774 | KC343532 | Gomes et al. (2013) |
|  | CBS 753.70 | *Spartium junceum* | Croatia | KC343049 | KC344017 | KC343775 | KC343533 | Gomes et al. (2013) |
| *D. cinerascens* | CBS 719.96 | *Ficus carica* | Bulgaria | KC343050 | KC344018 | KC343776 | KC343534 | Gomes et al. (2013) |
| *D. cissampeli* | CBS 141331* | *Cissampelus capensis* | South Africa | KX228273 | KX228384 | - | KX228366 | Crous et al. (2013) |
| *D. corylicola* | CFCC 53986* | *Corylus heterophylla* | China | MW839880 | MW883977 | MW815894 | MW836717 | Gao et al. (2021) |
| *D. cytosporella* | CBS 137020* | *Citrus limon* | Spain | KC843307 | KC843221 | KC843116 | MF418283 | Udayanga et al*.* (2014) |
| *D. elaeagni-glabrae* | CGMCC 3.18287* | *Elaeagnus glabra* | China | KX986779 | KX999212 | KX999171 | KX999251 | Gao et al. (2017) |
|  | LC4806 | *Elaeagnus glabra* | China | KX986780 | KX999213 | KX999172 | KX999252 | Gao et al. (2017) |
| *D. foeniculacea* | CBS 111553* | *Foeniculum vulgare* | Spain | KC343101 | KC344069 | KC343827 | KC343585 | Gomes et al*.* (2013) |
| *D. forlicesenica* | MFLUCC 17-1015* | *Dorycnium hirsutum* | Italy | KY964215 | KY964099 | KY964171 | - | Dissanayake et al. (2017) |
| *D. hickoriae* | CBS 145.26* | *Carya glabra* | USA | KC343118 | KC344086 | KC343844 | KC343602 | Gomes et al*.* (2013) |
| *D. inconspicua* | LGMF922 | *Spondias mombin* | Brazil | KC343124 | KC344092 | KC343850 | KC343608 | Gomes et al*.* (2013) |
|  | CBS 133813* | *Maytenus ilicifolia* | Brazil | KC343123 | KC344091 | KC343849 | KC343607 | Gomes et al*.* (2013) |
| *D. isoberline* | CPC 22549* | *Isoberlinia angolensis* | Zambia | KJ869133 | KJ869245 | - | - | Crous et al. (2014) |
| *D. lutescens* | SAUCC 194.36* | *Chrysalidocarpus lutescens* | China | MT822564 | MT855761 | MT855877 | MT855533 | Huang et al. (2020) |
| *D. machintoshii* | BRIP 55064a* | *Rapistrum rugostrum* | Australia | KJ197289 | KJ197269 | KJ197251 | - | Thompson et al. (2015) |
| *D. maytenicola* | CPC 21896* | *Maytenus acuminata* | South Africa | KF777157 | KF777250 | - | - | Crous et al. (2013) |
| *D. melastomatis* | SAUCC 194.55* | *Melastoma malabathricum* | China | MT822583 | MT855780 | MT855896 | MT855551 | Huang et al. (2020) |
| *D. nebulae* | PMM1681* | *Vitis vinifera* | South Africa | KY511337 | KY511369 | MH708552 | - | Lesuthu et al. (2017) |
|  | Phom240 | *Vitis vinifera* | South Africa | KY511315 | KY511346 | MH708543 | - | Lesuthu et al. (2017) |
| *D. nigra* | JZB320170* | *Ballota nigra* | Italy | MN653009 | MN887113 | MN892277 | - | Hyde and Brahmanage (2019) |
| *D. oncostoma* | CBS 589.78 | *Robinia pseudoacacia* | France | KC343162 | KC344130 | KC343888 | KC343646 | Gomes et al. (2013) |
|  | CBS 100454 | *Robinia pseudoacacia* | Germany | KC343160 | KC344128 | KC343886 | KC343644 | Gomes et al. (2013) |
| *D. parapterocarpi* | CPC 22729* | *Pterocarpus brenanii* | Zambia | KJ869138 | KJ869248 | - | - | Crous et al. (2014) |
| *D. parva* | PSCG 034* | *Pyrus bretschneideri* | China | MK626919 | MK691248 | MK654858 | MK726210 | Guo et al. (2020) |
|  | PSCG 035 | *Pyrus bretschneideri* | China | MK626920 | MK691249 | MK654859 | MK726211 | Guo et al. (2020) |
| *D. phillipsi* | CAA 817 | *Vaccinium corymbosum* | Portugal | MK792305 | MN000351 | MK828076 | MK871445 | Hilário et al*.* (2020) |
|  | CAA 818 | *Vaccinium corymbosum* | Portugal | MK792307 | MN000352 | MK828078 | MK871447 | Hilário et al*.* (2020) |
| *D. poincianellae* | URM 7932* | *Cenostigma pyramidale* | Brazil | MH989509 | MH989537 | MH989538 | MH989539 | Bezerra (2018) |
| *D. portugallica* | CPC 34247* | *Camellia sinensis* | Portugal | MH063905 | MH063917 | MH063911 | MH063899 | Guarnaccia, Crous (2018) |
|  | CPC 34248 | *Camellia sinensis* | Portugal | MH063906 | MH063918 | MH063912 | MH063900 | Guarnaccia, Crous (2018) |
| *D. pseudoinconspicua* | URM 7874* | *Cenostigma pyramidale* | Brazil | MH122538 | MH122524 | MH122533 | MH122517 | Bezerra (2018) |
| *D. psoreleae* | CBS 136412* | *Psoralea pinnata* | South Africa | KF777158 | KF777251 | KF777245 | - | Crous et al. (2013) |
| *D. pterocarpi* | MFLUCC 10-0571* | *Pterocarpus indicus* | Thailand | JQ619899 | JX275460 | JX275416 | - | Udayanga et al*.* (2012) |
|  | MFLUCC 10-0588 | *Pterocarpus indicus* | Thailand | JQ619900 | JX275461 | JX275417 | - | Udayanga et al*.* (2012) |
| *D. pungensis* | SAUCC 194.112* | *Elaeagnus pungensis* | China | MT822640 | MT855837 | MT855952 | MT855607 | Huang et al. (2020) |
| *D. ravennica* | MFLUCC 15-0480 | *Tamarix* sp. | Italy | KU900336 | KX377688 | KX426703 | - | Thambugala et al. (2016) |
| *D. ravennica* | MFLUCC 15-0479* | *Tamarix* sp. | Italy | KU900335 | KX432254 | KX365197 | - | Thambugala et al. (2016) |
| *D. rhusicola* | CBS 129528* | *Rhus pendulina* | South Africa | JF951146 | KC843205 | KC843100 | - | Crous et al. (2011) |
| *D. rumicicola* | JZB320006* | *Scrophularia canina* | Italy | MK066126 | MK078546 | MK078545 | - | Aluthmuhandiram (2020) |
| *D. saccarata* | CBS 116311* | *Protea repens* | South Africa | KC343190 | KC344158 | KC343916 | KC343674 | Gomes et al. (2013) |
| *Diaporthe* sp. | CMRP4330 | *Vochysia divergens* | Brazil | [MN173199](https://www.ncbi.nlm.nih.gov/nuccore/MN173193) | MW751670 | MT311684 | MW751662 | Iantas et al. (2021) |
| ***Diaporthe* sp.** | **CMRP5034** | *Vochysia divergens* | Brazil | **ON325550** | - | **ON564445** | - | **This study** |
| *D. stictica* | CBS 370.54* | *Buxus sampervirens* | Italy | KC343212 | KC344180 | KC343938 | KC343696 | Gomes et al. (2013) |
| *D. vangueriae* | CPC 22703* | *Vangueria infausta* | Zambia | KJ869137 | KJ869247 | - | - | Crous et al. (2014) |
| *D. velutina* | LC442/ CGMCC 3.18286* | *Neolitsea* sp. | China | KX986790 | KX999223 | KX999182 | KX999261 | Gao et al*.* (2017) |
|  | PSCG 134 | *Pyrus pyrifolia* | China | MK626918 | MK691243 | MK654853 | MK726205 | Guo et al. (2020) |
| *D. zaobaisu* | PSCG 031* | *Pyrus bretschneideri* | China | MK626922 | MK691245 | MK654855 | MK726207 | Guo et al. (2020) |
|  | PSCG 032 | *Pyrus bretschneideri* | China | MK626923 | MK691246 | - | - | Guo et al. (2020) |
| ^1^Collection – Type strains included in analysis are indicated with *. Strains marked in bold were those generated by this study.  Culture collections abbreviations: BRIP = Australian plant pathogen culture collection, Queensland, Australia; CAA = Personal Culture Collection of Artur Alves, Universidade de Aveiro, Portugal; CBS = Culture Collection of the Westerdijk Fungal Biodiversity Institute, Utrecht, The Netherlands; CGMCC = China General Microbiological Culture Collection; CMRP = Microbiological Collections of Paraná Network, Federal University of Paraná, Curitiba, Brazil; CPC = Culture Collection of Pedro Crous, housed at CBS; LC = Working collection of Lei Cai, housed at Institute of Microbiology, CAS, China; LGMF = Culture Collection of Laboratory of Genetics of Microorganisms, Federal University of Paraná, Curitiba, Brazil; MFLUCC = Mae Fah Luang University Culture Collection;  ^2^GenBank - *ITS:* internal transcribed spacers and intervening 5.8S nrDNA; *tef1*: translation elongation factor 1-α; *tub2:* partial beta-tubulin gene; *his3:* partial histone H3 gene.  - : Sequence not available | | | | | | | | |

**Table S3.** List of taxa, references and GenBank accession numbers of strains of *Nemania* included in the study.

|  |  |  |  | **GenBank accession no.^2^** | | |  |
| --- | --- | --- | --- | --- | --- | --- | --- |
| **Species** | **Collection number^1^** | **Host** | **Country** | **ITS** | ***tub2*** | ***act*** | **Reference** |
| *Biscogniauxia arima* | YMJ 122 | Wood | Mexico | EF026150 | AY951672 | AY951784 | Hsieh et al. (2010) |
| *Nemania abortiva* | BISH 467* | Decayed angiosperm wood | USA | GU292816 | GQ470219 | GQ374123 | Hsieh et al. (2010) |
| *N. abortiva* | ATCC MYA-4108 | - | - | FJ172270 | - | - | Houseknecht et al. (2016) |
| *N. aenea var. aureolatum* | ATCC 60819 | - | - | AF201704 | - | - | Pinto-Sherer and Chapela et al. (2000) |
| *N. beaumontii* | HAST 405 | Bark | Taiwan | GU292819 | GQ470222 | GQ389694 | Hsieh et al. (2010) |
| *N. bipapillata* | HAST 90080610 | Bark | Taiwan | GU292818 | GQ470221 | GQ389693 | Hsieh et al. (2010) |
| *N. chestersii* | JF04024 | - | - | - | DQ840089 | - | Tang et al. (2009) |
| *N. diffusa* | HAST 91020401 | *Castanopsis carlesii* var. *sessilis* | Taiwan | GU292817 | GQ470220 | GQ389692 | Hsieh et al. (2010) |
| *N. illita* | YMJ 236 | Wood | USA | EF026122 | EF025608 | EF025593 | Hsieh et al. (2010) |
| *N. macrocarpa* | WSP 265 | *Sapindus saponaria* | USA | GU292823 | GQ470226 | GQ389698 | Hsieh et al. (2010) |
| *N. macrocarpa* | CBS109567 | - | USA | MH862830 | - | - | Vu et al. (2019) |
| *N. maritima* | HAST 89120401 | *Kandelia candel* | Taiwan | GU292822 | GQ470225 | GQ389697 | Hsieh et al. (2010) |
| *N. phetchaburiensis* | MFLU 16-1185 | - | - | MN047124 | - | - | Dayarathne et al. (2020) |
| *N. plumbea* | 6540 | *Pholidota* sp. | China | JQ846087 | - | - | Tan and Guo (2012) |
|  | JF-TH-04-01 | - | Thailand | DQ641634 | - | - | Tang et al. (2007) |
| *N. pouzarii* | ATCC 2612 | - | - | KC477228 | - | - | Stadler et al. (2013) |
| ***N. primolutea*** | **CMRP4987** | ***Vochysia divergens*** | **Brazil** | **ON325485** | **ON623731** | - | **This study** |
|  | YMJ 91102001 | *Artocarpus communis* | Taiwan | EF026121 | EF025607 | EF025592 | Hsieh et al. (2010) |
|  | CMRP4323 | *Stryphnodendron adstringens* | Brazil | [MN173201](https://www.ncbi.nlm.nih.gov/nuccore/MN173193) | - | - | Iantas et al. (2021) |
| *N. serpens* | HAST 235 | Soil | Canada | GU292820 | GQ470223 | GQ389695 | Hsieh et al. (2010) |
|  | N20A | - | - | AJ390431 | - | - | Sánchez-Ballesteros et al. (2000) |
| *N. serpens var. macrospora* | N21A | - | - | AJ390433 | - | - | Sánchez-Ballesteros et al. (2000) |
| *N. serpens var. serpens* | CBS 659.70 | - | Canada | MH859890 | - | - | Vu et al. (2019) |
| *N. viridis* | MFLU 17-2600* | - | - | MN047123 | - | - | Dayarathne et al. (2020) |
| ^1^Collection – Type strains included in analysis are indicated with *. Strains marked in bold were those generated by this study.  Culture collections abbreviations: ATCC, American Type Culture Collection, Manassas, USA; BISH, Bishop Museum, Honolulu, USA; CBS = Culture Collection of the Westerdijk Fungal Biodiversity Institute, Utrecht, The Netherlands; CMRP = Microbiological Collections of Paraná Network, Federal University of Paraná, Curitiba, Brazil; HAST, Academia Sinica, Taipei, Taiwan; Y.M.J., Yu-Ming Ju, Academia Sinica, Taipei, Taiwan; WSP, Washington State University, Pullman, USA;  ^2^GenBank - *ITS:* internal transcribed spacers and intervening 5.8S nrDNA; *tub2:* partial beta-tubulin gene; *act:* partial actin gene.  - : Sequence not available | | | | | | | |

**Table S4.** List of taxa, references and GenBank accession numbers of strains of the family Xylariaceae included in the study.

|  |  |  |  | **GenBank accession no.^2^** | | | |  |
| --- | --- | --- | --- | --- | --- | --- | --- | --- |
| **Species** | **Collection number^1^** | **Host** | **Country** | **ITS** | ***LSU*** | ***rpb2*** | ***tub2*** | **Reference** |
| *Amphirosellinia fushanensis* | HAST 91111209 | *Quercus longinux* | Taiwan | GU339496 | - | GQ848339 | GQ495950 | Hsieh et al. (2010) |
| *A. nigrospora* | HAST 91092308 | - | Taiwan | GU322457 | - | GQ848340 | GQ495951 | Hsieh et al. (2010) |
| *Astrocystis concavispora* | MFLUCC 14-0174 | *Arundo donax* | Italy | KP297404 | KP340545 | KP340532 | KP406615 | Daranagama et al. (2015) |
| *Barrmaelia rappazii* | CBS 142771 | *Populus tremula* | Norway | MF488989 | MF488989 | MF488998 | MF489017 | Voglmayr et al. (2017) |
| *Brunneiperidium gracilentum* | MFLUCC 14-0011 | *Tamarix gallica* | Italy | KP297400 | KP340542 | KP340528 | KP406611 | Daranagama et al. (2015) |
| *Clypeosphaeria mamillana* | CBS 140735 | *Cornus alba* | France | KT949897 | KT949897 | MF489001 | - | Jaklitsch et al. (2016) |
| *Collodiscula bambusae* | GZUH 0102 | Bamboo | China | KP054279 | KP054280 | KP276675 | KP276674 | Li et al. (2014) |
| *C. fangjingshanensis* | GZUH 0109 | Bamboo stalk | China | KR002590 | KR002591 | KR002592 | KR002589 | Li et al. (2014) |
| *C. japonica* | CBS 124266 | - | - | JF440974 | JF440974 | KY624273 | KY624316 | Jaklitsch Voglmayr (2012) |
| *Entoleuca mammata* | J.D.R. 100 | Fagus | France | GU300072 | - | GQ844782 | GQ470230 | Hsieh et al. (2010) |
| *Euepixylon sphaeriostomum* | J.D.R. 261 | Fraxinus | USA | GU292821 | - | GQ844774 | GQ470224 | Hsieh et al. (2010) |
| *Hypocreodendron sanguineum* | J.D.R. 169 | Nests of Atta mexicana and rotten wood | Mexico | GU322433 | - | GQ844819 | GQ487710 | Hsieh et al. (2010) |
| *Kretzschmaria deusta* | CBS 163.93 | - | - | KC477237 | KY610458 | KY624227 | KX271251 | Stadler et al. (2013) |
| *Nemania abortiva* | BISH 467 T | Decayed angiosperm wood | USA | GU292816 | - | GQ844768 | GQ470219 | Hsieh et al. (2010) |
| *N. beaumontii* | HAST 405 | Bark | Martinique | GU292819 | - | GQ844772 | GQ470222 | Hsieh et al. (2010) |
| *N. bipapillata* | HAST 90080610 | Bark | Taiwan | GU292818 | - | GQ844771 | GQ470221 | Hsieh et al. (2010) |
| *N. maritima* | ST.MA. 04019 | - | France | KY610414 | KY610414 | - | - | Wendt et al. (2018) |
| *N. primolutea* | HAST 91102001 | *Artocarpus communis* | Taiwan | EF026121 | - | GQ844767 | EF025607 | Hsieh et al. (2010) |
| *Podosordaria muli* | WSP 167 | - | Mexico | GU324761 | - | GQ853038 | GQ844839 | Hsieh et al. (2010) |
| *Poronia pileiformis* | WSP 88113001 | Cow dung | Taiwan | GU324760 | - | GQ853037 | GQ502720 | Hsieh et al. (2010) |
| *P. punctata* | CBS 656.78 | Dung of horse | Australia | KT281904 | KY610496 | KY624278 | KX271281 | Senanayake et al. (2015) |
| *Rosellinia aquila* | MUCL 51703 | - | France | KY610392 | KY610460 | KY624285 | KX271253 | Wendt et al. (2018) |
| *R. buxi* | J.D.R. 99 | *Buxus sempervirens* | France | GU300070 | - | GQ844780 | GQ470228 | Hsieh et al. (2010) |
| *R. corticium* | MUCL 51693 | - | France | KY610393 | KY610461 | KY624229 | KX271254 | Wendt et al. (2018) |
| *R. necatrix* | CBS 349.36 | - | - | AY909001 | KF719204 | KY624275 | KY624310 | Peláez et al. (2008) |
| *Sarcoxylon compunctum* | CBS 359.61 | River water | South Africa | KT281903 | KY610462 | KY624230 | KX271255 | Senanayake et al. (2015) |
| *Stilbohypoxylon elaeicola* | Y.M.J. 173 | Decorticated wood | French Guiana | EF026148 | - | GQ844826 | EF025616 | Hsieh et al. (2010) |
| *Xylaria acuminatilongissima* | HAST 95060506 | Ground of bamboo plantation | Taiwan | EU178738 | - | GQ853028 | GQ502711 | Hsieh et al. (2010) |
| ***Xylaria adscendens*** | **CMRP4993** | ***Vochysia divergens*** | **Brazil** | **ON325488** | **ON325576** | - |  | **This study** |
|  | J.D.R. 865 | Wood | Thailand | GU322432 | - | GQ844818 | GQ487709 | Hsieh et al. (2010) |
| ***Xylaria arbuscula*** | **CMRP5050** | ***Vochysia divergens*** | **Brazil** | **ON325489** | **ON325577** | - |  | **This study** |
| ***Xylaria arbuscula*** | **CMRP5059** | ***Vochysia divergens*** | **Brazil** | **ON325486** | - | - |  | **This study** |
|  | HAST 89041211 | Bark | Taiwan | GU300090 | - |  |  | Hsieh et al. (2010) |
|  | CBS 126415 | - | Germany | KY610394 | KY610463 | KY624287 | KX271257 | Wendt et al. (2018) |
| *Xylaria bambusicola* | WSP 205 | *Bambusa oldhamii* | Taiwan | EF026123 | - | GQ844802 | AY951762 | Hsieh et al. (2010) |
| *Xylaria curta* | HAST 494 | Dead wood | Martinique | GU322444 | - | GQ844831 | GQ495937 | Hsieh et al. (2010) |
| *Xylaria discolour* | HAST 131023 | *Ocotea foetens* | USA | JQ087405 | - | JQ087411 | JQ087414 | Ju et al. (2012) |
| *Xylaria hypoxylon* | CBS 122620 | - | Sweden | KY610407 | KY610495 | KY624231 | KX271279 | Wendt et al. (2018) |
| ***Xylaria multiplex*** | **CMRP4983** | ***Vochysia divergens*** | **Brazil** | **ON325487** | **ON325575** | - |  | **This study** |
|  | JDR 259 | *Hibiscus tiliaceus* | USA | GU300099 | - | GQ844815 | GQ487706 | Hsieh et al. (2010) |
|  | HAST 580 | Dead wood | Martinique | GU300098 | - | GQ844814 | GQ487705 | Hsieh et al. (2010) |
| *Xylaria polymorpha* | MUCL 49884 | - | France | KY610408 | KY610464 | KY624288 | KX271280 | Wendt et al. (2018) |
| ***Xylariaceae* sp. 1** | **CMRP4988** | ***Vochysia divergens*** | **Brazil** | **ON325490** | **ON325578** | - |  | **This study** |
| ***Xylariaceae* sp. 1** | **CMRP4992** | ***Vochysia divergens*** | **Brazil** | **ON325491** | **ON325579** | - |  | **This study** |
| ***Xylariaceae* sp. 2** | **CMRP5055** | ***Vochysia divergens*** | **Brazil** | **ON325492** | - | - |  | **This study** |
| ^1^Collection – Type strains included in analysis are indicated with *. Strains marked in bold were those generated by this study.  Culture collections abbreviations: ATCC, American Type Culture Collection, Manassas, USA; BISH, Bishop Museum, Honolulu, USA; CBS = Culture Collection of the Westerdijk Fungal Biodiversity Institute, Utrecht, The Netherlands; CMRP = Microbiological Collections of Paraná Network, Federal University of Paraná, Curitiba, Brazil; GZUH, Guizhou University, Guiyang, China; HAST, Academia Sinica, Taipei, Taiwan; HAST, Academia Sinica, Taipei, Taiwan; MFLUCC, Mae Fah Luang University, Chiang Rai, Thailand; MUCL Agro- food & Environmental Fungal Collection; Y.M.J., Yu-Ming Ju, Academia Sinica, Taipei, Taiwan; WSP, Washington State University, Pullman, USA;  ^2^GenBank - *ITS:* internal transcribed spacers and intervening 5.8S nrDNA; *tub2:* partial beta-tubulin gene; *act:* partial actin gene.  - : Sequence not available | | | | | | | | |

**Table S5.** List of taxa and GenBank accession of the strains generated by this study.

|  |  |  | **GenBank accession no.** | | | | | |
| --- | --- | --- | --- | --- | --- | --- | --- | --- |
| **Identification** | **Collection number** | **Tissue** | **ITS** | **LSU** | ***tef1*** | ***tub2*** | ***his3*** | ***act*** |
| *Alternaria* sp. | CMRP5051 | Leaf | ON325519 | - | - | - | - | - |
| *Aspergillus* sp. | CMRP5218 | Leaf | ON325520 | - | - | - | - | - |
| *Biscogniauxia* sp. | CMRP5023 | Leaf | ON325558 | - | - | - | - | - |
| *Chaetomiaceae* sp. | CMRP5224 | Leaf | ON325564 | - | - | - | - | - |
| *Chaetomiaceae* sp. | CMRP5062 | Leaf | ON325565 | - | - | - | - | - |
| *Chaetomiaceae* sp. | CMRP5063 | Leaf | ON325566 | - | - | - | - | - |
| *Cladosporium* sp. | CMRP5021 | Leaf | ON325543 | - | - | - | - | - |
| *Cladosporium* sp. | CMRP5032 | Petiole | ON325544 | - | - | - | - | - |
| *Cladosporium* sp. | CMRP5226 | Leaf | ON325545 | - | - | - | - | - |
| *Cladosporium* sp. | CMRP5049 | Petiole | ON325546 | - | - | - | - | - |
| *Colletotrichum gigasporum* | CMRP5043 | Petiole | ON325506 | - | - | - | - | - |
| *Colletotrichum* sp. *acutatum* complex | CMRP5221 | Leaf | ON325516 | - | - | - | - | - |
| *Colletotrichum* sp. *acutatum* complex | CMRP5222 | Petiole | ON325517 | - | - | - | - | - |
| *Colletotrichum* sp. *acutatum* complex | CMRP5228 | Leaf | ON325518 | - | - | - | - | - |
| *Colletotrichum* sp. *boninense* complex | CMRP5035 | Petiole | ON325507 | - | - | - | - | - |
| *Colletotrichum* sp. *boninense* complex | CMRP5039 | Leaf | ON325508 | - | - | - | - | - |
| *Colletotrichum* sp. *boninense* complex | CMRP5056 | Leaf | ON325509 | - | - | - | - | - |
| *Colletotrichum* sp. *gloeosporioides* complex | CMRP4974 | Petiole | ON325510 | - | - | - | - | - |
| *Colletotrichum* sp. *gloeosporioides* complex | CMRP4984 | Leaf | ON325511 | - | - | - | - | - |
| *Colletotrichum* sp. *gloeosporioides* complex | CMRP4995 | Leaf | ON325512 | - | - | - | - | - |
| *Colletotrichum* sp. *gloeosporioides* complex | CMRP4999 | Petiole | ON325513 | - | - | - | - | - |
| *Colletotrichum* sp. *gloeosporioides* complex | CMRP5058 | Petiole | ON325514 | - | - | - | - | - |
| *Colletotrichum* sp. *gloeosporioides* complex | CMRP5001 | Leaf | ON325515 | - | - | - | - | - |
| *Corynespora* sp. | CMRP5054 | Petiole | ON325547 | - | - | - | - | - |
| *Curvularia* sp. | CMRP4991 | Leaf | ON325570 | - | - | - | - | - |
| *Curvularia* sp. | CMRP5040 | Leaf | ON325571 | - | - | - | - | - |
| *Daldinia* sp. | CMRP5028 | Petiole | ON325568 | - | - | - | - | - |
| *Diaporthe amolarensis* | CMRP4997 | Petiole | MZ567074 | - | ON564444 | ON675581 | ON755125 | - |
| *Diaporthe cf heveae 1* | CMRP4989 | Leaf | ON325574 | - | ON564442 | - | - | - |
| *Diaporthe cf heveae 1* | CMRP5038 | Petiole | ON325504 | - | ON564443 | - | - | - |
| *Diaporthe infertilis* | CMRP5061 | Petiole | ON325505 | - | ON564446 | - | - | - |
| *Diaporthe podocarpi-macrophylli* | CMRP4990 | Petiole | ON325549 | - | OP056197 | - | - | - |
| *Diaporthe vochysiae* | CMRP4977 | Petiole | ON325493 | - | ON564450 | ON616691 | - | - |
| *Diaporthe vochysiae* | CMRP4978 | Petiole | ON325494 | - | ON564449 | ON616692 | ON788003 | - |
| *Diaporthe vochysiae* | CMRP5220 | Leaf | ON325495 | - | - | - | - | - |
| *Diaporthe vochysiae* | CMRP4994 | Petiole | ON325496 | - | ON564448 | - | - | - |
| *Diaporthe vochysiae* | CMRP4996 | Petiole | ON325497 | - | - | - | - | - |
| *Diaporthe vochysiae* | CMRP5036 | Petiole | ON325498 | - | ON564447 | - | - | - |
| *Diaporthe* sp. | CMRP4985 | Petiole | ON325548 | - | - | - | - | - |
| *Diaporthe* sp. | CMRP5034 | Petiole | ON325550 | - | ON564445 | - | - | - |
| *Endomelanconiopsis* sp. | CMRP5042 | Petiole | ON325551 | - | - | - | - | - |
| *Erythrogloeaceae* sp. | CMRP5022 | Leaf | ON325522 | - | - | - | - | - |
| *Erythrogloeaceae* sp. | CMRP5057 | Leaf | ON325523 | - | - | - | - | - |
| *Fusarium* sp. | CMRP5017 | Petiole | ON325572 | - | - | - | - | - |
| *Fusarium* sp. | CMRP5223 | Petiole | ON325573 | - | - | - | - | - |
| *Hypoxylon* sp. | CMRP5016 | Petiole | ON325556 | - | - | - | - | - |
| *Hypoxylon* sp. | CMRP5053 | Petiole | ON325557 | - | - | - | - | - |
| *Lasiodiplodia pontae* | CMRP4998 | Petiole | ON325499 | - | ON564455 | ON642543 | - | - |
| *Mycosphaerellaceae* sp. | CMRP5024 | Leaf | ON325559 | - | - | - | - | - |
| *Mycosphaerellaceae* sp. | CMRP5025 | Leaf | ON325560 | - | - | - | - | - |
| *Mycosphaerellaceae* sp. | CMRP5026 | Leaf | ON325561 | - | - | - | - | - |
| *Nemania primolutea* | CMRP4987 | Leaf | ON325485 | - | - | ON623731 | - | - |
| *Neopestalotiopsis egyptiaca* | CMRP4981 | Leaf | ON325553 | - | - | ON715002 | - | - |
| *Neopestalotiopsis* sp. | CMRP5000 | Petiole | ON325552 | - | - | - | - | - |
| *Neurospora sublineolata* | CMRP5029 | Petiole | ON325526 | - | - | - | - | - |
| *Neurospora* sp. | CMRP5018 | Petiole | ON325524 | - | - | - | - | - |
| *Neurospora* sp. | CMRP5019 | Petiole | ON325525 | - | - | - | - | - |
| *Nigrospora brasiliensis* | CMRP4975 | Petiole | ON325534 | - | - | - | - | - |
| *Nigrospora* sp. | CMRP4973 | Petiole | ON325527 | - | - | - | - | - |
| *Nigrospora* sp. | CMRP4979 | Petiole | ON325528 | - | - | - | - | - |
| *Nigrospora* sp. | CMRP4980 | Petiole | ON325529 | - | - | - | - | - |
| *Nigrospora* sp. | CMRP5041 | Leaf | ON325530 | - | - | - | - | - |
| *Nigrospora* sp. | CMRP5044 | Leaf | ON325531 | - | - | - | - | - |
| *Nigrospora* sp. | CMRP5045 | Petiole | ON325532 | - | - | - | - | - |
| *Nigrospora* sp. | CMRP5046 | Petiole | ON325533 | - | - | - | - | - |
| *Onygenales* sp. | CMRP5030 | Leaf | ON325563 |  |  |  |  |  |
| *Onygenales* sp. | CMRP5064 | Leaf | ON325567 |  |  |  |  |  |
| *Paecilomyces* sp. | CMRP5052 | Petiole | ON325535 | - | - | - | - | - |
| *Penicillium* sp. sect. *Citrina* | CMRP5227 | Leaf | ON325521 | - | - | - | - | - |
| *Periconia* sp. | CMRP5033 | Petiole | ON325536 | - | - | - | - | - |
| *Pestalotiopsis* sp. | CMRP5037 | Leaf | ON325569 | - | - | - | - | - |
| *Phyllosticta capitalensis* | CMRP4971 | Leaf | ON325540 | - | ON564451 | - | - | ON362211 |
| *Phyllosticta capitalensis* | CMRP4972 | Leaf | ON325541 | - | ON564452 | - | - | ON362212 |
| *Phyllosticta capitalensis* | CMRP5048 | Leaf | ON325542 | - | ON564453 | - | - | ON362213 |
| *Polyscytalum* sp. | CMRP5225 | Leaf | ON325537 | - | - | - | - | - |
| *Prosopidicola* sp. | CMRP5031 | Leaf | ON325554 | - | - | - | - | - |
| *Pseudocercospora* sp. | CMRP5027 | Leaf | ON325538 | - | - | - | - | - |
| *Pseudofusicoccum stromaticum* | CMRP4976 | Leaf | ON325500 | - | - | - | - | - |
| *Pseudofusicoccum stromaticum* | CMRP5219 | Leaf | ON325501 | - | - | - | - | - |
| *Pseudofusicoccum stromaticum* | CMRP4982 | Petiole | ON325502 | - | ON564454 | - | - | - |
| *Pseudofusicoccum stromaticum* | CMRP5047 | Leaf | ON325503 | - | - | - | - | - |
| *Pseudopestalotiopsis* sp. | CMRP5060 | Leaf | ON325555 | - | - | - | - | - |
| *Talaromyces* sp. sect. *Talaromyces* | CMRP5020 | Petiole | ON325539 | - | - | - | - | - |
| *Xylaria adscendens* | CMRP4993 | Leaf | ON325488 | ON325576 | - | ON814139 | - | - |
| *Xylaria arbuscula* | CMRP5050 | Petiole | ON325489 | ON325577 | - | - | - | - |
| *Xylaria arbuscula* | CMRP5059 | Petiole | ON325486 | - | - | - | - | - |
| *Xylaria multiplex* | CMRP4983 | Petiole | ON325487 | ON325575 | - | ON803634 | - | - |
| *Xylariaceae* sp. | CMRP4988 | Leaf | ON325490 | ON325578 | - | ON881284 | - | - |
| *Xylariaceae* sp. | CMRP4992 | Petiole | ON325491 | ON325579 | - | ON911499 | - | - |
| *Xylariaceae* sp. | CMRP5055 | Leaf | ON325492 | - | - | ON981405 | - | - |
| *Xylomelasma sordida* | CMRP4986 | Leaf | ON325562 | - | - | - | - | - |

**Table S6.** Taxonomic classification of each phenotype of endophytic fungi isolated in this study from *Vochysia divergens.*

|  | Taxa | | | | |  |
| --- | --- | --- | --- | --- | --- | --- |
| **Class** | **Order** | **Family** | **Genus** | **Number of isolates** | **Species** | **Phenotype no.** |
| Dothideomycetes (24%) | Botryosphaeriales | Botryosphaeriaceae | *Lasiodiplodia* | 1 | *Lasiodiplodia pontae* | 65 |
|  |  | Endomelanconiopsidaceae | *Endomelanconiopsis* | 1 | *Endomelanconiopsis* sp. | 62 |
|  |  | Phyllostictaceae | *Phyllosticta* | 50 | *Phyllosticta capitalensis* | 1 ,2, 70 |
|  |  | Pseudofusicoccaceae | *Pseudofusicoccum* | 44 | *Pseudofusicoccum stromaticum* | 8, 9, 16, 68 |
|  | Cladosporiales | Cladosporiaceae | *Cladosporium* | 9 | *Cladosporium* sp. *cladosporioides* complex | 25, 46, 71, 72 |
|  | Mycosphaerellales | Mycosphaerellaceae | - | 5 | Mycosphaerellaceae sp. | 28, 29 |
|  |  |  | *Phaeophleospora* | 2 | *Phaeophleospora vochysiae* | 30 |
|  |  |  | *Pseudocercospora* | 2 | *Pseudocercospora* sp. | 31 |
|  | Pleosporales | Corynesporacaceae | *Corynespora* | 1 | *Corynespora cassicola* | 79 |
|  |  | Periconiaceae | *Periconia* | 2 | *Periconia ignaria* | 49 |
|  |  | Pleosporaceae | *Alternaria* | 1 | *Alternaria* sp. | 75 |
|  |  |  | *Curvularia* | 2 | *Curvularia* sp. | 41, 60 |
| Sordariomycetes (69%) |  | Barbatosphaeriaceae | *Xylomelasma* | 1 | *Xylomelasma sordida* | 32 |
|  | Amphisphaeriales | Pestalotiopsidaceae | *Neopestalotiopsis* | 12 | *Neopestalotiopsis* sp. | 15, 76 |
|  |  |  | *Pestalotiopsis* | 2 | *Pestalotiopsis* sp. | 56 |
|  |  | Sporocadaceae | *Pseudopestalotiopsis* | 1 | *Pseudopestlotiopsis* sp. | 86 |
|  | Diaporthales | Diaporthaceae | *Diaporthe* | 50 | *Diaporthe* sp. | 53 |
|  |  |  |  |  | *Diaporthe amolarensis* | 59 |
|  |  |  |  |  | *Diaporthe cf heveae 1* | 36, 57 |
|  |  |  |  |  | *Diaporthe infertilis* | 87 |
|  |  |  |  |  | *Diaporthe cerradensis* | 23 |
|  |  |  |  |  | *Diaporthe podocarpi-macrophylli* | 37 |
|  |  |  |  |  | *Diaporthe vochysiae* | 11, 12, 18, 50, 52, 55 |
|  |  | Chryphonectriaceae | *Chrysocrypta* | 5 | *Chrysocrypta* sp. | 26 ,82 |
|  |  | Prosopidicolaceae | *Prosopidicola* | 1 | Prosopidicolaceae sp. | 45 |
|  | Glomerellales | Glomerellaceae | *Colletotrichum* | 24 | *Colletotrichum gigasporum* | 63 |
|  |  |  |  |  | *Colletotrichum* sp. *boninense* complex | 54, 58, 81 |
|  |  |  |  |  | *Colletotrichum* sp. *gloeosporioides* complex | 4, 19, 51, 69, 83, 85 |
|  |  |  |  |  | *Colletotrichum* sp. *acutatum* complex | 22, 35, 90 |
|  | Hypocreales | Nectriaceae | *Fusarium* | 7 | *Fusarium* sp. | 10, 39 |
|  | Sordariales | Sordariaceae | *Neurospora* | 4 | *Neurospora sublineolata* | 40 |
|  |  |  |  |  | *Neurospora* sp. | 20, 21 |
|  |  | Chaetomiaceae | - | 5 | *Chaetomiaceae* sp. | 44, 88, 89 |
|  | Trichosphaeriales | Trichosphaeriaceae | *Nigrospora* | 24 | *Nigrospora* sp. | 3, 13, 14, 61, 64, 66, 67 |
|  |  |  |  |  | *Nigrospora brasiliensis* | 5 |
|  | Xylariales | Graphostromataceae | *Biscogniauxia* | 1 | *Biscogniauxia* sp. | 27 |
|  |  | Hypoxylaceae | *Hypoxylon* | 4 | *Hypoxylon* sp. | 7, 78 |
|  |  |  | *Daldinia* | 1 | *Daldinia* sp. | 38 |
|  |  | Phlogicylindriaceae | *Polyscytalum* | 5 | *Polyscytalum* sp. | 48 |
|  |  | Xylariaceae | *Nemania* | 1 | *Nemania primolutea* | 33 |
|  |  |  | *Xylaria* | 6 | *Xylaria adscendens* | 47 |
|  |  |  |  |  | *Xylaria arbuscula* | 74, 84 |
|  |  |  |  |  | *Xylaria multiplex* | 17 |
|  |  |  | - | 3 | *Xylariaceae* sp. | 34, 42, 80 |
| Eurotiomycetes (7%) | Eurotiales | Aspergillaceae | *Aspergillus* | 1 | *Aspergillus* sp. sect. *Nigri*, ser. *Japonici* | 6 |
|  |  |  | *Penicillium* | 1 | *Penicillium* sp. sect. *Citrina* | 73 |
|  |  | Thermoascaceae | *Paecilomyces* | 2 | *Paecilomyces* sp. | 77 |
|  |  | Trichocomaceae | *Talaromyces* | 2 | *Talaromyces* sp. sect. *Talaromyces* | 24 |
|  | Onygenales | Onygenales insetae sedis | - |  | - | 43, 91 |


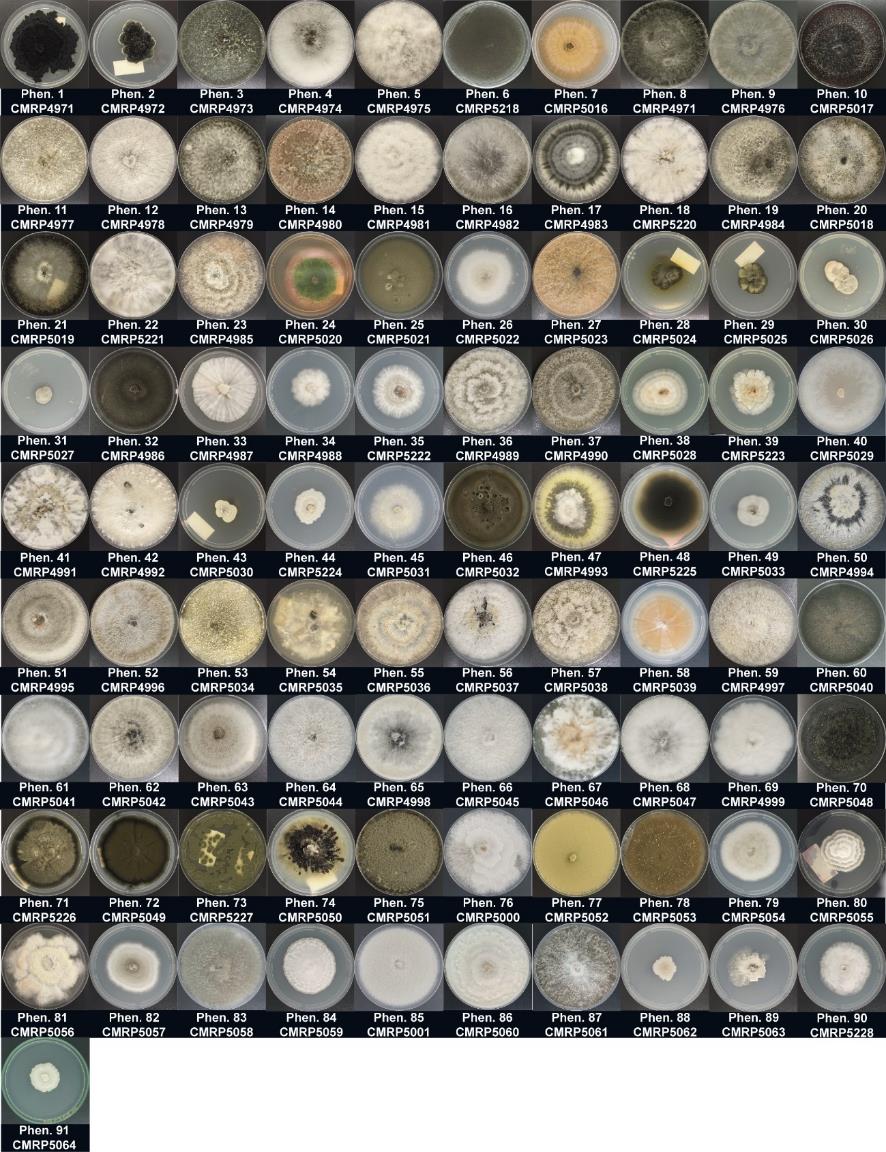


**Figure S1.** Macromorphology of each representative of the 91 phenotypes growth in PDA media after 7-14 days of incubation. The isolates are identified with the phenotype code (Phen.) and the culture collection code (CMRP).


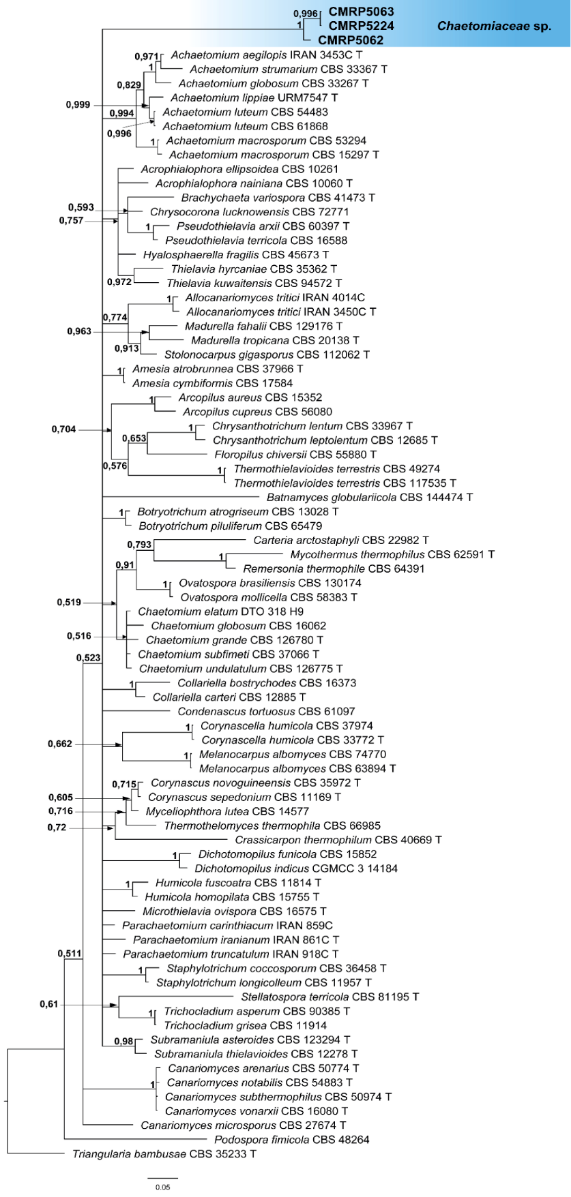


**Figure S2.** Bayesian Inference phylogenetic tree of species from Chaetomiaceae family based on the alignment of ITS partial sequence. The data matrix had 81 taxa and 721 characters. The species *Triangularia bambusae* (CBS 35233) was used as an outgroup. Strains marked with a “T” correspond to type sequences. Bayesian posterior probabilities equal to or greater than 0.50 are presented next to each node in bold. The scale bar of 0.05 represents the number of changes. The sequences of the isolates studied here are presented with its culture collection code (CMRP5224, CMRP5062, and CMRP5063) highlighted in bold.


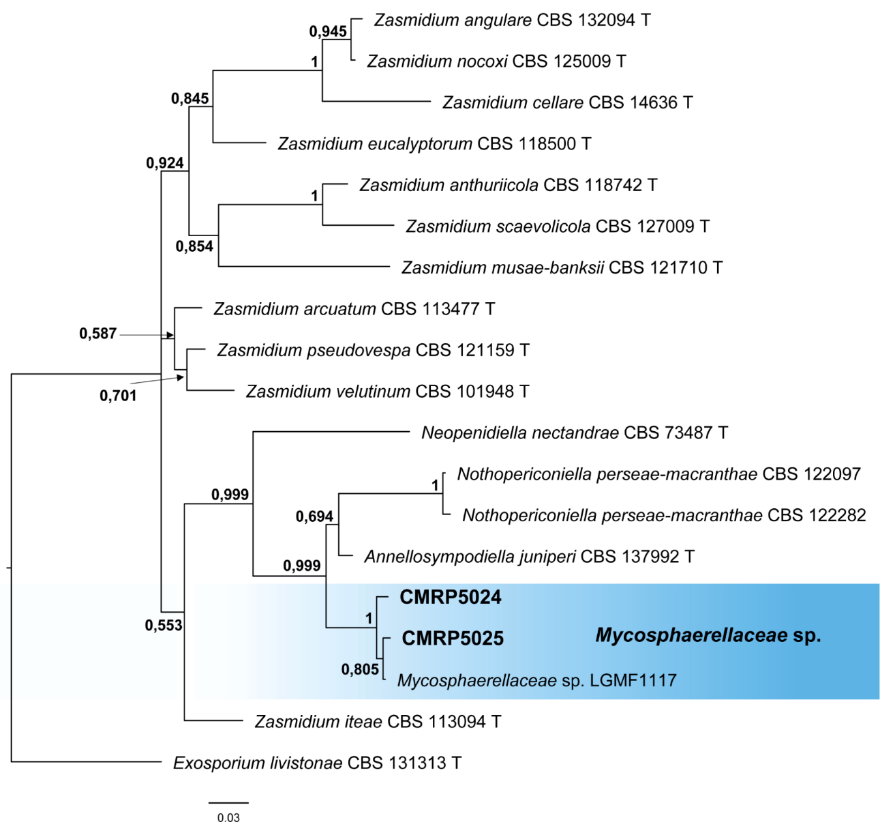


**Figure S3.** Bayesian Inference phylogenetic tree of species from Mycosphaerellaceae family based on the alignment of ITS partial sequence. The data matrix had 19 taxa and 528 characters. The species *Exosporium livistonae* (CBS 131313) was used as an outgroup. Strains marked with a “T” correspond to type sequences. Bayesian posterior probabilities equal to or greater than 0.50 are presented next to each node in bold. The scale bar of 0.03 represents the number of changes. The sequences of the isolates here studied are presented with their culture collection code (CMRP5024 and CMRP5025) highlighted in bold.


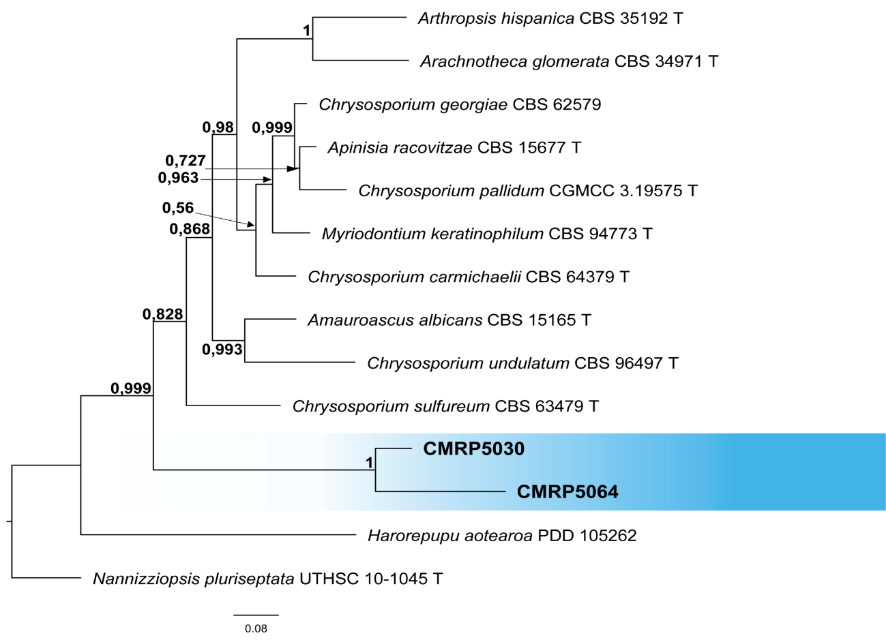


**Figure S4.** Bayesian Inference phylogenetic tree of species from Onygenales *insertae sedis* clade 4 (KANDEMIR et al. 2022) based on the alignment of ITS partial sequence. The data matrix had 14 taxa and 789 characters. The species *Nannizziopsis pluriseptata* (UTHSC 10-1045) was used as an outgroup. Strains marked with a “T” correspond to type sequences. Bayesian posterior probabilities equal to or greater than 0.50 are presented next to each node in bold. The scale bar of 0.08 represents the number of changes. The sequences of the isolates studied here are presented with their culture collection code (CMRP5030 and CMRP5064) highlighted in bold.


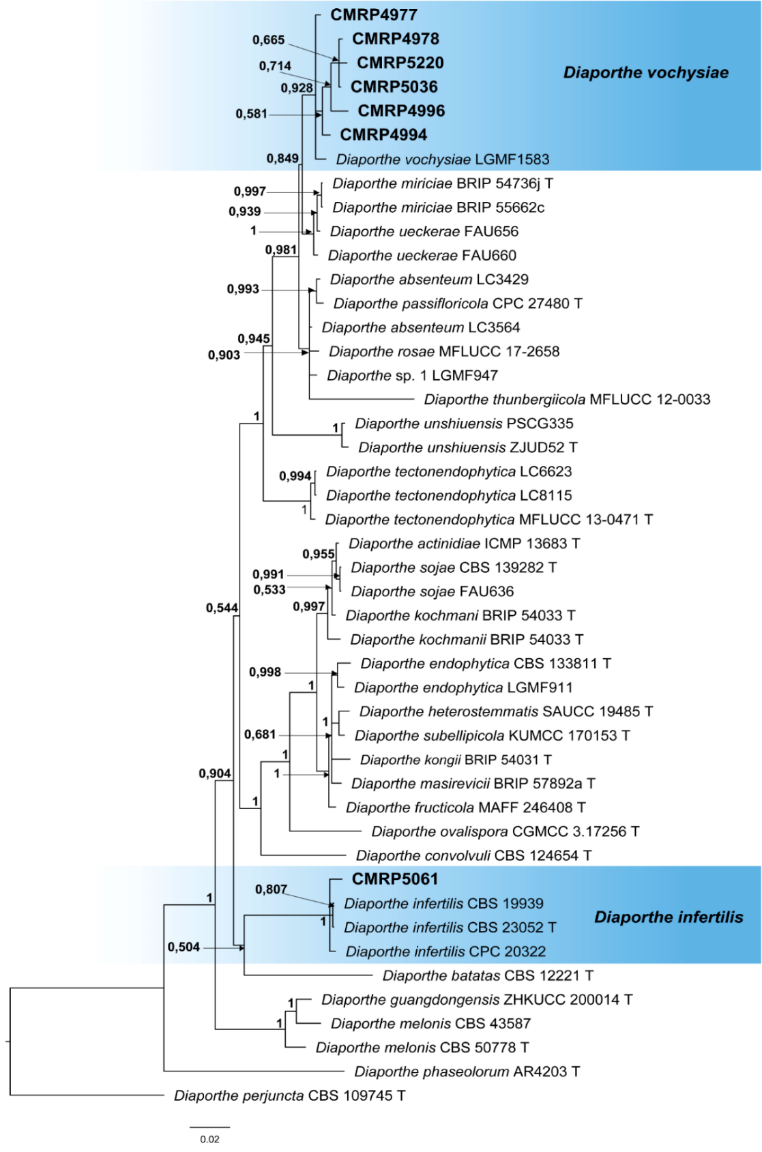


**Figure S5.** Bayesian Inference phylogenetic tree of *Diaporthe sojae* partial species complex based on the multiple alignment of ITS, *tef1*, *tub2* and *his3* partial sequences. The data matrix had 46 taxa and 2273 characters. The species *Diaporthe perjuncta* (CBS 109745) was used as an outgroup. Strains marked with a “T” correspond to type sequences. Bayesian posterior probabilities equal to or greater than 0.50 are presented next to each node in bold. The scale bar of 0.02 represents the number of changes. The sequence of the isolates studied here are presented with its culture collection code (CMRP4977, CMRP4978, CMRP5220, CMRP5036, CMRP4994, CMRP4996, and CMRP5061) highlighted in bold.


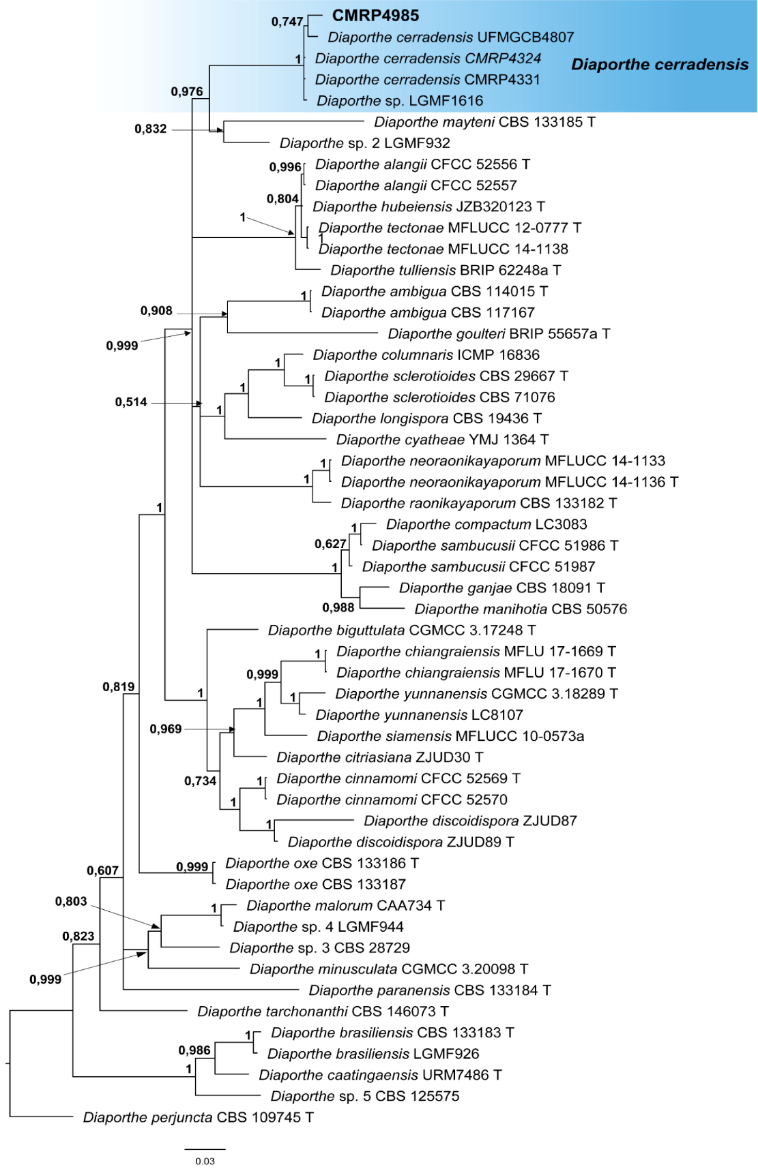


**Figure S6.** Bayesian Inference phylogenetic tree of *Diaporthe sojae* partial species complex based on the alignment of ITS and *tef1* partial sequences. The data matrix had 53 taxa and 1058 characters. The species *Diaporthe perjuncta* (CBS 109745) was used as an outgroup. Strains marked with a “T” correspond to type sequences. Bayesian posterior probabilities equal to or greater than 0.50 are presented next to each node in bold. The scale bar of 0.03 represents the number of changes. The sequence of the isolate studied here is presented with its culture collection code (CMRP4985) highlighted in bold.


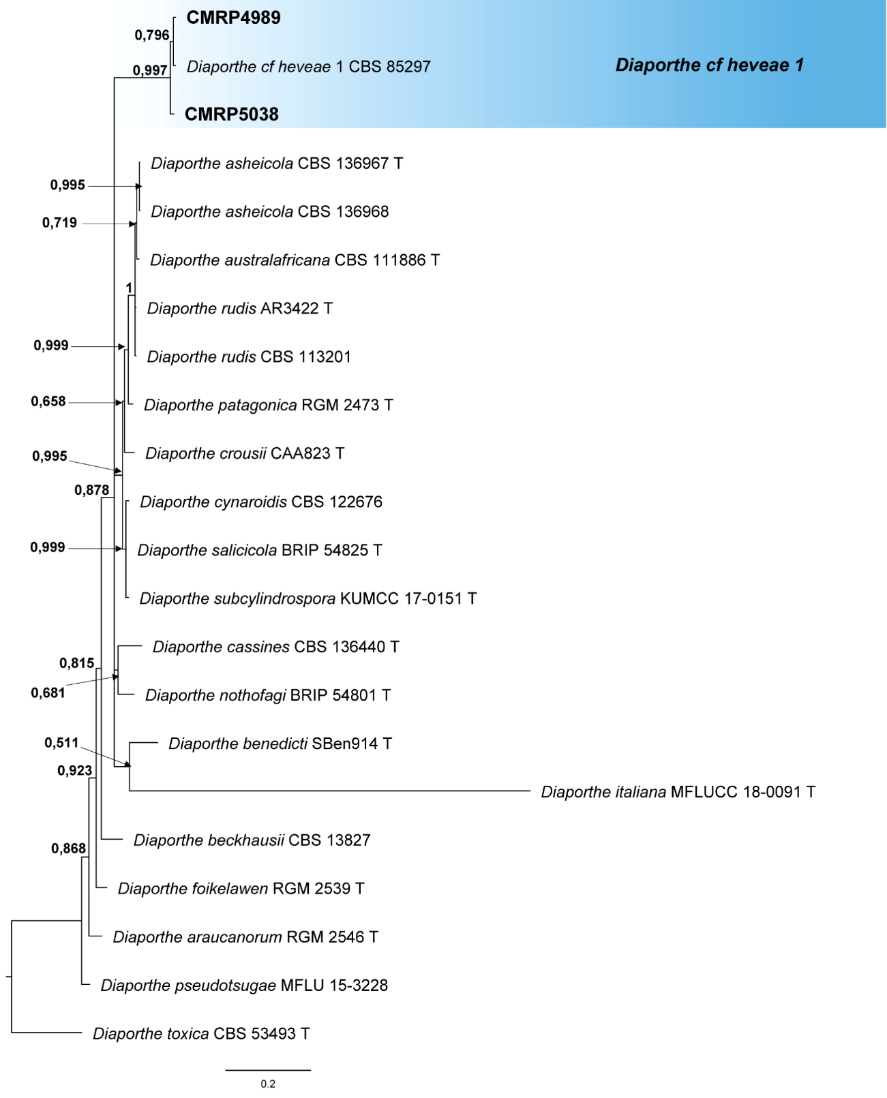


**Figure S7.** Bayesian Inference phylogenetic tree of *Diaporthe rudis* species complex based on the alignment of *tef1* partial sequence. The data matrix had 22 taxa and 635 characters. The species *Diaporthe toxica* (CBS 534.93) was used as an outgroup. Strains marked with a “T” correspond to type sequences. Bayesian posterior probabilities equal to or greater than 0.50 are presented next to each node in bold. The scale bar of 0.2 represents the number of changes. The sequences of the studied isolates are presented with its culture collection code (CMRP4989 and CMRP5038) highlighted in bold.


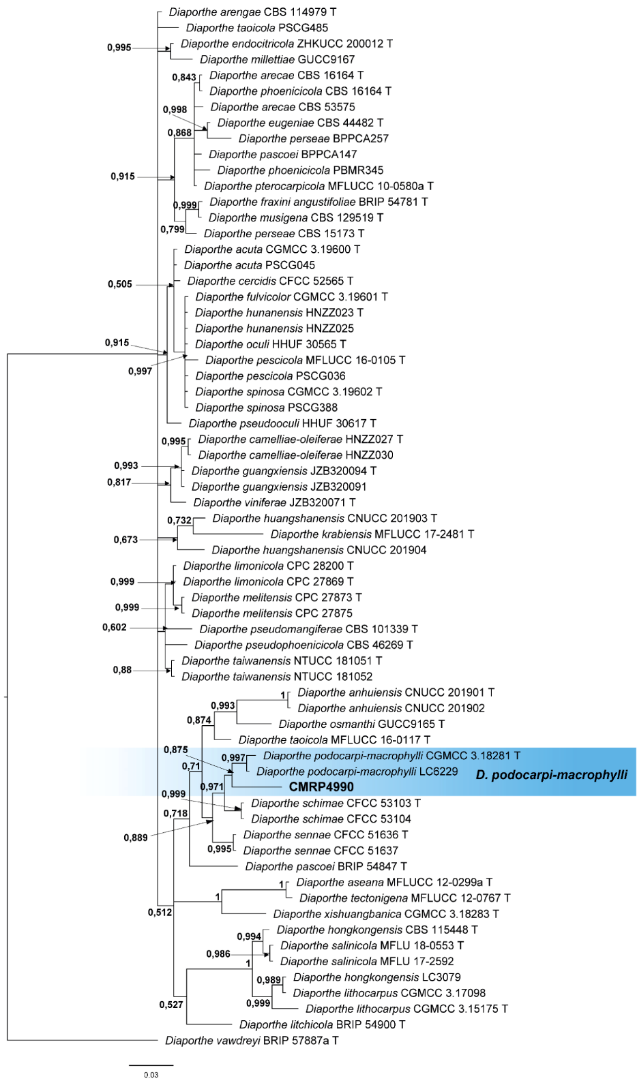


**Figure S8.** Bayesian Inference phylogenetic tree of *Diaporthe arecae* species complex based on the alignment of *tef1* partial sequence. The data matrix had 66 taxa and 385 characters. The species *Diaporthe vawdreyi* (BRIP 57887a) was used as an outgroup. Strains marked with a “T” correspond to type sequences. Bayesian posterior probabilities equal to or greater than 0.50 are presented next to each node in bold. The scale bar of 0.03 represents the number of changes. The sequence of the isolate studied here is presented with its culture collection code (CMRP4990) highlighted in bold.


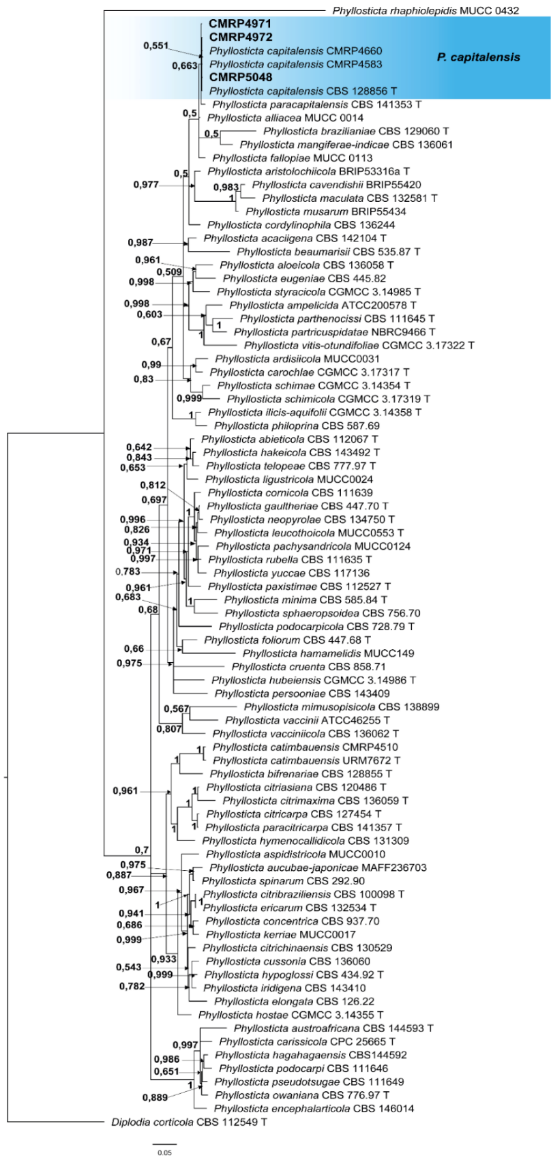


**Figure S9.** Bayesian Inference phylogenetic tree of *Phyllosticta* species based on multilocus alignment of ITS, *tef1*, *act* and *gapdh* partial sequences. The data matrix had 81 taxa and 2038 characters. The species *Diplodia corticola* (CBS 112549) was used as an outgroup. Strains marked with a “T” correspond to type sequences. Bayesian posterior probabilities equal to or greater than 0.50 are presented next to each node in bold. The scale bar of 0.05 represents the number of changes. The sequences of the isolates studied here are presented with their culture collection codes (CMRP4971, CMRP4972, and CMRP5048) highlighted in bold.


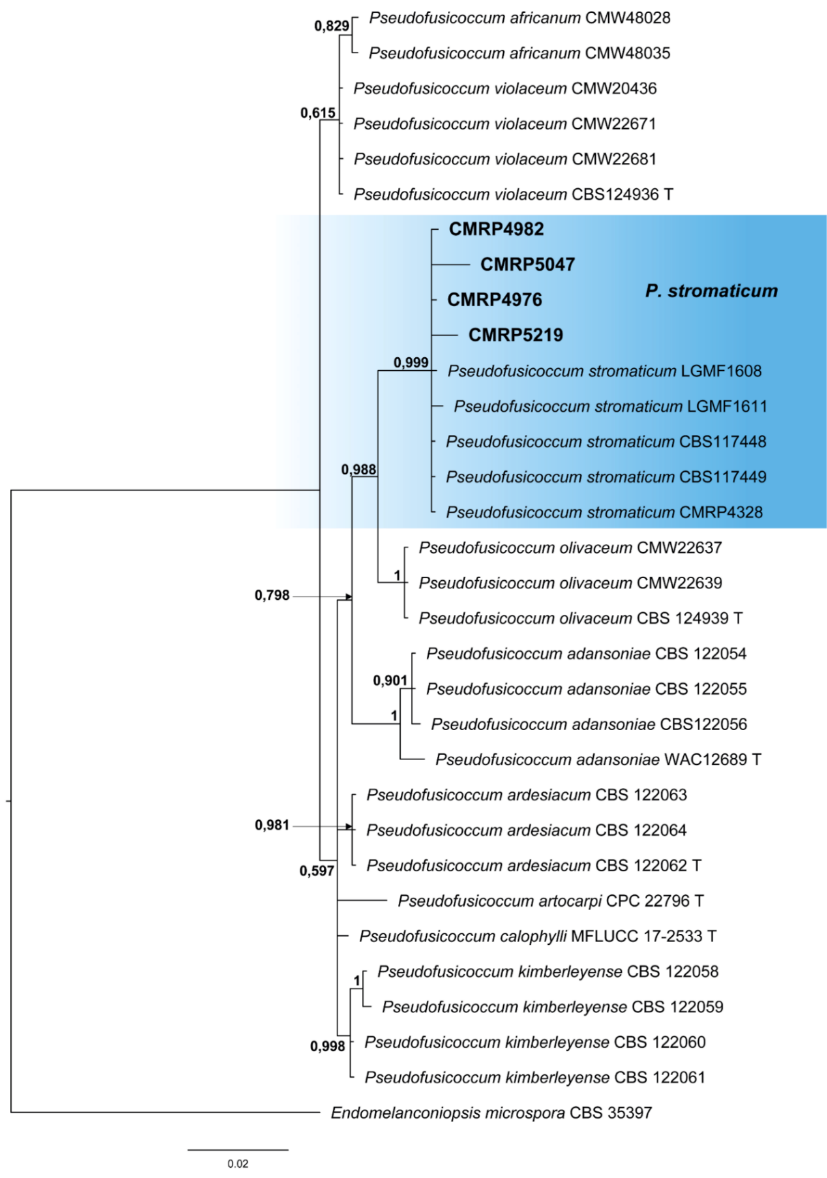


**Figure S10.** Bayesian Inference phylogenetic tree of *Pseudofusicoccum* species based on multiple alignment of ITS and *tef1* partial sequences. The data matrix had 32 taxa and 843 characters. The species *Endomelanconiopsis microspora* (CBS 353.97) was used as an outgroup. Strains marked with a “T” correspond to type sequences. Bayesian posterior probabilities equal to or greater than 0.50 are presented next to each node in bold. The scale bar of 0.02 represents the number of changes. The sequences of the isolates studied here are presented with their culture collection codes (CMRP4982, CMRP5047, CMRP4976, and CMRP5219) highlighted in bold.


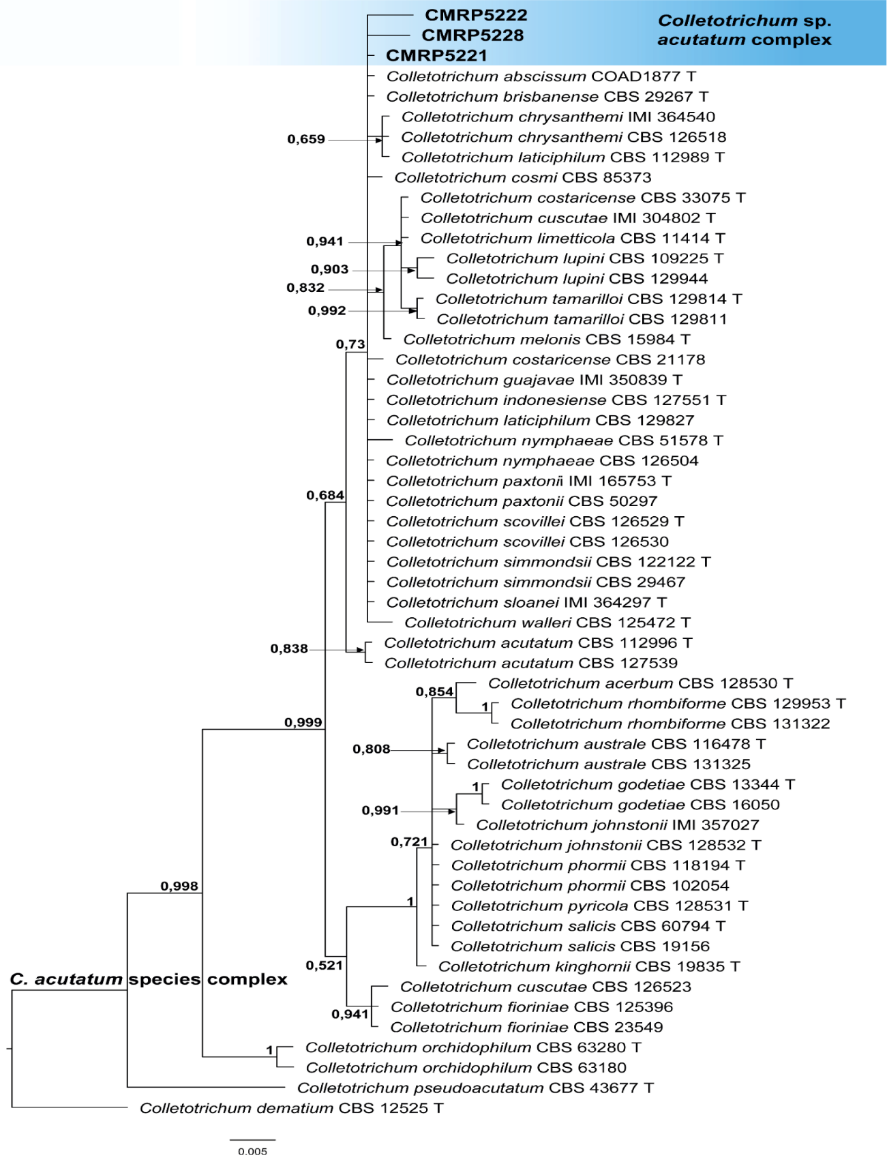


**Figure S11.** Bayesian Inference phylogenetic tree of *Colletotrichum acutatum* species complex based on the alignment of ITS partial sequence. The data matrix had 54 taxa and 556 characters. The species *Colletotrichum dematium* (CBS 125.25) was used as an outgroup. Strains marked with a “T” correspond to type sequences. Bayesian posterior probabilities equal to or greater than 0.50 are presented next to each node in bold. The scale bar of 0.004 represents the number of changes. The sequences of the isolates studied here are presented with their culture collection code (CMRP4974, CMRP5222, CMRP5228, and CMRP5221) highlighted in bold.


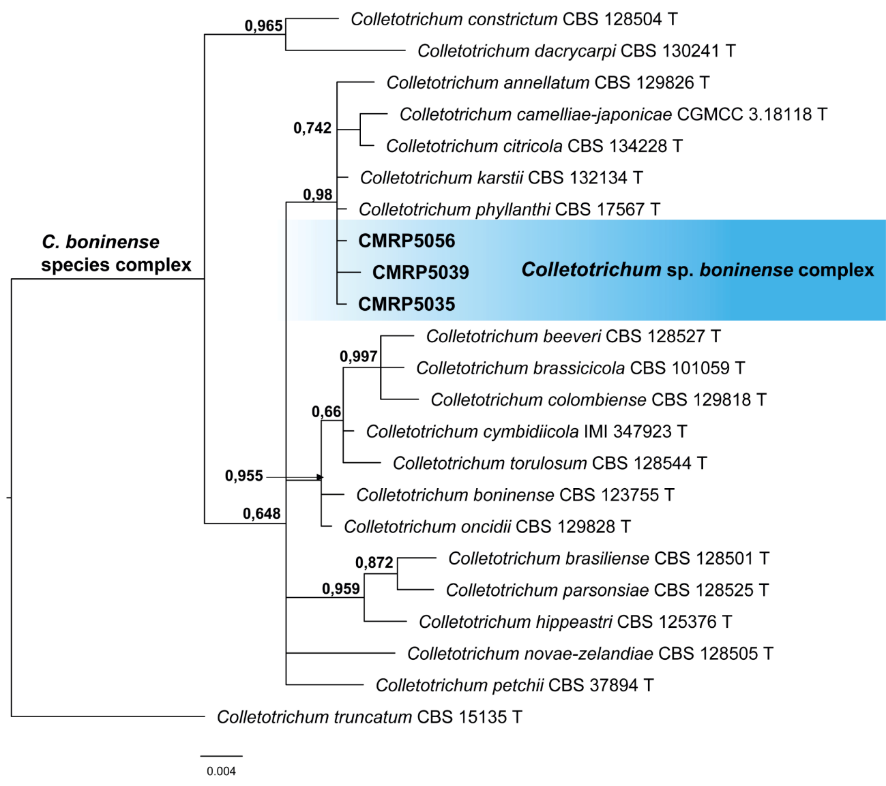


**Figure S12.** Bayesian Inference phylogenetic tree of *Colletotrichum boninense* species complex based on the alignment of ITS partial sequence. The data matrix had 23 taxa and 560 characters. The species *Colletotrichum truncatum* (CBS 151.35) was used as an outgroup. Strains marked with a “T” correspond to type sequences. Bayesian posterior probabilities equal to or greater than 0.50 are presented next to each node in bold. The scale bar of 0.004 represents the number of changes. The sequences of the isolates studied here are presented with their culture collection codes (CMRP5056, CMRP5039, and CMRP5035) highlighted in bold.


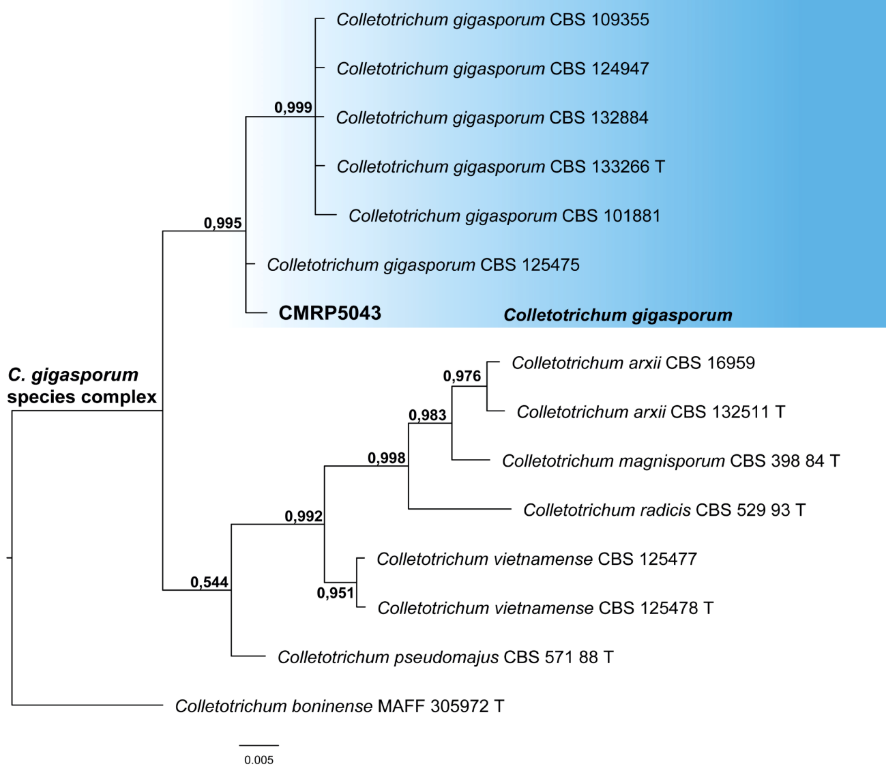


**Figure S13.** Bayesian Inference phylogenetic tree of *Colletotrichum gigasporum* species complex based on the alignment of ITS partial sequence. The data matrix had 16 taxa and 628 characters. The species *Colletotrichum boninense* (MAFF 305972) was used as an outgroup. Strains marked with a “T” correspond to type sequences. Bayesian posterior probabilities equal to or greater than 0.50 are presented next to each node in bold. The scale bar of 0.006 represents the number of changes. The sequence of the isolate here studied is presented with its culture collection code (CMRP5043) highlighted in bold.


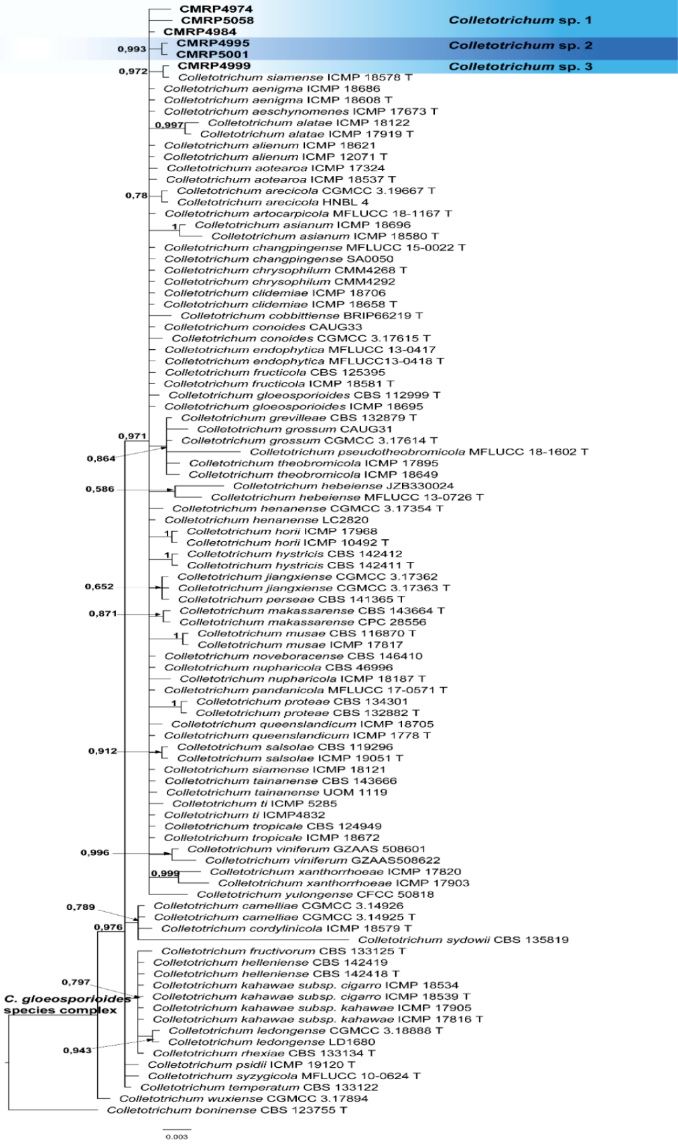


**Figure S14.** Bayesian Inference phylogenetic tree of *Colletotrichum gloeosporioides* species complex based on the alignment of ITS partial sequence. The data matrix had 105 taxa and 634 characters. The species *Colletotrichum boninense* (CBS 123755) was used as an outgroup. Strains marked with a “T” correspond to type sequences. Bayesian posterior probabilities equal or greater than 0.50 are presented next to each node in bold. The scale bar of 0.003 represents the number of changes. The sequences of the isolates here studied are presented with their culture collection codes (CMRP4974, CMRP5058, CMRP4984, CMRP4995, CMRP5001, and CMRP4999) highlighted in bold.


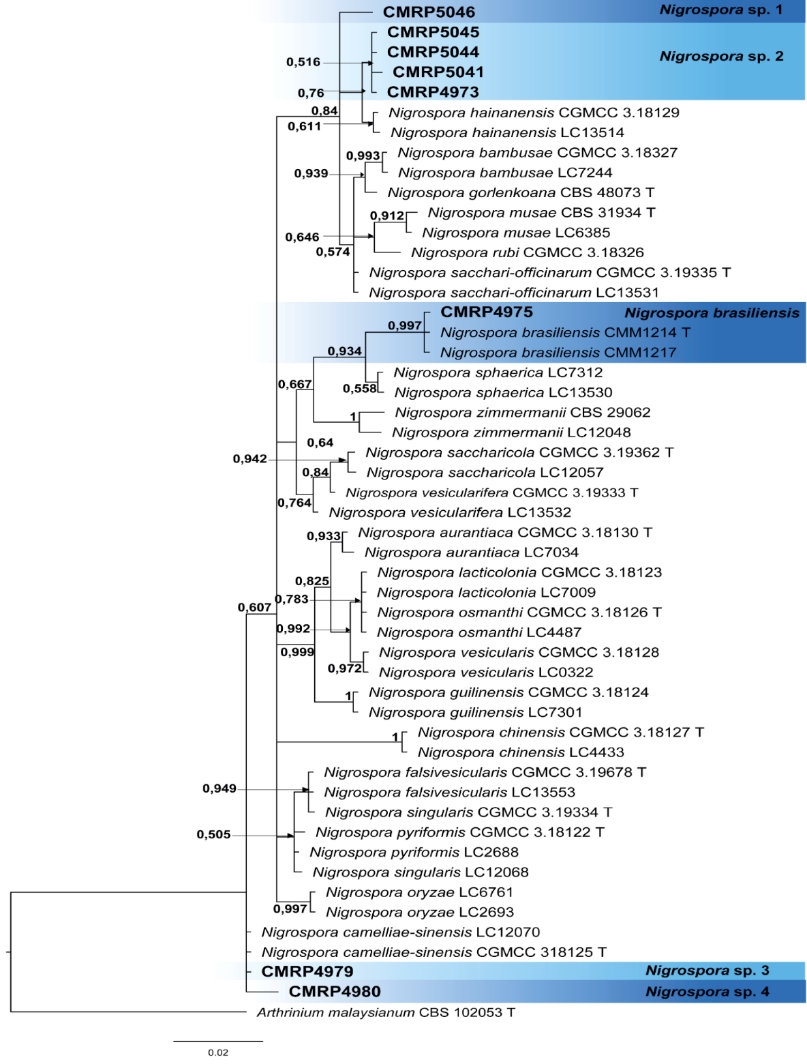


**Figure S15.** Bayesian Inference phylogenetic tree of *Nigrospora* species based on the alignment of ITS partial sequence. The data matrix had 51 taxa and 635 characters. The species *Arthrinium malaysianum* (CBS 102053) was used as an outgroup. Strains marked with a “T” correspond to type sequences. Bayesian posterior probabilities equal to or greater than 0.50 are presented next to each node in bold. The scale bar of 0.02 represents the number of changes. The sequences of the isolates studied here are presented with their culture collection code (CMRP5046, CMRP5045, CMRP5044, CMRP5041, CMRP4973, CMRP4975, CMRP4979, and CMRP4980) highlighted in bold.


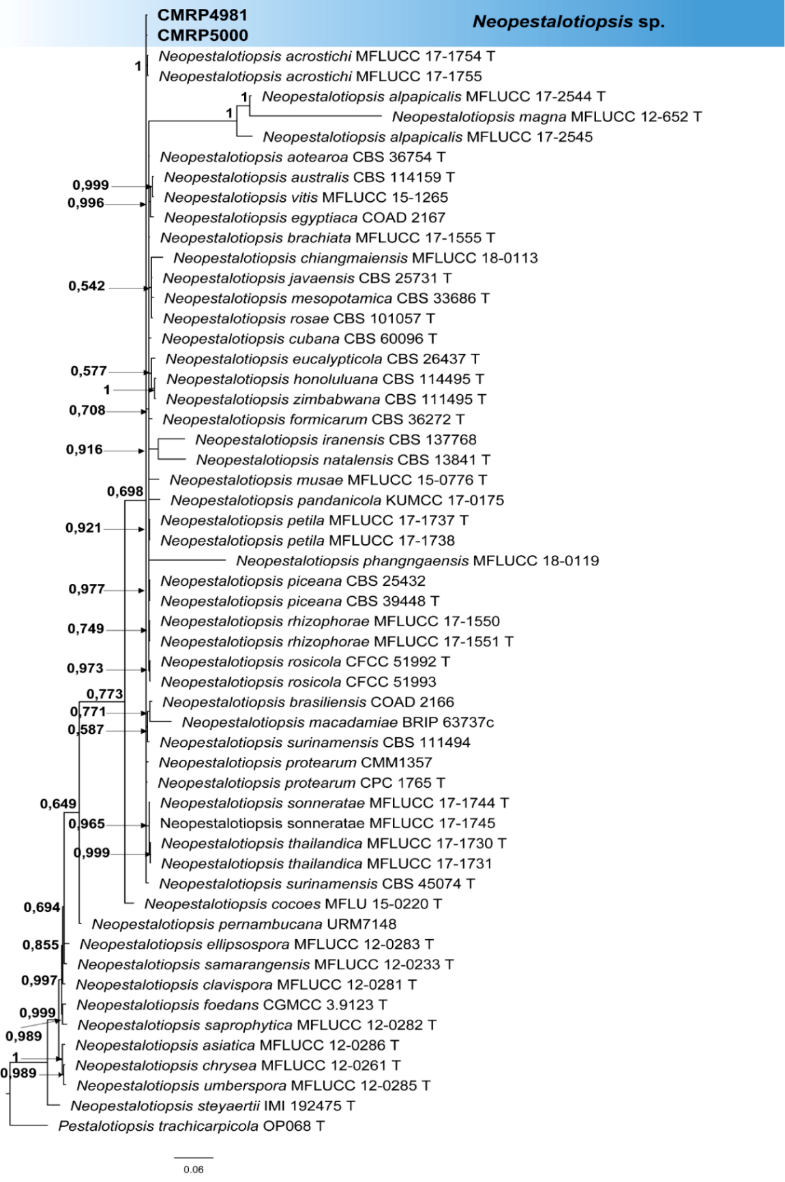


**Figure S16.** Bayesian Inference phylogenetic tree of *Neopestalotiopsis* species based on the multiple alignment of ITS, *tef1* and *tub2* partial sequence. The data matrix had 56 taxa and 2734 characters. The species *Pestalotiopsis trachicarpicola* (OP068) was used as an outgroup. Strains marked with a “T” correspond to type sequences. Bayesian posterior probabilities equal to or greater than 0.50 are presented next to each node in bold. The scale bar of 0.06 represents the number of changes. The sequences of the isolates studied here are presented with their culture collection codes (CMRP4981 and CMRP5000) highlighted in bold.


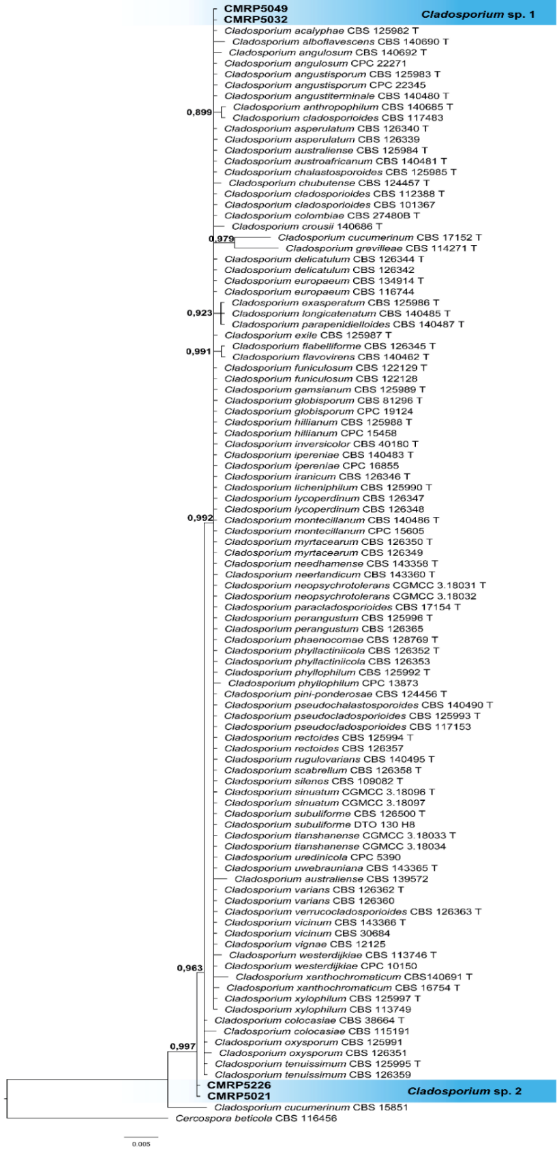


**Figure S17.** Bayesian Inference phylogenetic tree of *Cladosporium* species based on the alignment of ITS partial sequence. The data matrix had 103 taxa and 749 characters. The species *Cercospora beticola* (CBS 116456) was used as an outgroup. Strains marked with a “T” correspond to type sequences. Bayesian posterior probabilities equal to or greater than 0.50 are presented next to each node in bold. The scale bar of 0.005 represents the number of changes. The sequences of the isolates studied here are presented with their culture collection codes (CMRP5049, CMRP5032, CMRP5226, and CMRP5021) highlighted in bold.


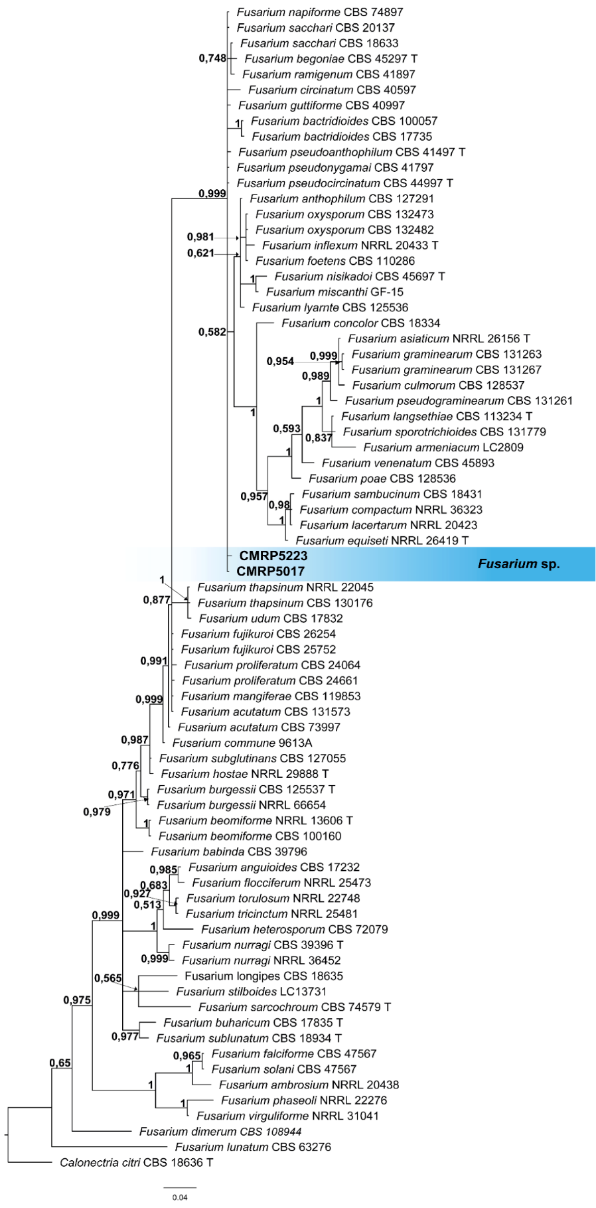


**Figure S18.** Bayesian Inference phylogenetic tree of *Fusarium* species based on the alignment of ITS partial sequence. The data matrix had 75 taxa and 778 characters. The species *Calonectria citri* (CBS 18636) was used as an outgroup. Strains marked with a “T” correspond to type sequences. Bayesian posterior probabilities equal to or greater than 0.50 are presented next to each node in bold. The scale bar of 0.04 represents the number of changes. The sequences of the isolates studied here are presented with their culture collection codes (CMRP5223 and CMRP5017) highlighted in bold.


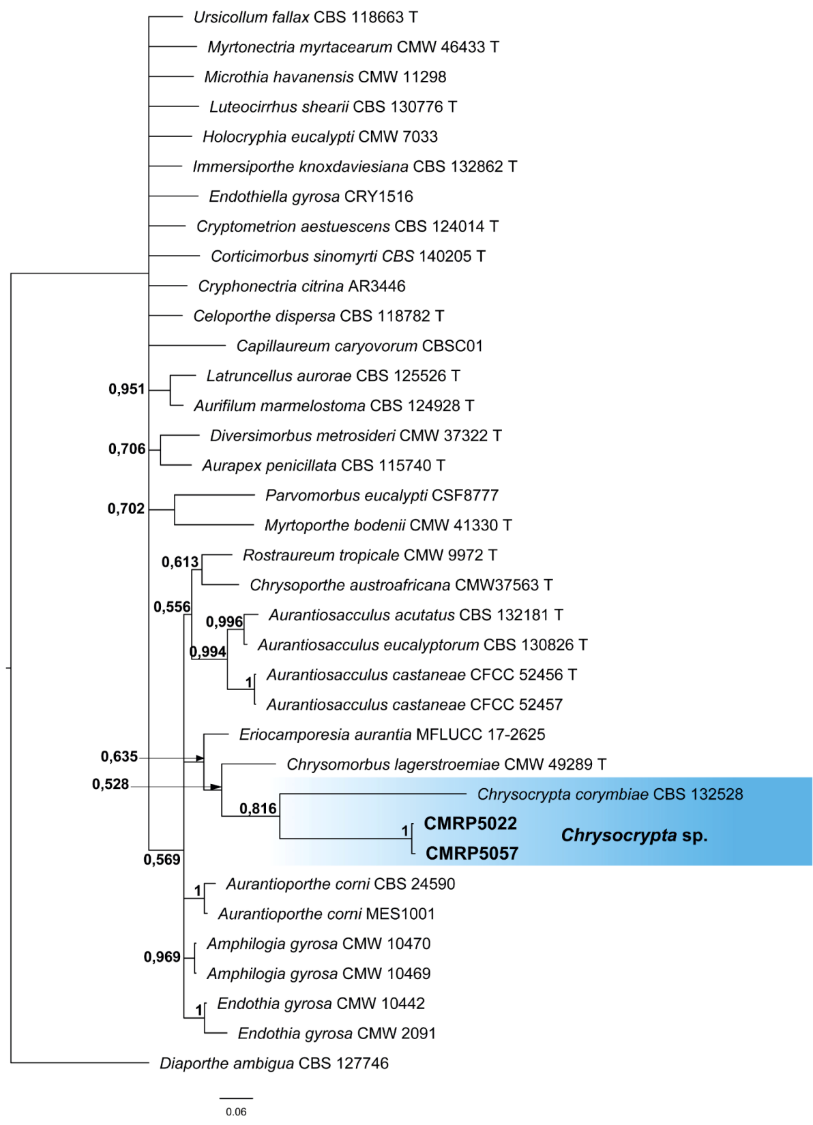


**Figure S19.** Bayesian Inference phylogenetic tree of species from Chryphonectriaceae family based on the alignment of ITS partial sequence. The data matrix had 36 taxa and 620 characters. The species *Diaporthe ambigua* (CBS 127746) was used as an outgroup. Strains marked with a “T” correspond to type sequences. Bayesian posterior probabilities equal to or greater than 0.50 are presented next to each node in bold. The scale bar of 0.06 represents the number of changes. The sequences of the isolates studied here are presented with their culture collection codes (CMRP5022 and CMRP5057) highlighted in bold.


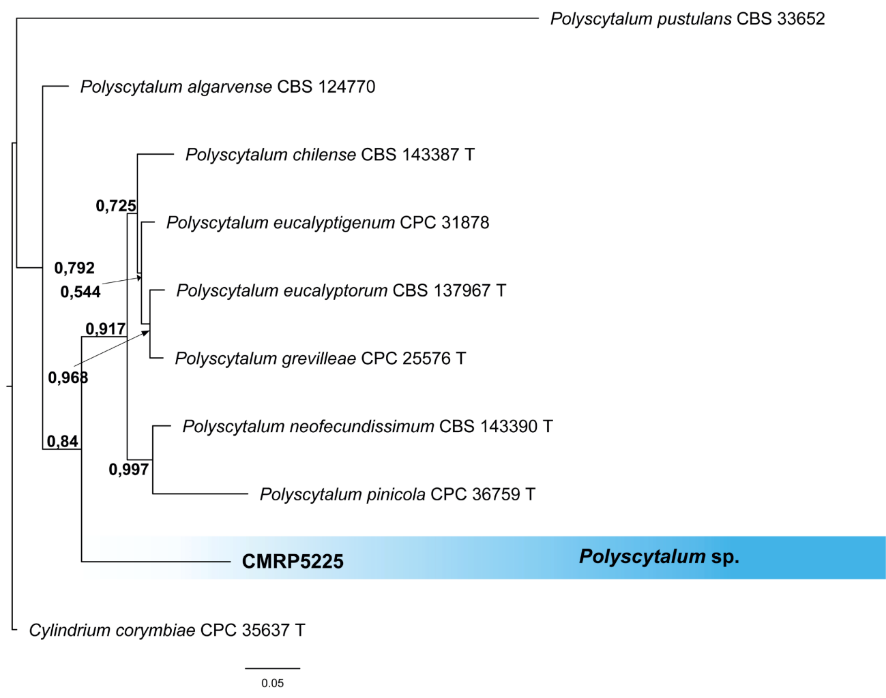


**Figure S20.** Bayesian Inference phylogenetic tree of *Polyscytalum* species based on the alignment of ITS partial sequence. The data matrix had 10 taxa and 957 characters. The species *Cylindrium corymbiae* (CPC 35637) was used as an outgroup. Strains marked with a “T” correspond to type sequences. Bayesian posterior probabilities equal to or greater than 0.50 are presented next to each node in bold. The scale bar of 0.05 represents the number of changes. The sequence of the isolate here studied is presented with its culture collection code (CMRP5225) highlighted in bold.


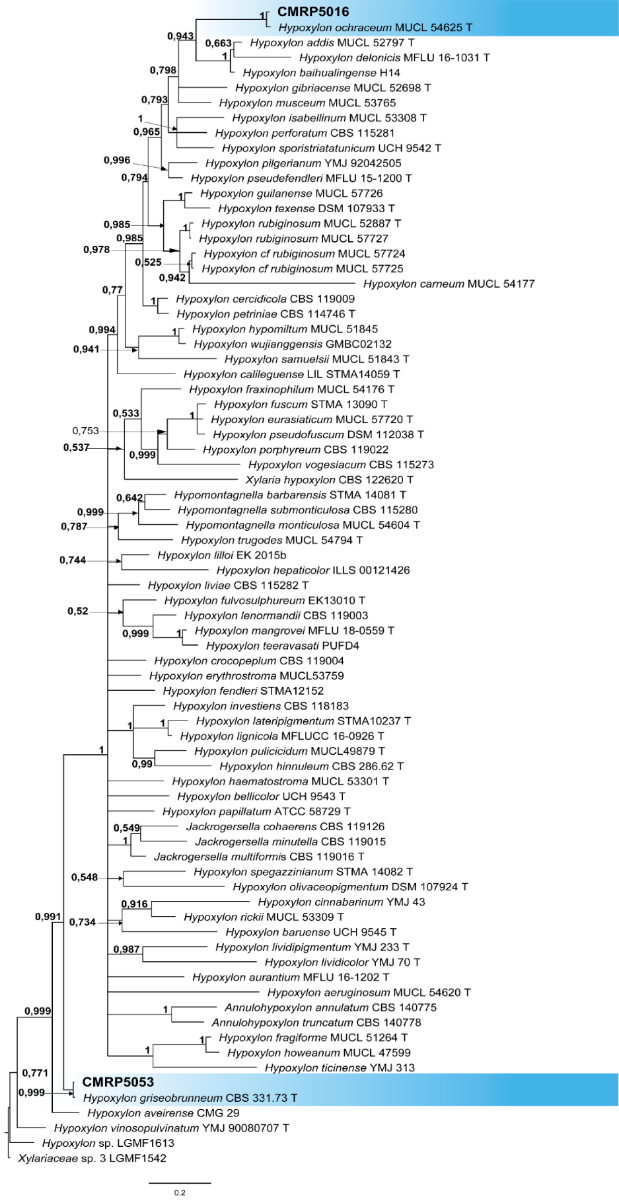


**Figure S21.** Bayesian Inference phylogenetic tree of *Hypoxylon* species based on the alignment of ITS partial sequence. The data matrix had 77 taxa and 956 characters. The species *Xylariaceae* sp. 3 (LGMF 1542) was used as an outgroup. Strains marked with a “T” correspond to type sequences. Bayesian posterior probabilities equal to or greater than 0.50 are presented next to each node in bold. The scale bar of 0.2 represents the number of changes. The sequences of the isolates studied here are presented with their culture collection codes (CMRP5016 and CMRP5053) highlighted in bold.


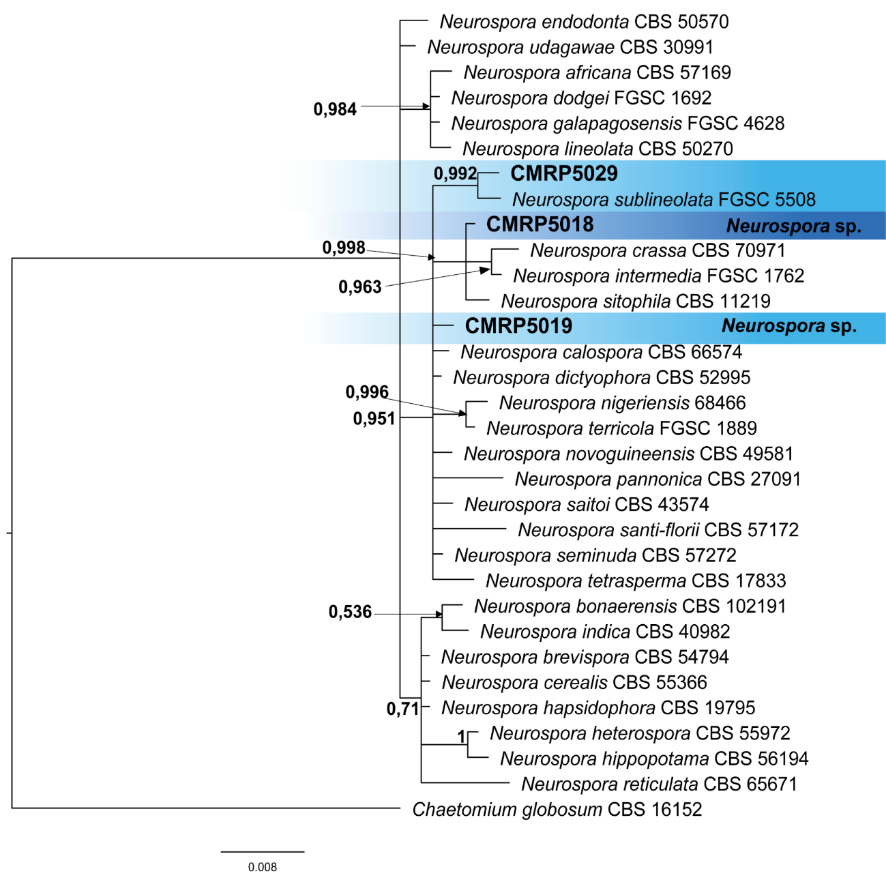


**Figure S22.** Bayesian Inference phylogenetic tree of *Neurospora* species based on the alignment of ITS partial sequence. The data matrix had 32 taxa and 633 characters. The species *Chaetomium globosum* (CBS 161.52) was used as an outgroup. Strains marked with a “T” correspond to type sequences. Bayesian posterior probabilities equal to or greater than 0.50 are presented next to each node in bold. The scale bar of 0.008 represents the number of changes. The sequences of the isolates studied here are presented with their culture collection codes (CMRP5029, CMRP5018, and CMRP5019) highlighted in bold.


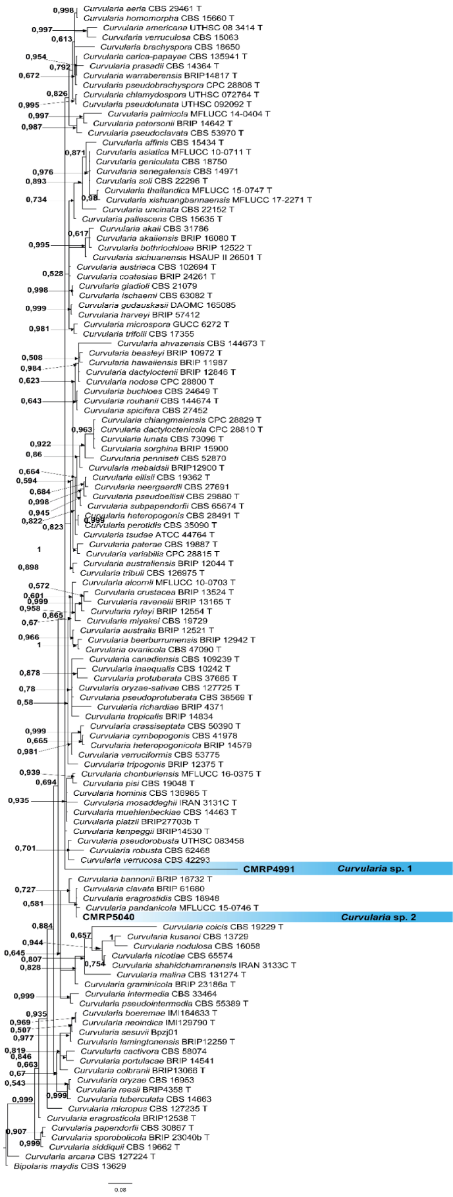


**Figure S23.** Bayesian Inference phylogenetic tree of *Curvularia* species based on the alignment of ITS partial sequence. The data matrix had 122 taxa and 900 characters. The species *Bipolaris maydis* (CBS 136.29) was used as an outgroup. Strains marked with a “T” correspond to type sequences. Bayesian posterior probabilities equal to or greater than 0.50 are presented next to each node in bold. The scale bar of 0.08 represents the number of changes. The sequences of the isolates studied here are presented with their culture collection codes (CMRP4991 and CMRP5040) highlighted in bold.


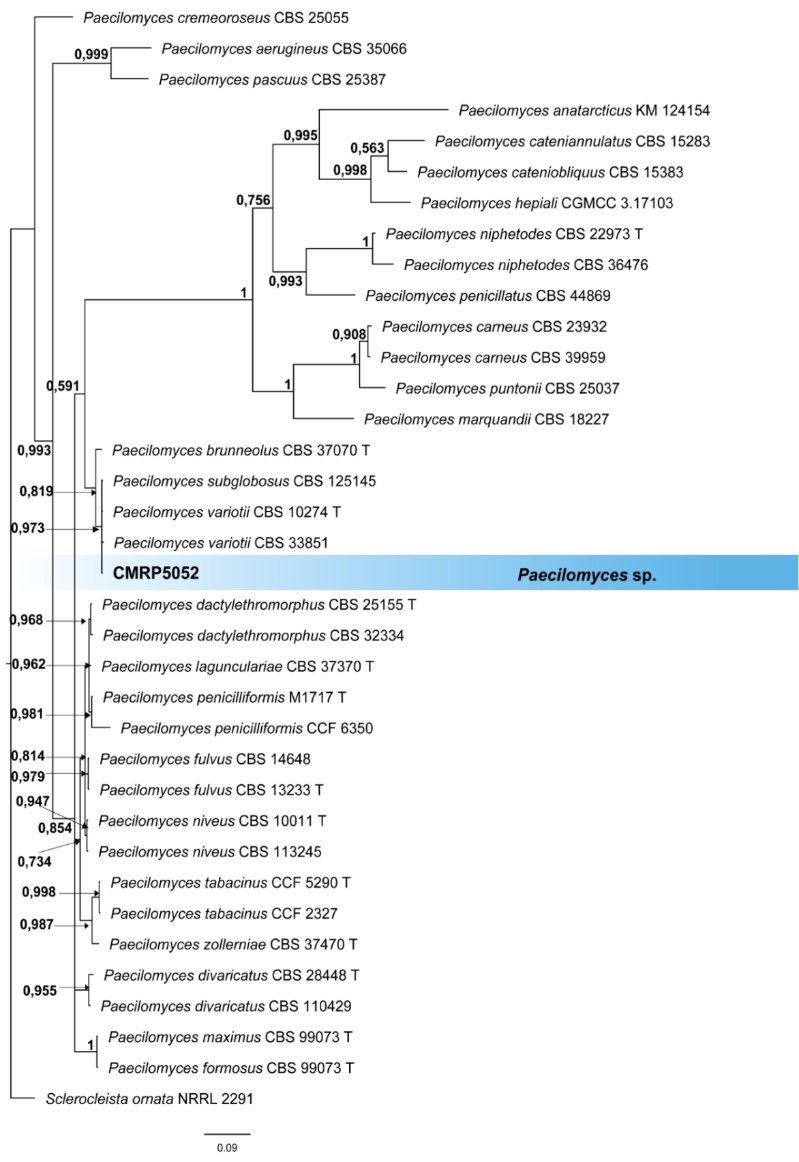


**Figure S24.** Bayesian Inference phylogenetic tree of *Paecilomyces* species based on the alignment of ITS partial sequence. The data matrix had 36 taxa and 770 characters. The species *Sclerocleista ornata* (NRRL 2291) was used as an outgroup. Strains marked with a “T” correspond to type sequences. Bayesian posterior probabilities equal to or greater than 0.50 are presented next to each node in bold. The scale bar of 0.09 represents the number of changes. The sequence of the isolate here studied is presented with its culture collection code (CMRP5052) highlighted in bold.


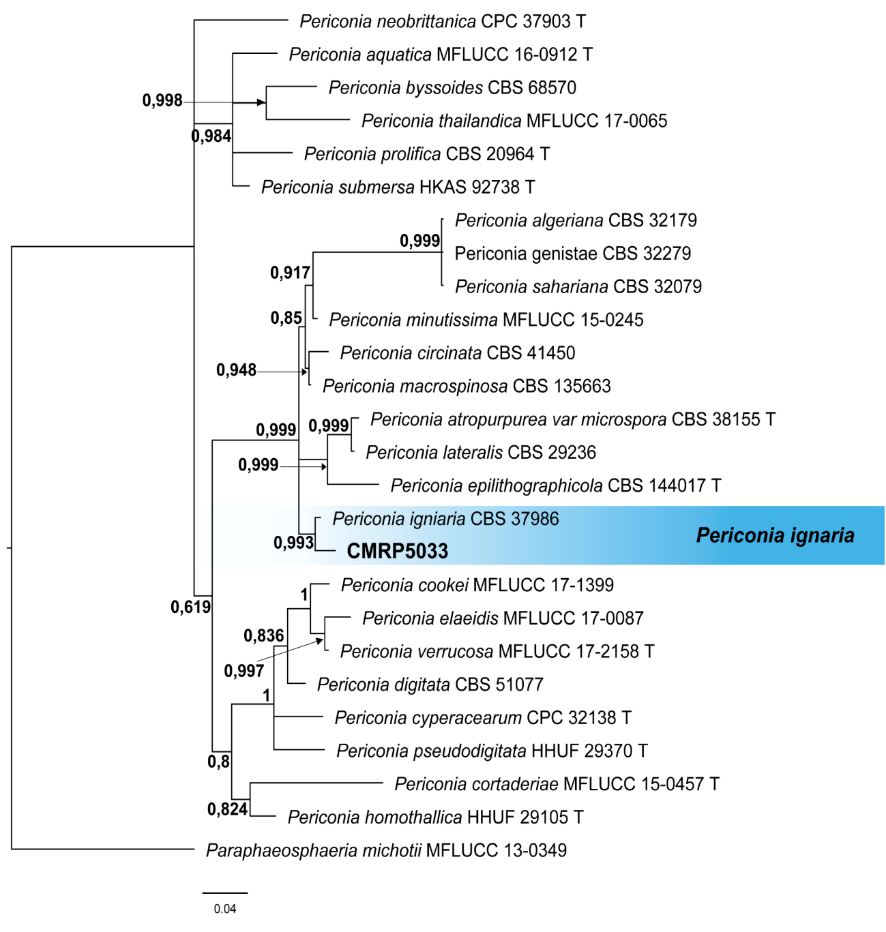


**Figure S25.** Bayesian Inference phylogenetic tree of *Periconia* species based on the alignment of ITS partial sequence. The data matrix had 26 taxa and 1326 characters. The species *Paraphaeosphaeria michotii* (MFLUCC 13-0349) was used as an outgroup. Strains marked with a “T” correspond to type sequences. Bayesian posterior probabilities equal to or greater than 0.50 are presented next to each node in bold. The scale bar of 0.04 represents the number of changes. The sequence of the isolate here studied is presented with its culture collection code (CMRP5033) highlighted in bold.


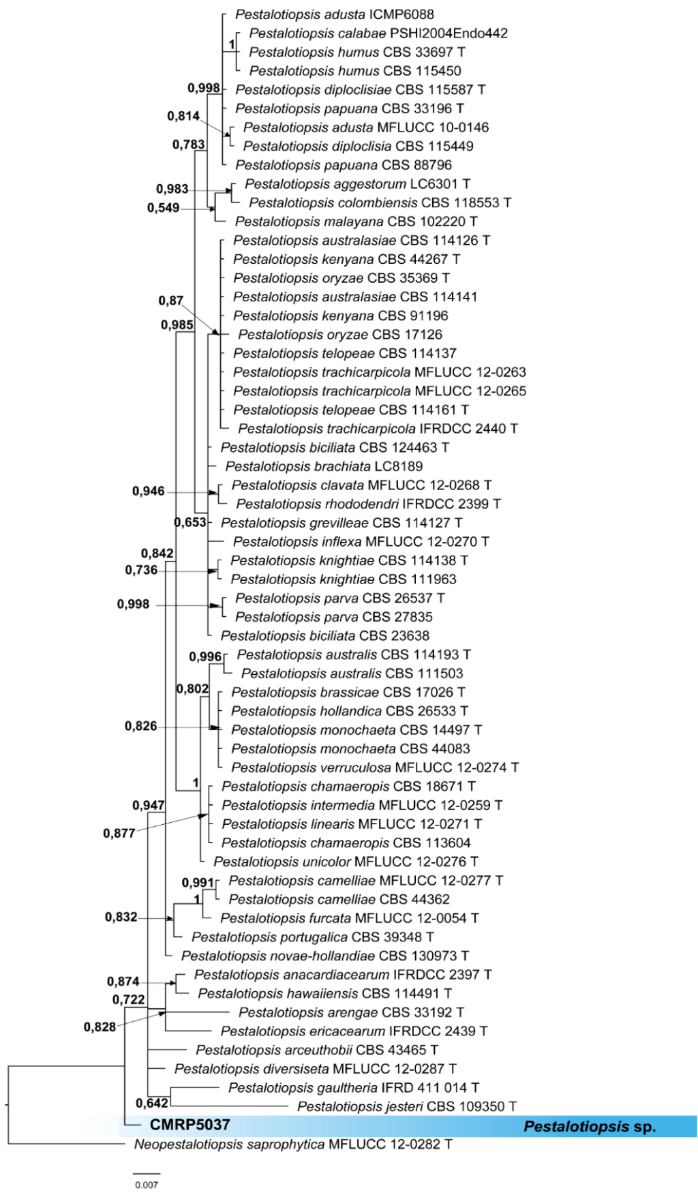


**Figure S26.** Bayesian Inference phylogenetic tree of *Pestalotiopsis* species based on the alignment of ITS partial sequence. The data matrix had 93 taxa and 634 characters. The species *Neopestalotiopsis saprophytica* (MFLUCC 12-0282) was used as an outgroup. Strains marked with a “T” correspond to type sequences. Bayesian posterior probabilities equal to or greater than 0.50 are presented next to each node in bold. The scale bar of 0.007 represents the number of changes. The sequence of the isolate here studied is presented with its culture collection code (CMRP5037) highlighted in bold.


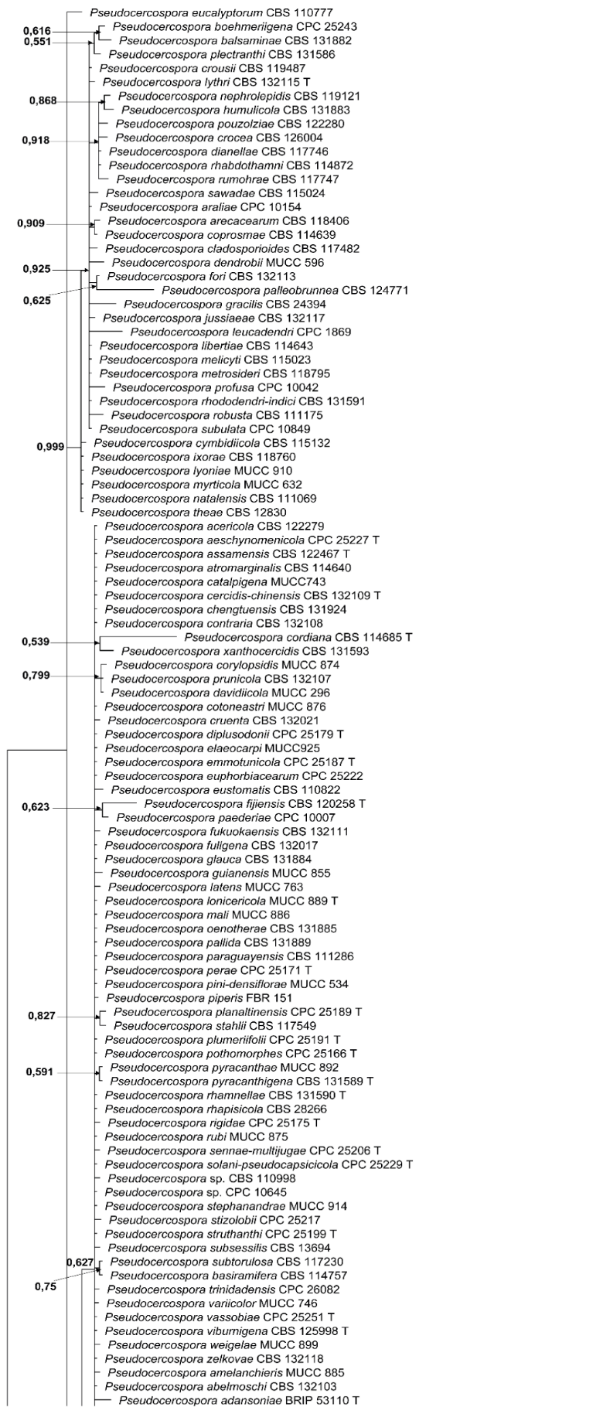


**Figure S27.** (Continue)


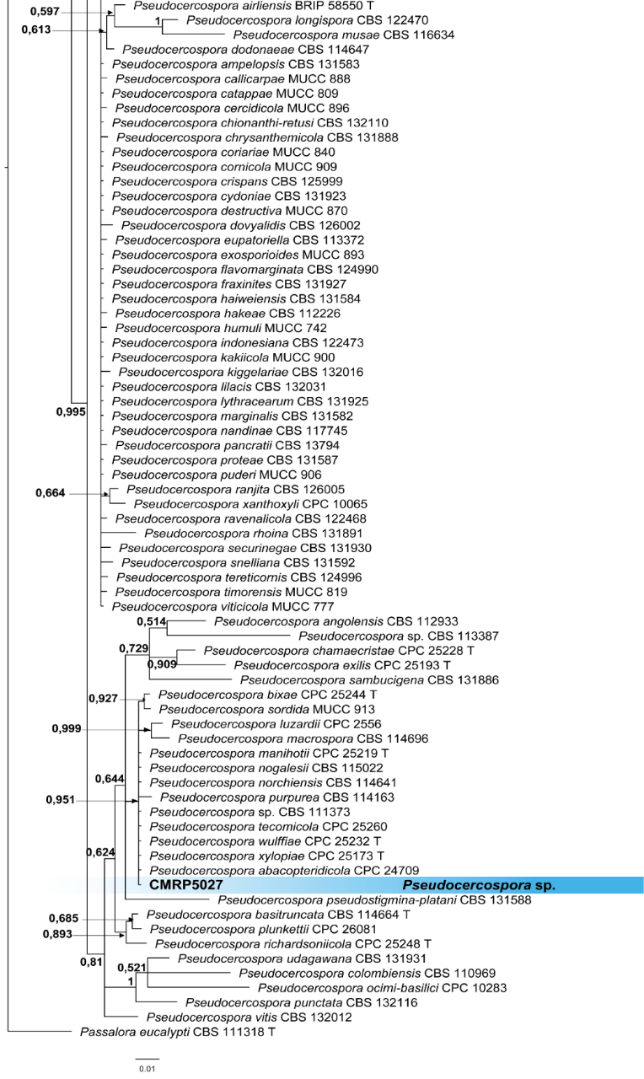


**Figure S27.** Bayesian Inference phylogenetic tree of *Pseudocercospora* species based on the alignment of ITS partial sequence. The data matrix had 172 taxa and 583 characters. The species *Passalora eucalypti* (CBS 111318) was used as an outgroup. Strains marked with a “T” correspond to type sequences. Bayesian posterior probabilities equal to or greater than 0.50 are presented next to each node in bold. The scale bar of 0.01 represents the number of changes. The sequence of the isolate here studied is presented with its culture collection code (CMRP5027) highlighted in bold.


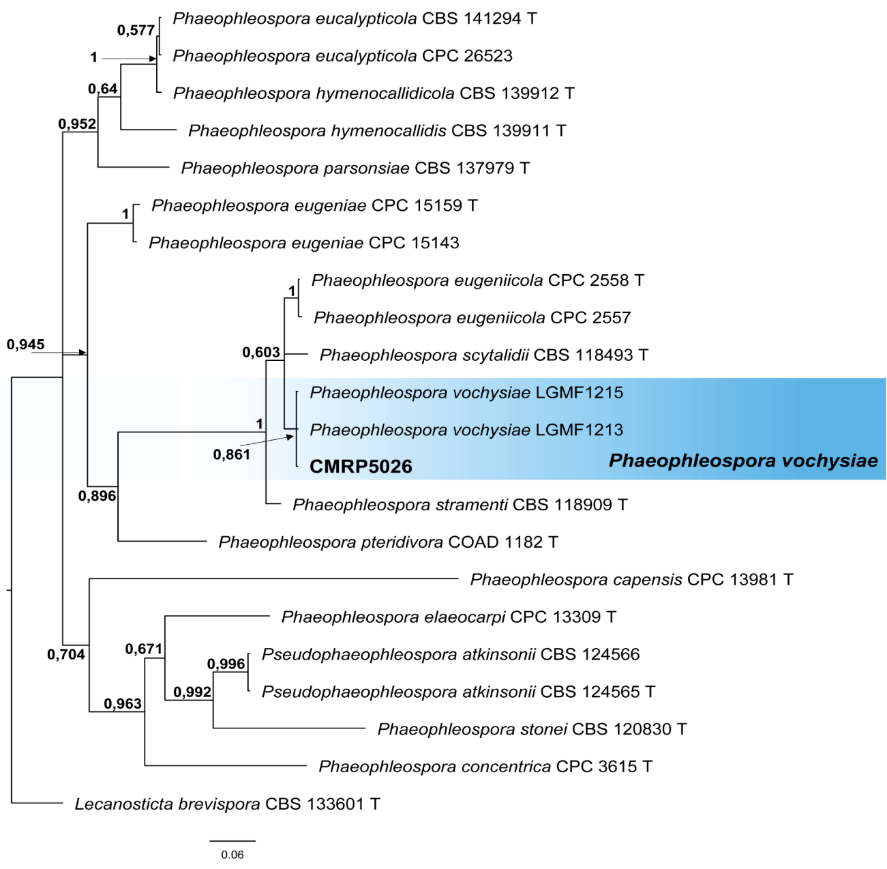


**Figure S28.** Bayesian Inference phylogenetic tree of *Phaeophleospora* species based on the alignment of ITS partial sequence. The data matrix had 22 taxa and 714 characters. The species *Lecanosticta brevispora* (CBS 133601) was used as an outgroup. Strains marked with a “T” correspond to type sequences. Bayesian posterior probabilities equal to or greater than 0.50 are presented next to each node in bold. The scale bar of 0.06 represents the number of changes. The sequence of the isolate here studied is presented with its culture collection code (CMRP5026) highlighted in bold.


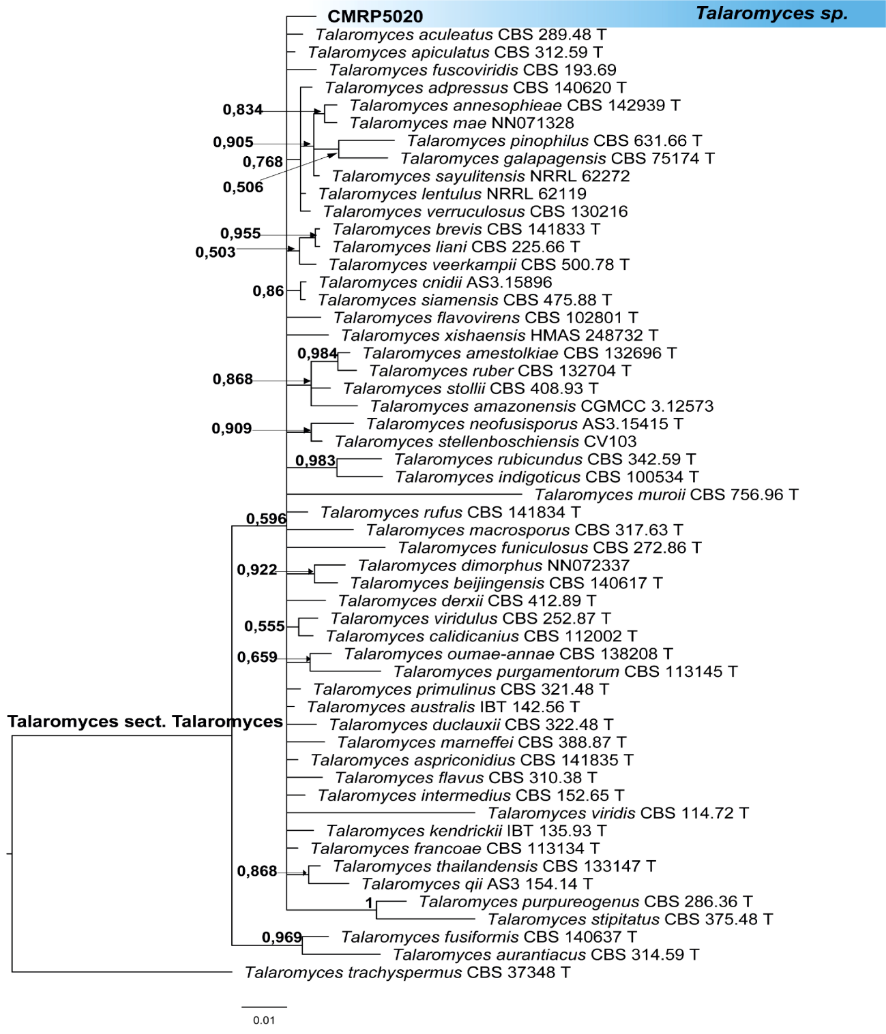


**Figure S29.** Bayesian Inference phylogenetic tree of *Talaromyces* section *Talaromyces* species based on the alignment of ITS partial sequence. The data matrix had 55 taxa and 787 characters. The species *Talaromyces trachyspermus* (CBS 37348) was used as an outgroup. Strains marked with a “T” correspond to type sequences. Bayesian posterior probabilities equal to or greater than 0.50 are presented next to each node in bold. The scale bar of 0.01 represents the number of changes. The sequence of the isolate here studied is presented with its culture collection code (CMRP5020) highlighted in bold.


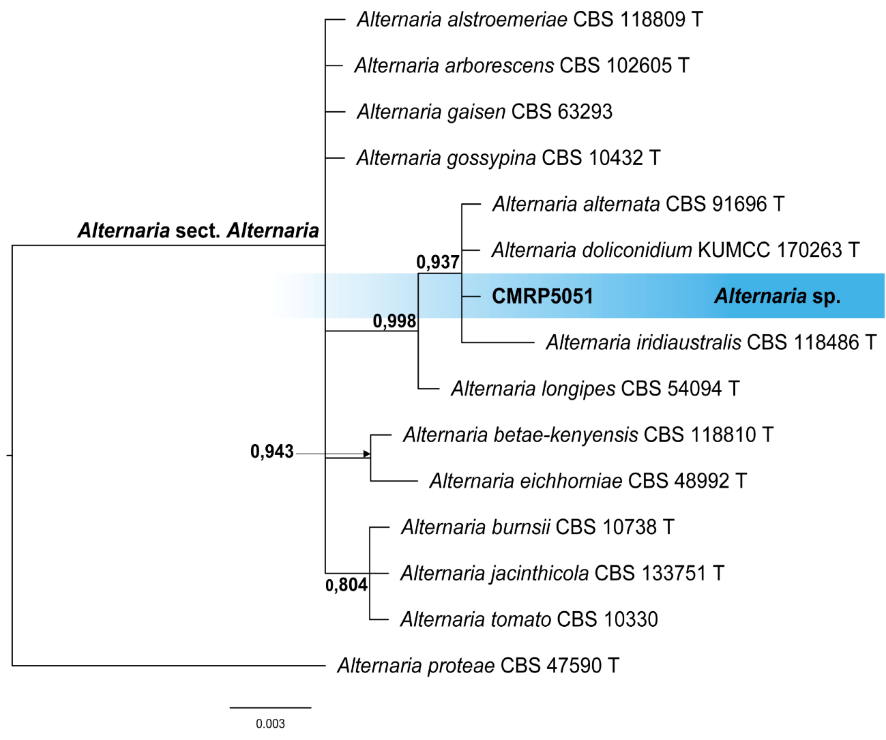


**Figure S30.** Bayesian Inference phylogenetic tree of *Alternaria* section *Alternaria* species based on the alignment of ITS partial sequence. The data matrix had 15 taxa and 570 characters. The species *Alternaria protae* (CBS 47590) was used as an outgroup. Strains marked with a “T” correspond to type sequences. Bayesian posterior probabilities equal to or greater than 0.50 are presented next to each node in bold. The scale bar of 0.003 represents the number of changes. The sequence of the isolate here studied is presented with its culture collection code (CMRP5051) highlighted in bold.


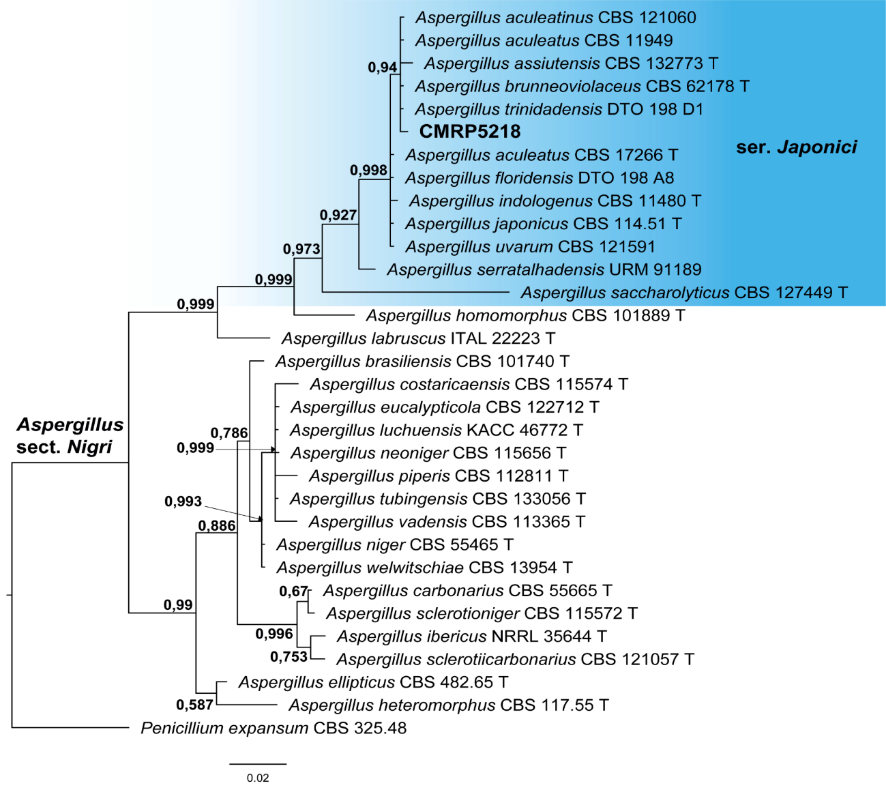


**Figure S31.** Bayesian Inference phylogenetic tree of *Aspergillus* section *Nigri* species based on the alignment of ITS partial sequence. The data matrix had 32 taxa and 641 characters. The species *Penicillium expansum* (CBS 325.48) was used as an outgroup. Strains marked with a “T” correspond to type sequences. Bayesian posterior probabilities equal to or greater than 0.50 are presented next to each node in bold. The scale bar of 0.02 represents the number of changes. The sequence of the isolate here studied is presented with its culture collection code (CMRP5218) highlighted in bold.


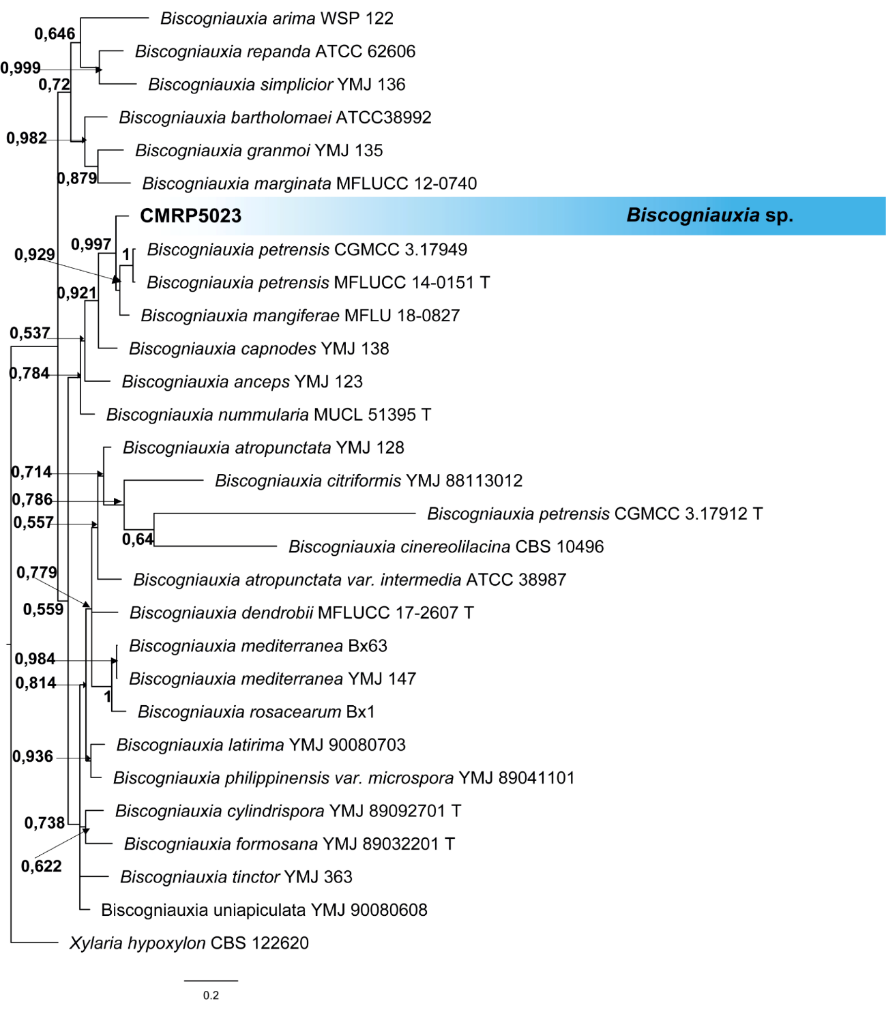


**Figure S32.** Bayesian Inference phylogenetic tree of *Biscogniauxia* species based on the alignment of ITS partial sequence. The data matrix had 29 taxa and 1055 characters. The species *Xylaria hypoxylon* (CBS 122620) was used as an outgroup. Strains marked with a “T” correspond to type sequences. Bayesian posterior probabilities equal to or greater than 0.50 are presented next to each node in bold. The scale bar of 0.2 represents the number of changes. The sequence of the isolate here studied is presented with its culture collection code (CMRP5023) highlighted in bold.


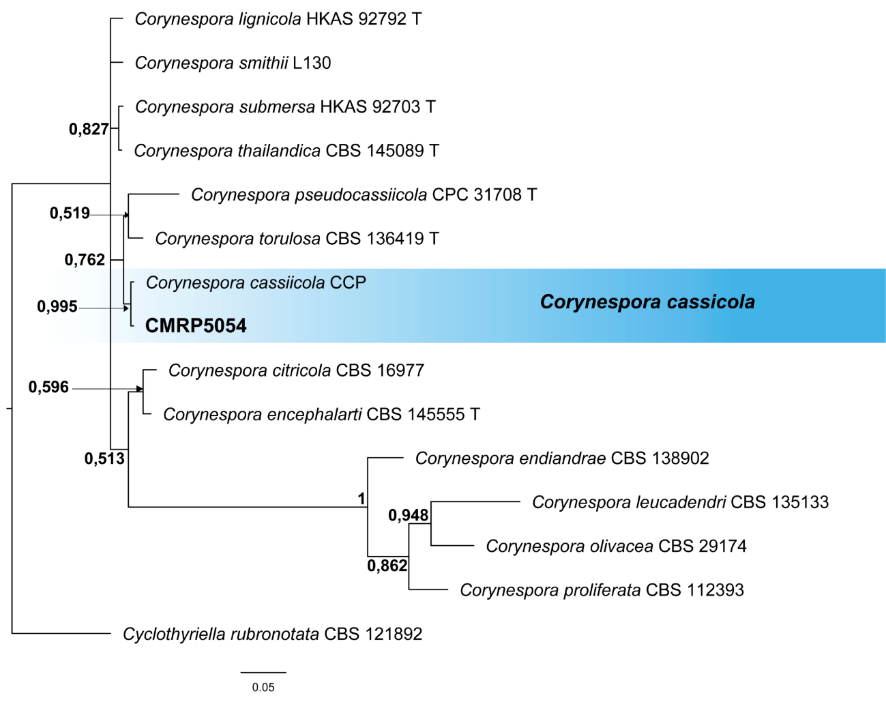


**Figure S33.** Bayesian Inference phylogenetic tree of *Corynespora* species based on the alignment of ITS partial sequence. The data matrix had 15 taxa and 1067 characters. The species *Cyclothyriella rubronotata* (CBS 121892) was used as an outgroup. Strains marked with a “T” correspond to type sequences. Bayesian posterior probabilities equal to or greater than 0.50 are presented next to each node in bold. The scale bar of 0.05 represents the number of changes. The sequence of the isolate here studied is presented with its culture collection code (CMRP5054) highlighted in bold.


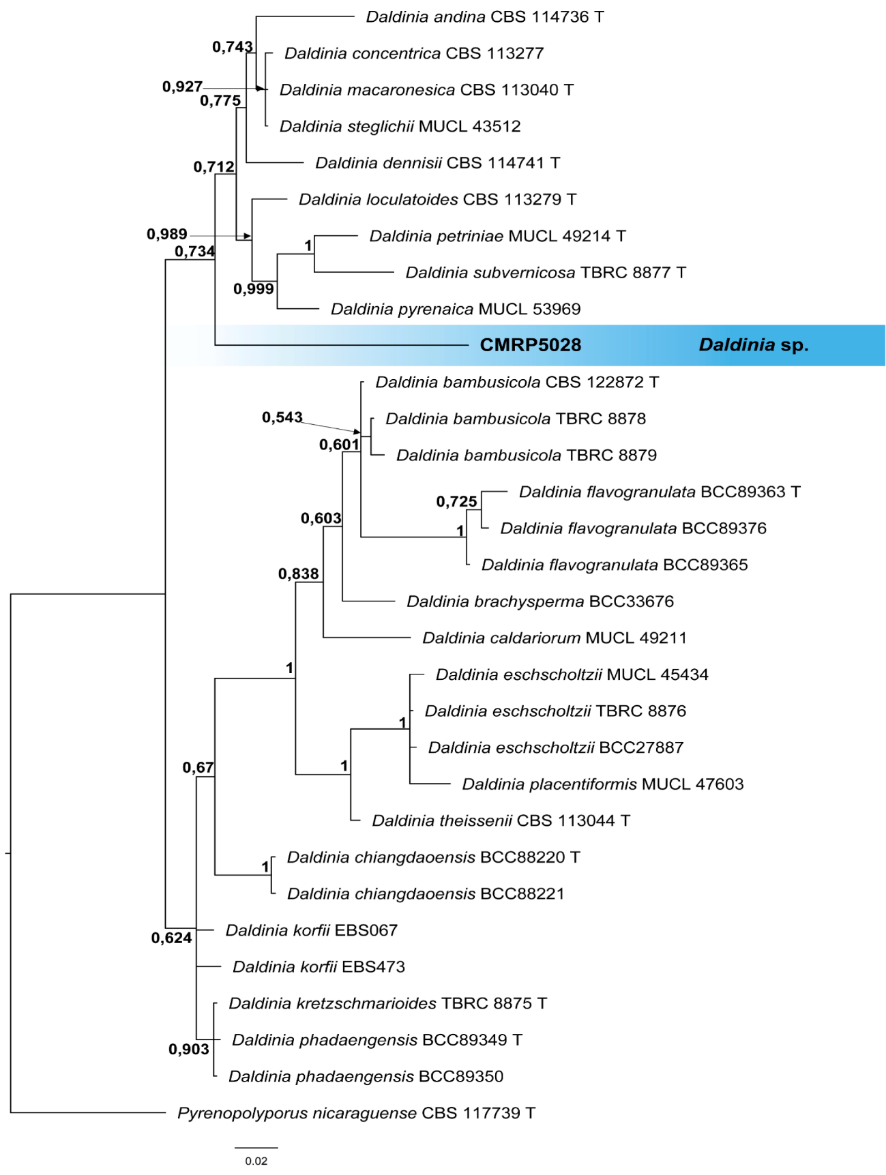


**Figure S34.** Bayesian Inference phylogenetic tree of *Daldinia* species based on the alignment of ITS partial sequence. The data matrix had 31 taxa and 989 characters. The species *Pyrenopolyporus nicaraguense* (CBS 117739) was used as an outgroup. Strains marked with a “T” correspond to type sequences. Bayesian posterior probabilities equal to or greater than 0.50 are presented next to each node in bold. The scale bar of 0.02 represents the number of changes. The sequence of the isolate here studied is presented with its culture collection code (CMRP5028) highlighted in bold.


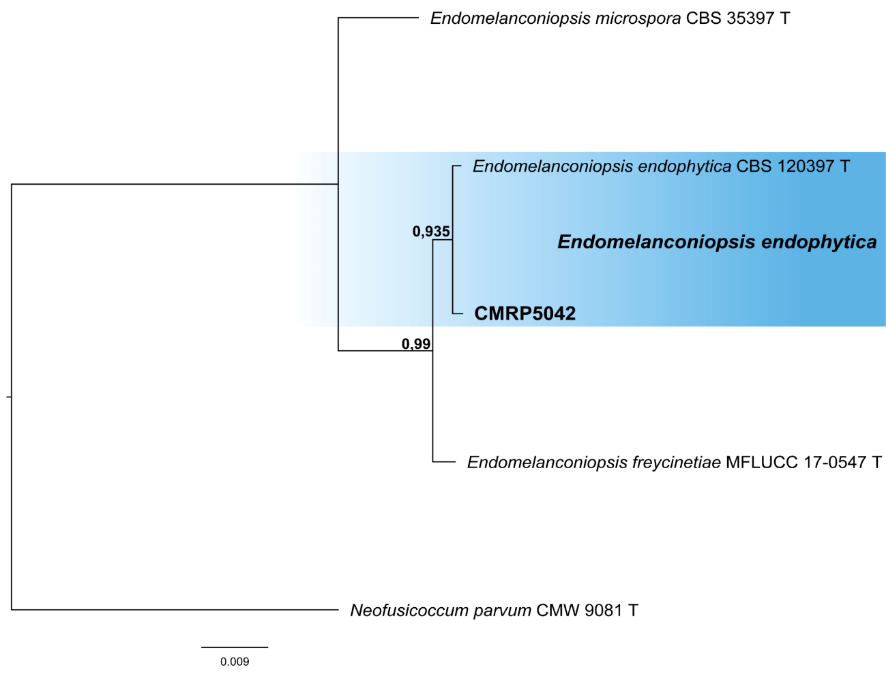


**Figure S35.** Bayesian Inference phylogenetic tree of *Endomelanconiopsis* species based on the alignment of ITS partial sequence. The data matrix had 5 taxa and 658 characters. The species *Neofusicoccum parvum* (CMW 9081) was used as an outgroup. Strains marked with a “T” correspond to type sequences. Bayesian posterior probabilities equal to or greater than 0.50 are presented next to each node in bold. The scale bar of 0.009 represents the number of changes. The sequence of the isolate here studied is presented with its culture collection code (CMRP5042) highlighted in bold.


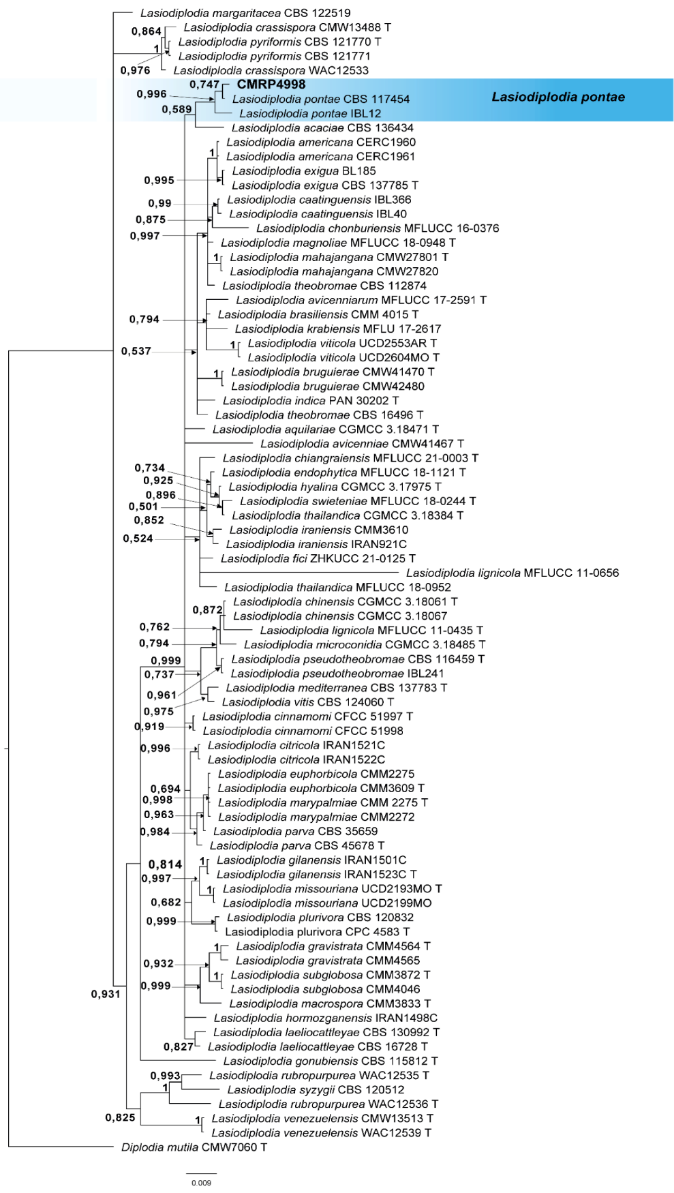


**Figure S36.** Bayesian Inference phylogenetic tree of *Lasiodiplodia* species based on multiple alignment of ITS, *tef1* and *tub2* partial sequence. The data matrix had 76 taxa and 2,085 characters. The species *Diplodia mutila* (CMW 7060) was used as an outgroup. Strains marked with a “T” correspond to type sequences. Bayesian posterior probabilities equal to or greater than 0.50 are presented next to each node in bold. The scale bar of 0.03 represents the number of changes. The sequence of the isolate here studied is presented with its culture collection code (CMRP4998) highlighted in bold.


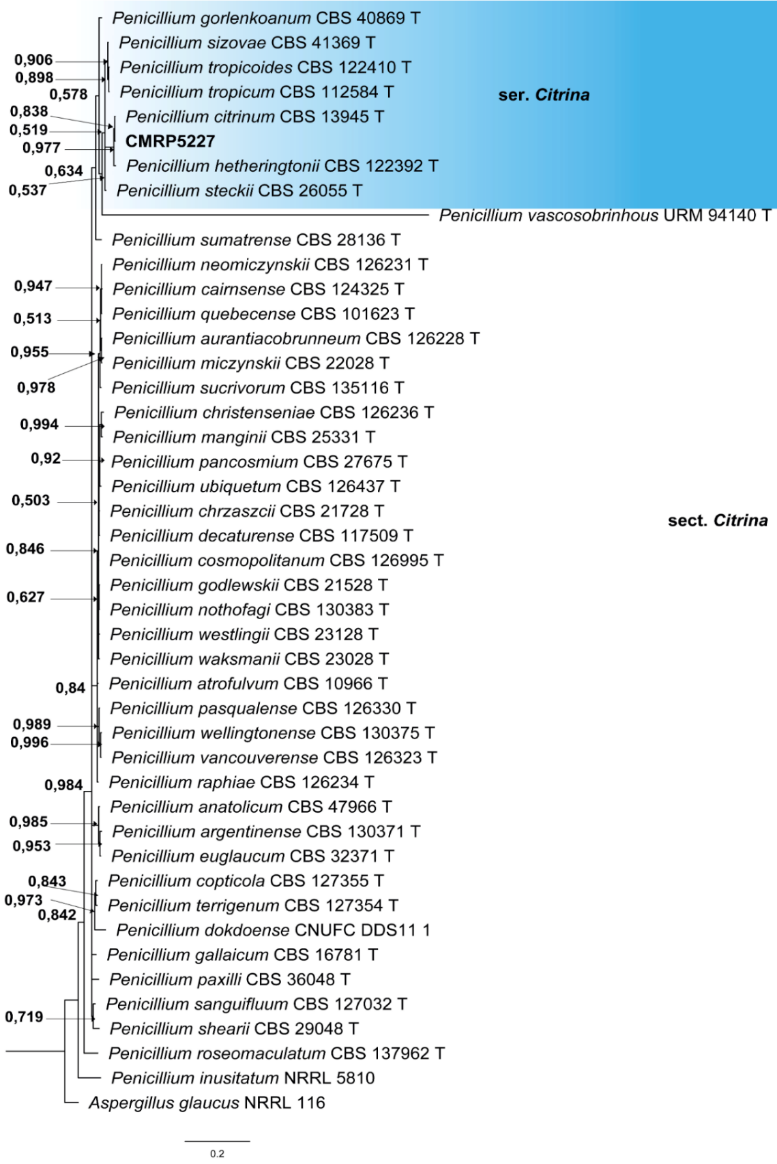


**Figure S37.** Bayesian Inference phylogenetic tree of *Penicillium* section *Citrina* species based on the alignment of ITS partial sequence. The data matrix had 45 taxa and 889 characters. The species *Aspergillus glaucus* (NRRL 116) was used as an outgroup. Strains marked with a “T” correspond to type sequences. Bayesian posterior probabilities equal to or greater than 0.50 are presented next to each node in bold. The scale bar of 0.2 represents the number of changes. The sequence of the isolate here studied is presented with its culture collection code (CMRP5227) highlighted in bold.


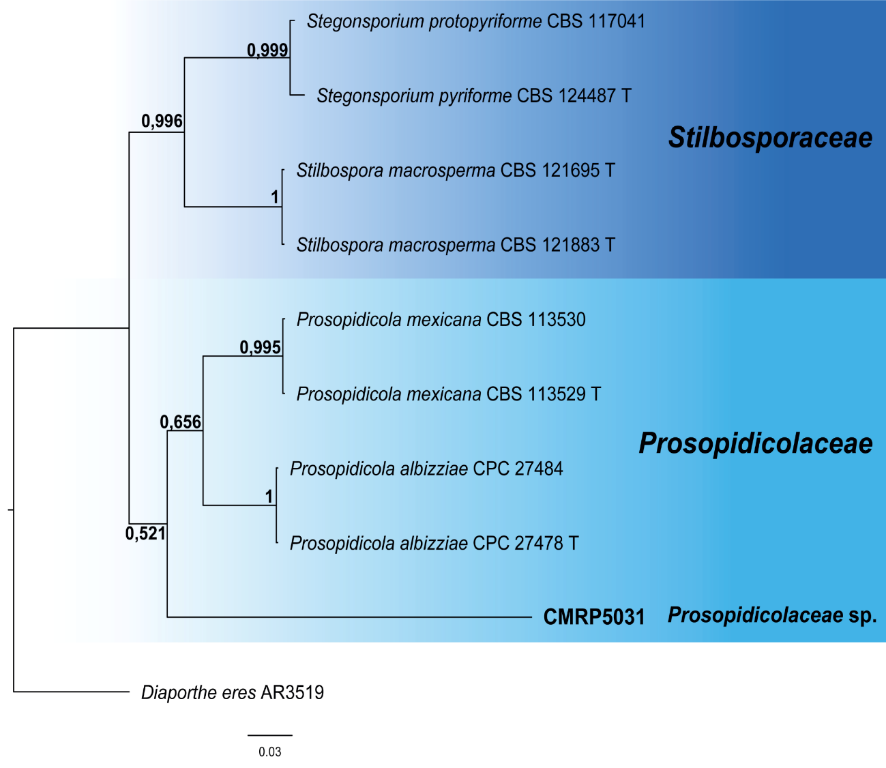


**Figure S38.** Bayesian Inference phylogenetic tree of species from Prosopidicolaceae and Stilbosporaceae families based on the alignment of ITS partial sequence. The data matrix had 10 taxa and 681 characters. The species *Diaporthe eres* (AR3519) was used as an outgroup. Strains marked with a “T” correspond to type sequences. Bayesian posterior probabilities equal to or greater than 0.50 are presented next to each node in bold. The scale bar of 0.03 represents the number of changes. The sequence of the isolate here studied is presented with its culture collection code (CMRP5031) highlighted in bold.


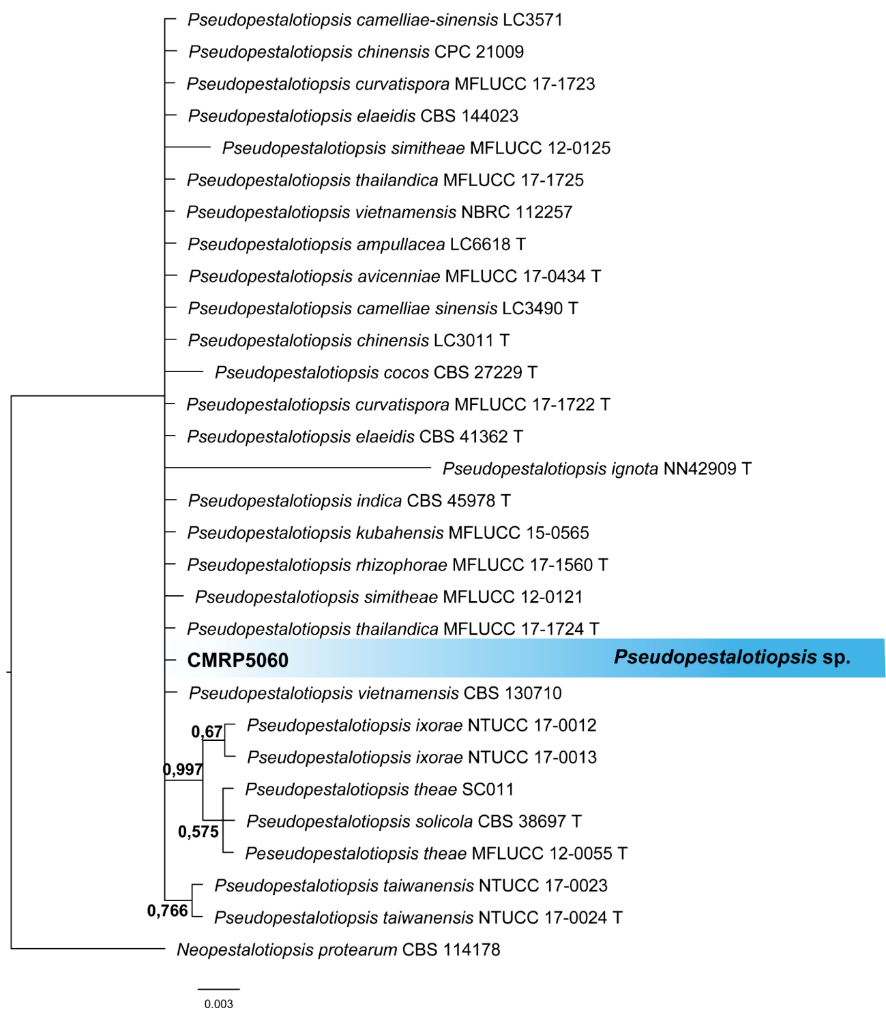


**Figure S39.** Bayesian Inference phylogenetic tree of *Pseudopestalotiopsis* species based on the alignment of ITS partial sequence. The data matrix had 30 taxa and 615 characters. The species *Neopestalotiopsis protearum* (CBS 114178) was used as an outgroup. Strains marked with a “T” correspond to type sequences. Bayesian posterior probabilities equal to or greater than 0.50 are presented next to each node in bold. The scale bar of 0.003 represents the number of changes. The sequence of the isolate here studied is presented with its culture collection code (CMRP5060) highlighted in bold.


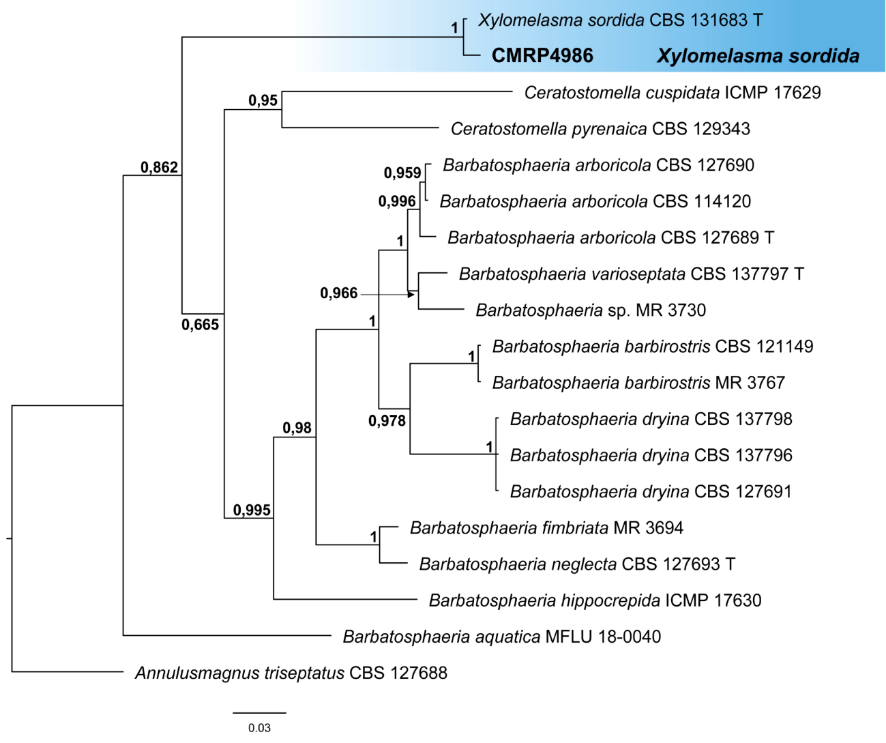


**Figure S40.** Bayesian Inference phylogenetic tree of species from Barbatosphaeriaceae family based on the alignment of ITS partial sequence. The data matrix had 19 taxa and 611 characters. The species *Annulusmagnus triseptatus* (CBS 127688) was used as an outgroup. Strains marked with a “T” correspond to type sequences. Bayesian posterior probabilities equal to or greater than 0.50 are presented next to each node in bold. The scale bar of 0.03 represents the number of changes. The sequence of the isolate here studied is presented with its culture collection code (CMRP4986) highlighted in bold.

**Table S7.** GenBank megablast result for the first 10 sequences using ITS (internal transcribed spacer) partial sequence from CMRP4997 isolate.

| **Strain** | **Culture collection** | **GenBank** | **Query cover (%)** | **Identities (%)** |
| --- | --- | --- | --- | --- |
| *Phomopsis* sp. | CML 1316 | JN153056 | 98 | 99 |
| *Phomopsis* sp. | CML 871 | JN153054 | 98 | 99 |
| *Diaporthe* sp. | Bpf-10 | MH397486 | 100 | 99 |
| Fungal endophyte sp. | g29 | HM537034 | 100 | 99 |
| *Phomopsis* sp. | CML 1930 | JN153072 | 97 | 99 |
| *Phomopsis* sp. | VegaE3-81 | EU002934 | 97 | 99 |
| *Diaporthe velutina* | LC4422 | KX986786 | 98 | 99 |
| *Phomopsis* sp. | F122 | KM979805 | 98 | 99 |
| *Diaporthe foeniculina* | A1901B | MT230445 | 100 | 98 |
| *Diaporthe foeniculina* | A1907B | MT230444 | 100 | 98 |

**Table S8.** GenBank megablast result for the first 10 sequences using *tef1* (translation elongation factor 1-α) partial sequence from CMRP4997 isolate.

| **Strain** | **Culture collection** | **GenBank** | **Query cover (%)** | **Identities (%)** |
| --- | --- | --- | --- | --- |
| *Diaporthe* sp. | CMRP4602 | MZ889475 | 85 | 100 |
| *Diaporthe portugallica* | CPC 34247 | MH063911 | 99 | 94 |
| *Diaporthe* sp. | Fi2341 | KX986873 | 83 | 99 |
| *Diaporthe phillipsii* | CAA817 | MK828076 | 97 | 94 |
| *Phomopsis* sp. | JMS-2010c | GQ250357 | 97 | 94 |
| *Diaporthe anacardii* | CBS 144610 | MK442692 | 98 | 94 |
| *Diaporthe nebulae* | PMM818 | MT458111 | 100 | 93 |
| *Diaporthe macadamiae* | BRIP 66526 | MN696528 | 100 | 93 |
| *Diaporthe macadamiae* | BRIP 66525 | MN696527 | 100 | 93 |
| *Diaporthe nebulae* | Phom240 | MH708543 | 98 | 93 |

**Table S9.** GenBank megablast result for the first 10 sequences using *tub* (tubulin) partial sequence from CMRP4997 isolate.

| **Strain** | **Culture collection** | **GenBank** | **Query cover (%)** | **Identities (%)** |
| --- | --- | --- | --- | --- |
| *Diaporthe* sp. | UFMGCB 7241 | MT407179 | 99 | 99 |
| *Diaporthe* sp. | UFMGCB 9826 | KU992321 | 97 | 99 |
| *Diaporthe* sp. | UFMGCB 7401 | MT407178 | 93 | 99 |
| *Diaporthe velutina* | PSCG 134 | MK691243 | 99 | 97 |
| *Diaporthe velutina* | PSCG 417 | MK691244 | 99 | 97 |
| *Diaporthe velutina* | LC4788 | KX999218 | 98 | 97 |
| *Diaporthe macadamiae* | BRIP 66526 | MN696539 | 98 | 97 |
| *Diaporthe foeniculina* | ColPat-560 | MK522116 | 99 | 96 |
| *Diaporthe foeniculina* | ColPat-559 | MK522115 | 99 | 96 |
| *Diaporthe foeniculina* | ColPat-555 | MK522114 | 99 | 96 |

**Table S10.** GenBank megablast result for the first 10 sequences using *his3* (histone H3) partial sequence from CMRP4997 isolate.

| **Strain** | **Culture collection** | **GenBank** | **Query cover (%)** | **Identities (%)** |
| --- | --- | --- | --- | --- |
| *Diaporthe velutina* | PSCG 134 | MK726205 | 97 | 96 |
| *Diaporthe velutina* | LC4422 | KX999257 | 96 | 96 |
| *Diaporthe anacardii* | CBS 72097 | KC343508 | 98 | 95 |
| *Diaporthe velutina* | PSCG 417 | MK726206 | 97 | 95 |
| *Diaporthe velutina* | LC4414 | KX999259 | 96 | 95 |
| *Diaporthe velutina* | LC4788 | KX999256 | 96 | 95 |
| *Diaporthe stictica* | CBS 37054 | KC343696 | 97 | 95 |
| *Diaporthe phillipsii* | CAA817 | MK871445 | 97 | 95 |
| *Diaporthe velutina* | LC6519 | KX999262 | 96 | 95 |
| *Diaporthe velutina* | LC4419 | KX999260 | 96 | 95 |


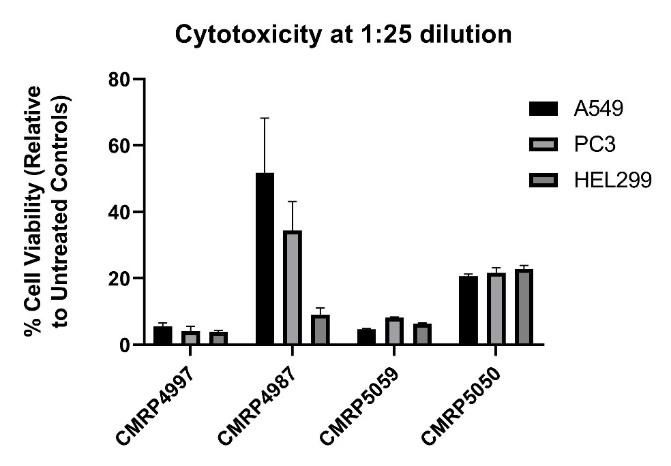


**Figure S41.** % Viability of A549 (non-small lung), PC3 (prostate) and HEL299 (human lung fibroblast) cell lines (after 72h) at 1:25 dilution of the fungal extracts produced by *Diaporthe* *amolarensis* sp. nov. CMRP4997, *Nemania primolutea* CMRP4987, *Xylaria arbuscula* CMRP5059, *Xylaria arbuscula* CMRP5050.


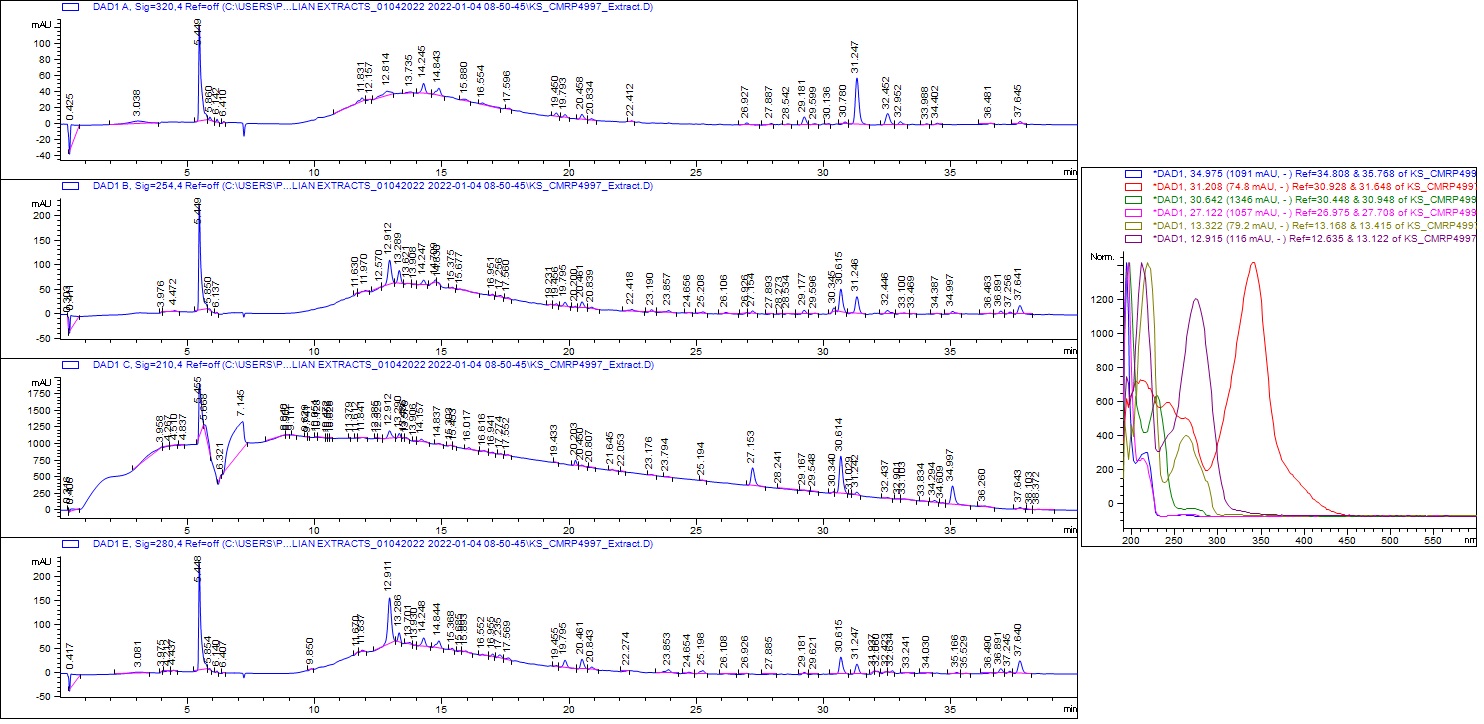


**Figure S42.** HPLC/UV analyses of the extract produced by *Diaporthe* *amolarense* sp. nov. CMRP4997. HPLC-conditions: solvent A: H_2_O/0.1% FA; solvent B: CH_3_CN; flow rate: 0.5 mL min^-1^; 0-30 min, 5-100% B (linear gradient); 30-35 min, 100% B; 35-36 min, 100-5% B (linear gradient); 36-40 min, 5% B; Detection wavelength: 320, 254, 210, 280 nm. UV-vis inset of full wavelength scan (190-600 nm).


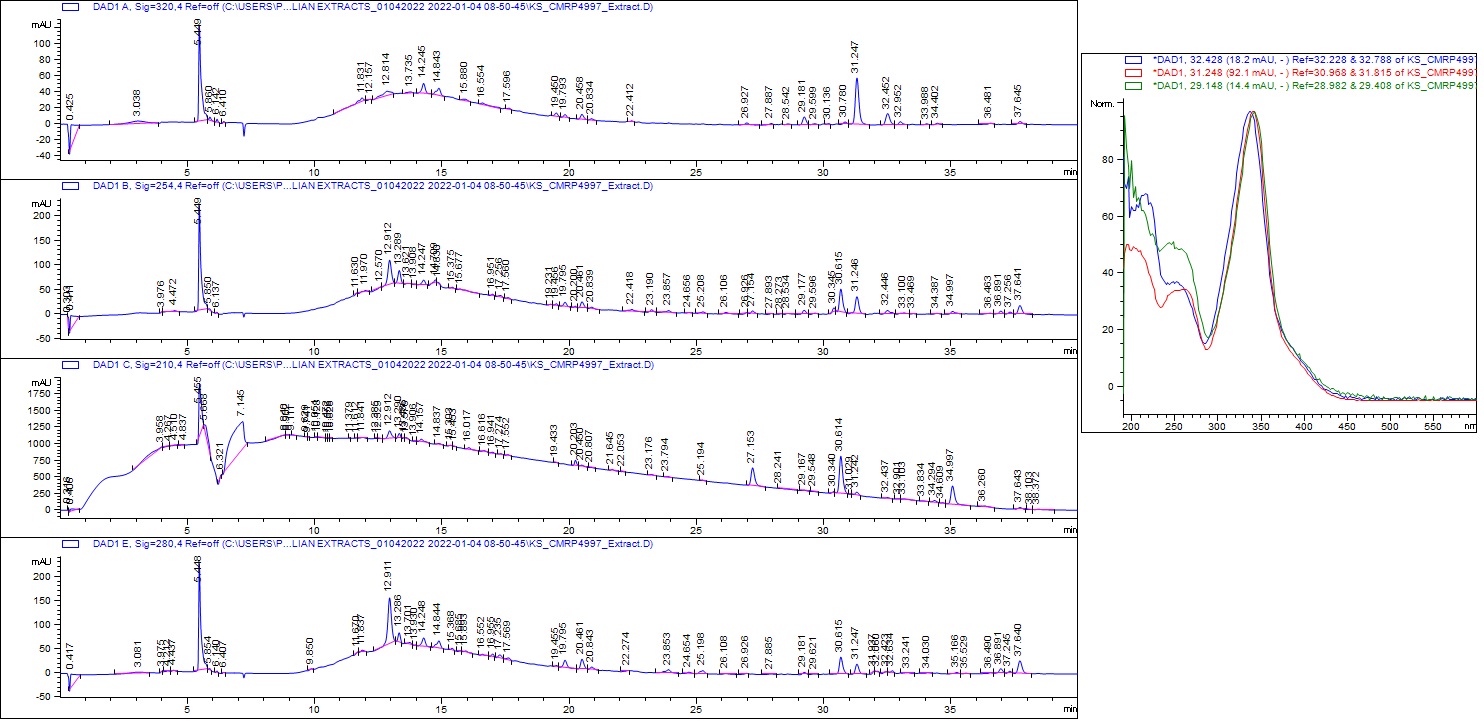


**Figure S43.** HPLC/UV analyses of the extract produced by *Diaporthe* *amolarense* sp. nov. CMRP4997. HPLC-conditions: solvent A: H_2_O/0.1% FA; solvent B: CH_3_CN; flow rate: 0.5 mL min^-1^; 0-30 min, 5-100% B (linear gradient); 30-35 min, 100% B; 35-36 min, 100-5% B (linear gradient); 36-40 min, 5% B; Detection wavelength: 320, 254, 210, 280 nm. UV-vis inset of full wavelength scan (190-600 nm).

**RT = 13 min**

**RT = 14.2 min**

**[2M+H]^+^**

**[M-H]**^−^

**[M+H]^+^**

**MW: 196**

**Figure S44.** (+) and (–)-ESI-MS spectra of the compounds detected in the extract produced by *Diaporthe* *amolarense* sp. nov. CMRP4997. LCMS-conditions: solvent A: H_2_O/0.1% FA; solvent B: CH_3_CN; flow rate: 0.5 mL min^-1^; 0-30 min, 5-100% B (linear gradient); 30-35 min, 100% B; 35-36 min, 100-5% B (linear gradient); 36-40 min, 5% B. Note – no clear mass peaks were detected in the (–)-ESI-MS. RT = **R**etention **T**ime

**RT = 14.9 min**

**RT = 20.0 min**

**MW: 253**

**[M+HCOO]**^−^

**[M+H]^+^**

**Figure S45.** (+) and (–)-ESI-MS spectra of the compounds detected in the extract produced by *Diaporthe* *amolarense* sp. nov. CMRP4997. LCMS-conditions: solvent A: H_2_O/0.1% FA; solvent B: CH_3_CN; flow rate: 0.5 mL min^-1^; 0-30 min, 5-100% B (linear gradient); 30-35 min, 100% B; 35-36 min, 100-5% B (linear gradient); 36-40 min, 5% B. RT = **R**etention **T**ime

**MW: 296**

**RT = 31.1 min**

**[(M-H_2_O)+H]^+^**

**[M-H]**^−^

**[M+Cl]**^−^

**[M+HCOO]**^−^

**[M+H]^+^**

**RT = 27 min**

**MW: 414**

**Figure S46.** (+) and (–)-ESI-MS spectra of the compounds detected in the extract produced by *Diaporthe* *amolarense* sp. nov. CMRP4997. LCMS-conditions: solvent A: H_2_O/0.1% FA; solvent B: CH_3_CN; flow rate: 0.5 mL min^-1^; 0-30 min, 5-100% B (linear gradient); 30-35 min, 100% B; 35-36 min, 100-5% B (linear gradient); 36-40 min, 5% B. RT = **R**etention **T**ime


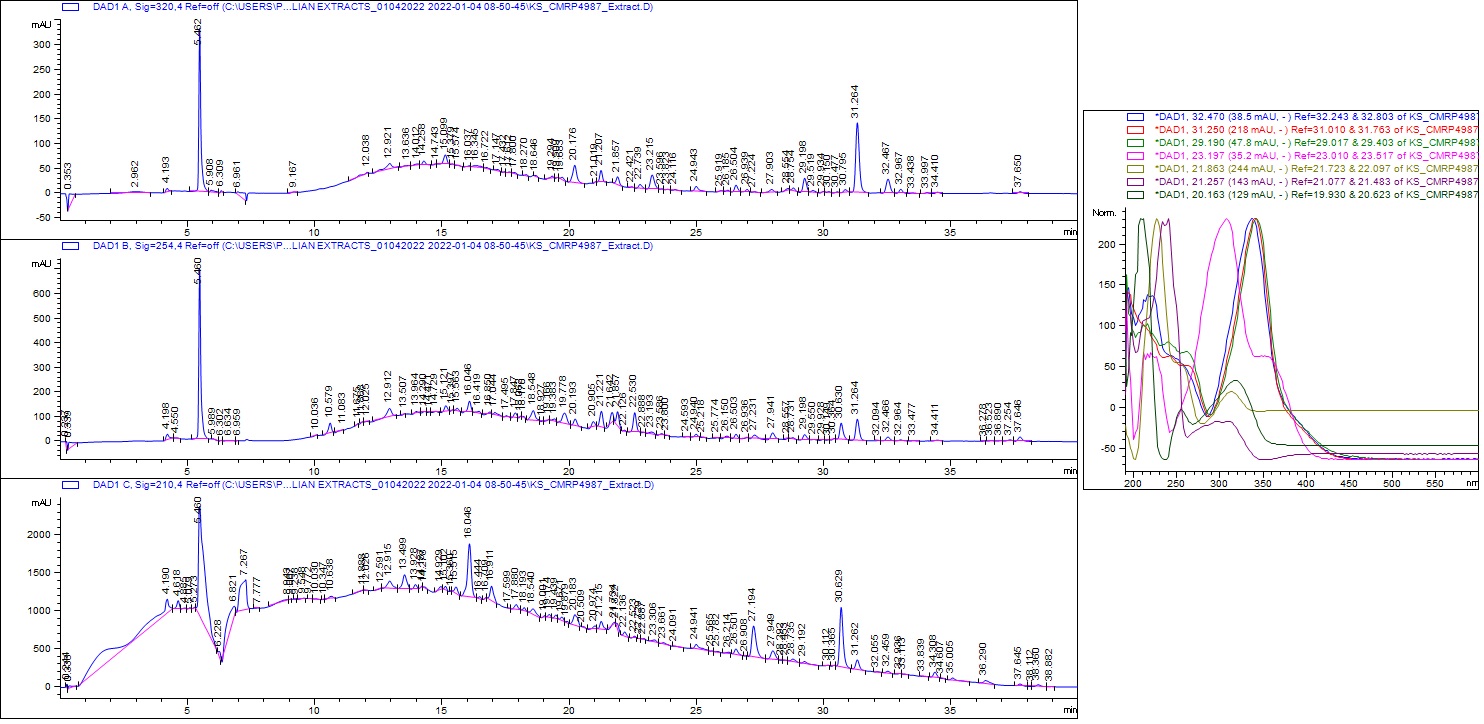


**Figure S47.** HPLC/UV analyses of the extract produced by *Nemania primolutea* CMRP4987. HPLC-conditions: solvent A: H_2_O/0.1% FA; solvent B: CH_3_CN; flow rate: 0.5 mL min^-1^; 0-30 min, 5-100% B (linear gradient); 30-35 min, 100% B; 35-36 min, 100-5% B (linear gradient); 36-40 min, 5% B; Detection wavelength: 320, 254, 210 nm. UV-vis inset of full wavelength scan (190-600 nm).

**[M-H]**^−^

**[M+H]^+^**

**RT = 17.9 min**

**MW: 431**

**RT = 16 .1min**

**Figure S48.** (+) and (–)-ESI-MS spectra of the compounds detected in the extract produced by *Nemania primolutea* CMRP4987. LCMS-conditions: solvent A: H_2_O/0.1% FA; solvent B: CH_3_CN; flow rate: 0.5 mL min^-1^; 0-30 min, 5-100% B (linear gradient); 30-35 min, 100% B; 35-36 min, 100-5% B (linear gradient); 36-40 min, 5% B. Note – no clear mass was detected for peak at RT = 17.9 min. RT = **R**etention **T**ime

**[M-H]**^−^

**[M+H]^+^**

**RT = 27.3 min**

**MW: 332**

**RT = 22.4 min**

**[M+H]^+^**

**MW: 325**

**Figure S49.** (+) and (–)-ESI-MS spectra of the compounds detected in the extract produced by *Nemania primolutea* CMRP4987. LCMS-conditions: solvent A: H_2_O/0.1% FA; solvent B: CH_3_CN; flow rate: 0.5 mL min^-1^; 0-30 min, 5-100% B (linear gradient); 30-35 min, 100% B; 35-36 min, 100-5% B (linear gradient); 36-40 min, 5% B. RT = **R**etention **T**ime

**RT = 28 min**

**MW: 290**

**[M+H]^+^**

**[(M-2H_2_O)+H]^+^**

**[(M-H_2_O)+H]^+^**

**RT = 30.6 min**

**Figure S50.** (+) and (–)-ESI-MS spectra of the compounds detected in the extract produced by *Nemania primolutea* CMRP4987. LCMS-conditions: solvent A: H_2_O/0.1% FA; solvent B: CH_3_CN; flow rate: 0.5 mL min^-1^; 0-30 min, 5-100% B (linear gradient); 30-35 min, 100% B; 35-36 min, 100-5% B (linear gradient); 36-40 min, 5% B. Note – no clear mass was detected for peak at RT = 28 min. RT=**R**etention **T**ime

**RT = 29.2 min**

**RT = 21.8 min**

**[(M-sugar)+H]^+^**

**MW: 478**

**[M-H]**^−^

**[(M-H_2_O)+H]^+^**

**[M-H]**^−^

**MW: 666**

**[M+H]^+^**

**Figure S51.** (+) and (–)-ESI-MS spectra of the compounds detected in the extract produced by *Nemania primolutea* CMRP4987. LCMS-conditions: solvent A: H_2_O/0.1% FA; solvent B: CH_3_CN; flow rate: 0.5 mL min^-1^; 0-30 min, 5-100% B (linear gradient); 30-35 min, 100% B; 35-36 min, 100-5% B (linear gradient); 36-40 min, 5% B. Note – no clear mass was detected for peak at RT = 28 min. RT=**R**etention **T**ime

**Figure S52.** (+) and (–)-ESI-MS spectra of the compounds detected in the extract produced by *Nemania primolutea* CMRP4987. LCMS-conditions: solvent A: H_2_O/0.1% FA; solvent B: CH_3_CN; flow rate: 0.5 mL min^-1^; 0-30 min, 5-100% B (linear gradient); 30-35 min, 100% B; 35-36 min, 100-5% B (linear gradient); 36-40 min, 5% B. Note – no clear mass was detected for peaks at RT = 31.3 and 32.6 min. RT = **R**etention **T**ime


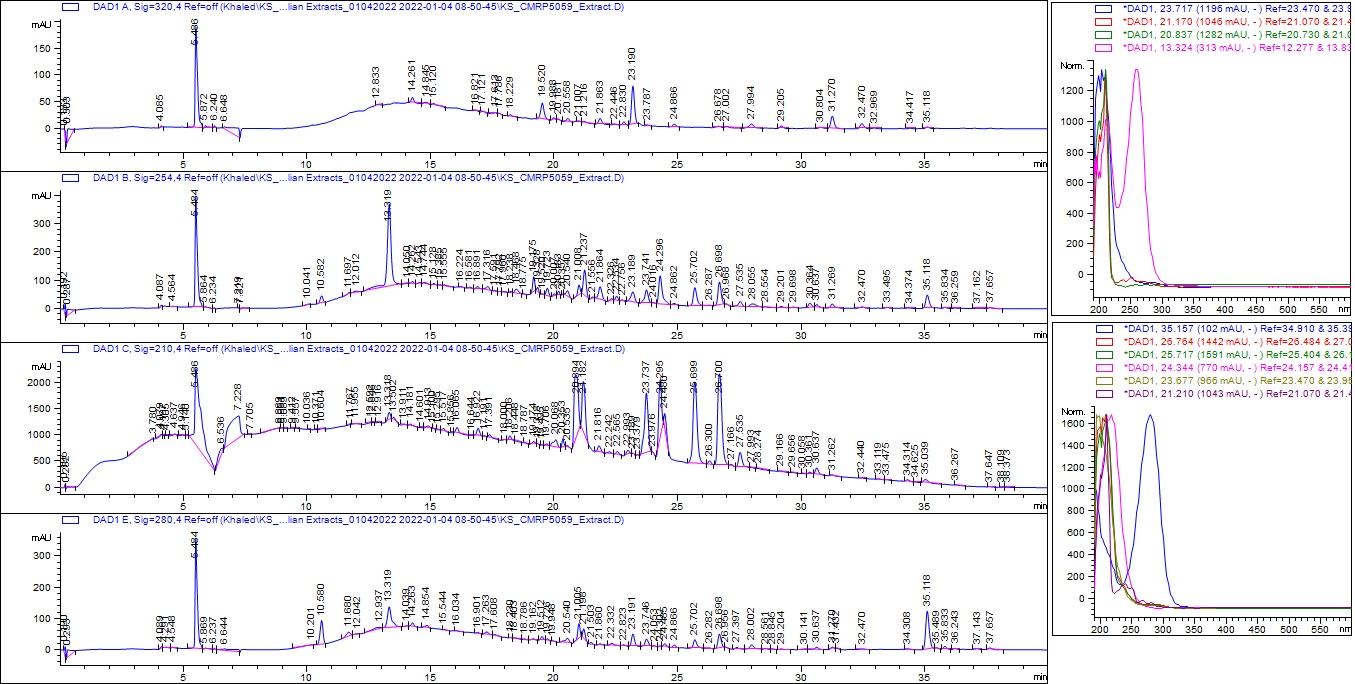


**Figure S53.** HPLC/UV analyses of the extract produced by *Xylaria arbuscula* CMRP5059. HPLC-conditions: solvent A: H_2_O/0.1% FA; solvent B: CH_3_CN; flow rate: 0.5 mL min^-1^; 0-30 min, 5-100% B (linear gradient); 30-35 min, 100% B; 35-36 min, 100-5% B (linear gradient); 36-40 min, 5% B; Detection wavelength: 320, 254, 210, 280 nm. UV-vis inset of full wavelength scan (190-600 nm).

**[M-H]**^−^

**[M+H]^+^**

**MW: 260**

**RT = 13.5 min**

**RT = 5.5 min**

**MW: 253**

**[M+H]^+^**

**Figure S54.** (+) and (–)-ESI-MS spectra of the compounds detected in the extract produced by *Xylaria arbuscula* CMRP5059. LCMS-conditions: solvent A: H_2_O/0.1% FA; solvent B: CH_3_CN; flow rate: 0.5 mL min^-1^; 0-30 min, 5-100% B (linear gradient); 30-35 min, 100% B; 35-36 min, 100-5% B (linear gradient); 36-40 min, 5% B.

**RT = 21.0 min**

**MW: 303?**

**[M+H]^+^**

**RT = 21.2 min**

**[2M+H]^+^**

**MW: 507**

**[M+H]^+^**

**Figure S55.** (+) and (–)-ESI-MS spectra of the compounds detected in the extract produced by *Xylaria arbuscula* CMRP5059. LCMS-conditions: solvent A: H_2_O/0.1% FA; solvent B: CH_3_CN; flow rate: 0.5 mL min^-1^; 0-30 min, 5-100% B (linear gradient); 30-35 min, 100% B; 35-36 min, 100-5% B (linear gradient); 36-40 min, 5% B. Note – no clear mass peaks were detected in the (–)-ESI-MS.

**[M+H]^+^**

**MW: 414 or 542?**

**[M+H]^+^**

**RT = 24.3 min**

**RT = 21.9 min**

**[M+H]^+^**

**MW: 331**

**Figure S56.** (+) and (–)-ESI-MS spectra of the compounds detected in the extract produced by *Xylaria arbuscula* CMRP5059. LCMS-conditions: solvent A: H_2_O/0.1% FA; solvent B: CH_3_CN; flow rate: 0.5 mL min^-1^; 0-30 min, 5-100% B (linear gradient); 30-35 min, 100% B; 35-36 min, 100-5% B (linear gradient); 36-40 min, 5% B. Note – no clear mass peaks were detected in the (–)-ESI-MS.

**MW: 523**

**[2M+H]^+^**

**[2M+H]^+^**

**[M-H]**^−^

**[M+H]^+^**

**RT = 25.7 min**

**RT = 26.7 min**

**[(M-H_2_O)+H]^+^**

**[M-H]**^−^

**[M+H]^+^**

**MW: 507**

**Figure S57.** (+) and (–)-ESI-MS spectra of the compounds detected in the extract produced by *Xylaria arbuscula* CMRP5059. LCMS-conditions: solvent A: H_2_O/0.1% FA; solvent B: CH_3_CN; flow rate: 0.5 mL min^-1^; 0-30 min, 5-100% B (linear gradient); 30-35 min, 100% B; 35-36 min, 100-5% B (linear gradient); 36-40 min, 5% B.

**[(M-H_2_O)+H]^+^**

**[2M+H]^+^**

**RT = 25.7 min**

**MW: 523**

**[M+H]^+^**

**[2M+H]^+^**

**[M-H]**^−^

**RT = 23.7 min**

**MW: 507**

**Figure S58.** (+) and (–)-ESI-MS spectra of the compounds detected in the extract produced by *Xylaria arbuscula* CMRP5059. LCMS-conditions: solvent A: H_2_O/0.1% FA; solvent B: CH_3_CN; flow rate: 0.5 mL min^-1^; 0-30 min, 5-100% B (linear gradient); 30-35 min, 100% B; 35-36 min, 100-5% B (linear gradient); 36-40 min, 5% B.


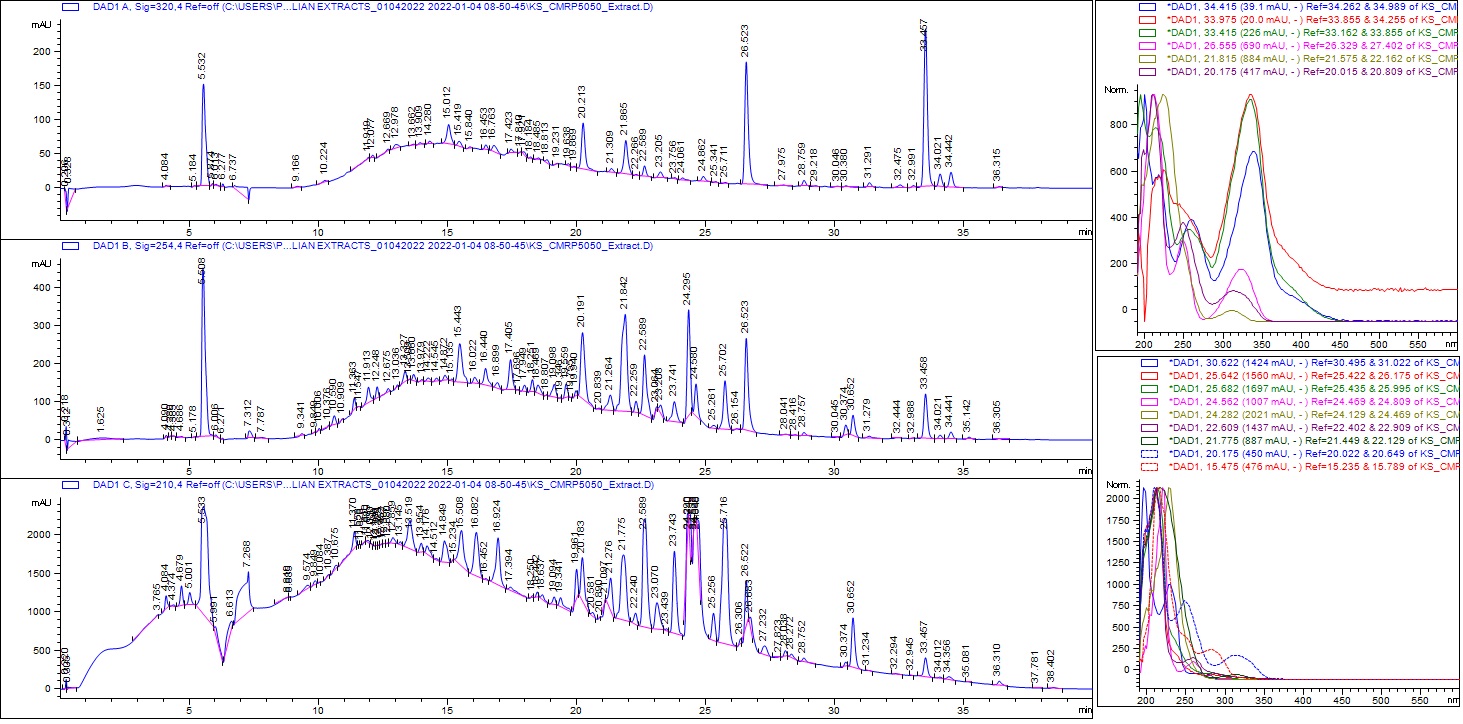


**Figure S59.** HPLC/UV analyses of the extract produced by *Xylaria arbuscula* CMRP5050. HPLC-conditions: solvent A: H_2_O/0.1% FA; solvent B: CH_3_CN; flow rate: 0.5 mL min^-1^; 0-30 min, 5-100% B (linear gradient); 30-35 min, 100% B; 35-36 min, 100-5% B (linear gradient); 36-40 min, 5% B; Detection wavelength: 320, 254, 210 nm. UV-vis inset of full wavelength scan (190-600 nm).


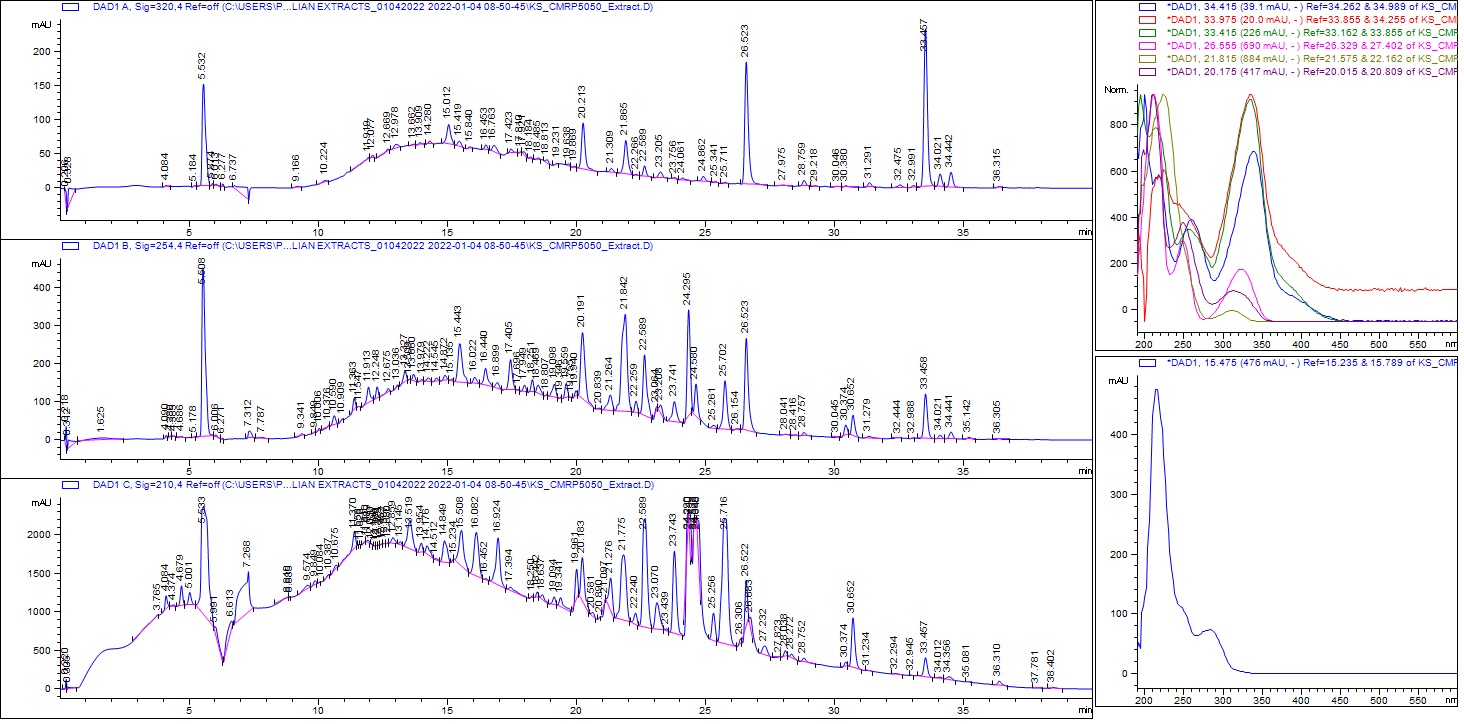


**Figure S60.** HPLC/UV analyses of the extract produced by *Xylaria arbuscula* CMRP5050. HPLC-conditions: solvent A: H_2_O/0.1% FA; solvent B: CH_3_CN; flow rate: 0.5 mL min^-1^; 0-30 min, 5-100% B (linear gradient); 30-35 min, 100% B; 35-36 min, 100-5% B (linear gradient); 36-40 min, 5% B; Detection wavelength: 320, 254, 210 nm. UV-vis inset of full wavelength scan (190-600 nm).

**RT = 16.1 min**

**MW: 552**

**[M+H]^+^**

**[M-H]**^−^

**MW: 416**

**RT = 15.5 min**

**[M+H]^+^**

**[M-H]**^−^

**Figure S61.** (+) and (–)-ESI-MS spectra of the compounds detected in the extract produced by *Xylaria arbuscula* CMRP5050. LCMS-conditions: solvent A: H_2_O/0.1% FA; solvent B: CH_3_CN; flow rate: 0.5 mL min^-1^; 0-30 min, 5-100% B (linear gradient); 30-35 min, 100% B; 35-36 min, 100-5% B (linear gradient); 36-40 min, 5% B.

**[M+H]^+^**

**MW: 539**

**RT = 21.8 min**

**[M-H]**^−^

**MW: 541**

**[M+H]^+^**

**RT = 20.2 min**

**Figure S62.** (+) and (–)-ESI-MS spectra of the compounds detected in the extract produced by *Xylaria arbuscula* CMRP5050. LCMS-conditions: solvent A: H_2_O/0.1% FA; solvent B: CH_3_CN; flow rate: 0.5 mL min^-1^; 0-30 min, 5-100% B (linear gradient); 30-35 min, 100% B; 35-36 min, 100-5% B (linear gradient); 36-40 min, 5% B.

**[M-H]**^−^

**[(M-H_2_O)+H]^+^**

**[2M-H]**^−^

**[M-H]**^−^

**RT = 24.3 min**

**MW: 432**

**RT = 22.6 min**

**[M+H]^+^**

**MW: 523**

**Figure S63.** (+) and (–)-ESI-MS spectra of the compounds detected in the extract produced by *Xylaria arbuscula* CMRP5050. LCMS-conditions: solvent A: H_2_O/0.1% FA; solvent B: CH_3_CN; flow rate: 0.5 mL min^-1^; 0-30 min, 5-100% B (linear gradient); 30-35 min, 100% B; 35-36 min, 100-5% B (linear gradient); 36-40 min, 5% B.

**[2M+H]^+^**

**[(M-H_2_O)+H]^+^**

**MW: 523**

**RT = 25.7 min**

**[M-H]**^−^

**RT = 24.7 min**

**MW: 523**

**[M+H]^+^**

**Figure S64.** (+) and (–)-ESI-MS spectra of the compounds detected in the extract produced by *Xylaria arbuscula* CMRP5050. LCMS-conditions: solvent A: H_2_O/0.1% FA; solvent B: CH_3_CN; flow rate: 0.5 mL min^-1^; 0-30 min, 5-100% B (linear gradient); 30-35 min, 100% B; 35-36 min, 100-5% B (linear gradient); 36-40 min, 5% B.

**MW: 314**

**RT = 30.4 min**

**[M-H]**^−^

**[M+H]^+^**

**[M+H]^+^**

**RT = 26.6 min**

**MW: 523**

**[M-H]**^−^

**Figure S65.** (+) and (–)-ESI-MS spectra of the compounds detected in the extract produced by *Xylaria arbuscula* CMRP5050. LCMS-conditions: solvent A: H_2_O/0.1% FA; solvent B: CH_3_CN; flow rate: 0.5 mL min^-1^; 0-30 min, 5-100% B (linear gradient); 30-35 min, 100% B; 35-36 min, 100-5% B (linear gradient); 36-40 min, 5% B.

**Figure S66.** (+) and (–)-ESI-MS spectra of the compounds detected in the extract produced by *Xylaria arbuscula* CMRP5050. LCMS-conditions: solvent A: H_2_O/0.1% FA; solvent B: CH_3_CN; flow rate: 0.5 mL min^-1^; 0-30 min, 5-100% B (linear gradient); 30-35 min, 100% B; 35-36 min, 100-5% B (linear gradient); 36-40 min, 5% B. Note – no clear mass peaks were detected for RT=30.7 and 33.6 min.

**REFERENCES**

Crous, P.W., Groenewald, J.Z., Shivas, R.G., Edwards, J., Seifert, K.A., Alfenas, A.C., Alfenas, R.F., et al. (2011). Fungal Planet description sheets: 69-91. Persoonia. 26, 108-156. doi: 10.3767/003158511X581723

Crous, P.W., Wingfield, M.J., Guarro, J., Cheewangkoon, R., van der Bank, M., Swart, W.J., Stchigel, A.M. et al., (2013). Fungal Planet description sheets: 154-213. Persoonia. 31, 188-296. doi: 10.3767/003158513X675925

Crous, P.W., Shivas, R.G., Quaedvlieg, W., van der Bank, M., Zhang, Y., Summerell, B.A., Guarro, J., et al. (2014). Fungal Planet description sheets: 214-280. Persoonia. 32, 184-306. doi: 10.3767/003158514X682395

Crous, P.W., Schumacher, R.K., Akulov, A., Thangavel, R., Hernandez-Restrepo, M., Carnegie, A.J., Cheewangkoon, R., et al. (2019a). New and Interesting Fungi. 2. Fungal Syst Evol ., 57-134. doi: 10.3114/fuse.2019.03.06

Dayarathne, M.C., Jones, E.B.G., Maharachchikumbura, S.S.N., Devadatha, B., Sarma, V.V., Khongphinitbunjong, K., Chomnunti, P., et al. (2020). Morpho-molecular characterization of microfungi associated with marine based habitats. Mycosphere. 11:1, 1-188. doi: 10.5943/mycosphere/11/1/1

Disanayake, A.J., Camporesi, E., Hyde, K.D., Zhang, W., Yan, J.Y. and Li, X.H. (2017). Molecular phylogenetic analysis reveals seven new *Diaporthe* species from Italy. Mycosphere. 8:5, 853-877. doi: 10.5943/mycosphere/8/5/4

Gao, H., Pan, M., Tian, C., Fan, X. (2021). *Cytospora* and *Diaporthe* species associated with hazelnut canker and dieback in Beijing, China. Front Cell Infect Microbiol. 2:11:664366. doi: 10.3389/fcimb.2021.664366

Guarnaccia, V. and Crous, P.W. (2018). Species of *Diaporthe* on Camellia and Citrus in the Azores Islands. Phytopathol. Mediterr. 57:2, 307-319. doi: 10.14601/Phytopathol_Mediterr-23254

Guo, Y.S., Crous, P.W., Bai, Q., Fu, M., Yang, M.M., Wang, X.H., Du, Y.M., et al. (2020). High diversity of *Diaporthe* species associated with pear shoot canker in China. Persoonia. 45, 132-162. doi: 10.3767/persoonia.2020.45.05

Hilário, S., Amaral, I.A., Goncalves, M.F.M., Lopes, A., Santos, L. and Alves, A. (2020). *Diaporthe* species associated with twig blight and dieback of *Vaccinium corymbosum* in Portugal, with description of four new species. Mycologia. 1-16. doi: 10.1080/00275514.2019.1668906

Hsieh, H.M., Lin, C.R., Fang, M.J., Rogers, J.D., Fournier, J., Lechat, C. and Ju, Y.M. (2010). Phylogenetic status of *Xylaria* subgenus *Pseudoxylaria* among taxa of the subfamily Xylarioideae (Xylariaceae) and phylogeny of the taxa involved in the subfamily. Mol. Phylogenet. Evol. 54:3, 957-969. doi: 10.1016/j.ympev.2009.12.015

Jaklitsch, W.M. and Voglmayr, H. (2012). Phylogenetic relationships of five genera of Xylariales and *Rosasphaeria* gen. nov. (Hypocreales). Fungal Divers. 52, 75-98. doi: 10.1007/s13225-011-0104-2

Jaklitsch, W.M., Gardiennet, A. and Voglmayr, H. (2016). Resolution of morphology-based taxonomic delusions: *Acrocordiella*, *Basiseptospora*, *Blogiascospora*, *Clypeosphaeria*, *Hymenopleella*, *Lepteutypa*, *Pseudapiospora*, *Requienella*, *Seiridium* and *Strickeria*. Persoonia. 37, 82-105. doi: 10.3767/003158516X690475

Ju, Y.M., Hsieh, H.M., Rogers, J.D., Fournier, J., Jaklitsch, W.M. and Courtecuisse, R. (2012). New and interesting penzigioid *Xylaria* species with small, soft stromata. Mycologia, 104:3, 766-76. doi: 10.3852/11-313.

Lesuthu, P., Mostert, L., Spies, C.F.J., Moyo, P. and Halleen, F. (2017). *Diaporthe* sp. nov. and first report of *D. cynaroidis*, *D. novem* and *D. serafiniae* on grapevines in South Africa. Plant Dis. 103:5, 808-817. doi: 10.1094/PDIS-03-18-0433-RE

Lombard, L., van Leeuwen, G.C.M., Guarnaccia, V., Polizzi, G., van Rijswick, P.C.J., Rosendahl, K.C.H.M., Gabler, J. et al. (2014). *Diaporthe* species associated with *Vaccinium* spp. in Europe. Phytopathol. Mediterr*.* 53:2, 287–299. doi: 10.14601/Phytopathol_Mediterr-14034

Peláez, F., Gonzalez, V., Platas, G. and Sanchez-Ballesteros, J. (2008). Molecular phylogenetic studies within the family Xylariaceae based on ribosomal DNA sequences. Fungal Divers. 31, 111-134.

Sánchez-Ballesteros, J., Gonzalez, V., Salazar, O., Acero, J., Portal, M.A., Julian, M., Rubio, V., et al., (2000). Phylogenetic study of *Hypoxylon* and related genera based on ribosomal ITS sequences. Mycologia 92:5, 964-977. doi: 10.1080/00275514.2000.12061240

Senanayake, I.C., Maharachchikumbura, S.S.N., Hyde, K.D., Bhat, J.D., Gareth Jones, E.B., McKenzie, E.H.C., Dai, D.Q., et al. (2015). Towards unraveling relationships in Xylariomycetidae (Sordariomycetes). Fungal Divers. 73, 73-144. doi: 10.1007/s13225-015-0340-y

Stadler, M., Kuhnert, E., Persoh, D. and Fournier, J. (2013). The Xylariaceae as model example for a unified nomenclature following the One Fungus- One Name (1F1N) Concept. Mycology. 4:1. doi: 10.1080/21501203.2013.782478

Tang, A.M., Jeewon, R. and Hyde, K.D. (2007). Phylogenetic relationships of Nemania plumbea sp. nov. and related taxa based on ribosomal ITS and RPB2 sequences. Mycol. Res. 111:4, 392-402. doi: 10.1016/j.mycres.2007.01.009

Tang, A.M.C., Jeewon, R. and Hyde, K.D. (2009). A reevaluation of the evolutionary relationships within the Xylariaceae based on ribosomal and protein-coding gene sequences. Fungal Diversity. 34:127-155.

Thambugala, K.M., Daranagama, D.A., Phillips, A.J.L., Bulgakov, T.S., Bhat, D.J., Camporesi, E., Bahkali, A.H., et al. (2016). Microfungi on Tamarix. Fungal Diversity. 82, 239–306. doi: 10.1007/s13225-016-0371-z

Thompson, S.M., Tan, Y.P., Shivas, R.G., Neate, S.M., Morin, L., Bissett, A. and Aitken, E.A. (2015). Green and brown bridges between weeds and crops reveal novel *Diaporthe* species in Australia. Persoonia 35, 39-49. doi: 10.3767/003158515X687506

Udayanga, D., Castlebury, L.A., Rossman, A.Y. and Hyde, K.D. (2014). Species limits in *Diaporthe*: molecular re-assessment of D. citri, *D. cytosporella*, *D. foeniculina* and *D. rudis*. Persoonia 32, 83-101. doi: 10.3767/003158514X679984

Voglmayr, H., Friebes, G., Gardiennet, A. and Jaklitsch, W.M. (2017). *Barrmaelia* and *Entosordaria* in Barrmaeliaceae (fam. nov., Xylariales), and critical notes on Anthostomella-like genera based on multi-gene phylogenies. Mycol. Prog. 17, 155–177. doi: 10.1007/s11557-017-1329-6

Vu, D., Groenewald, M., de Vries, M., Gehrmann, T., Stielow, B., Eberhardt, U., Al-Hatmi, A. et al. (2019) Large-scale generation and analysis of filamentous fungal DNA barcodes boosts coverage for kingdom fungi and reveals thresholds for fungal species and higher taxon delimitation. Stud. Mycol. 92, 135-154. doi: 10.1016/j.simyco.2018.05.001

Wendt, L.C., Kuhnert, E., Heitkaemper, S., Lambert, C., Hladki, A.I., Romero, A.I., Luangsa-ard, J.J., et al. (2018). Mycological Progress. 17, 115–154. doi: 10.1007/s11557-017-1311-3
